# Impact of endovascular revascularization, pharmacotherapy, and supervised exercise therapy on long-term cardiovascular, cerebrovascular, mortality, and limb outcomes in patients with peripheral artery disease: a systematic review and network meta-analysis

**DOI:** 10.3389/fmed.2026.1874951

**Published:** 2026-07-01

**Authors:** Jiayue Feng, Shang Ju, Jiawei Zhang

**Affiliations:** 1 Department of Peripheral Vascular Surgery, Beijing University of Chinese Medicine Affiliated Dongzhimen Hospital, Beijing, China; 2 Beijing University of Chinese Medicine, Beijing, China

**Keywords:** amputation, ankle-brachial index, endovascular revascularization, medical therapy, network meta-analysis, peripheral artery disease (PAD), supervised exercise therapy

## Abstract

**Objective:**

This systematic review and network meta-analysis compared the long-term effects of endovascular reconstruction/revascularization (ER), supervised exercise therapy (SET), medical therapy (MT), and their combined regimens on cardiovascular and cerebrovascular events, all-cause mortality, and limb outcomes in patients with peripheral artery disease (PAD).

**Methods:**

Chinese and English databases were searched from inception to April 8, 2026 for randomized controlled trials (RCTs) comparing ER, SET, MT, or their combinations in adults with PAD. The protocol was registered prospectively in PROSPERO (CRD420261362458). Risk of bias was assessed using the Cochrane RoB 2.0 tool. A frequentist network meta-analysis was performed in Stata 19.0. Odds ratios (ORs) were used for binary outcomes and mean differences (MDs) for continuous outcomes. Treatment ranking was summarized using the surface under the cumulative ranking curve (SUCRA), network consistency was evaluated by node-splitting, and certainty of evidence was assessed using the CINeMA framework.

**Results:**

Fifteen RCTs including 1,461 participants were eligible. For all-cause mortality, ER + SET + MT had the highest SUCRA value (99.4%), suggesting the greatest probability of benefit within the available evidence network. For cardiovascular events and cerebral infarction, ER + MT ranked highest, with SUCRA values of 76.7 and 85.3%, respectively. For amputation, SET + MT showed the highest SUCRA value (77.7%), indicating a greater probability of limb-salvage benefit. For ABI improvement, ER + SET + MT ranked highest (85.2%). Subgroup analyses suggested that the apparent amputation-protective effect of ER combined with ordinary medical therapy (ER + OMT) was larger than that of ER combined with best medical therapy (ER + BMT), although the direction of effect was consistent. Sensitivity analyses did not materially change the main rankings. Overall certainty of evidence was mainly moderate to low, largely because of clinical heterogeneity, sparse direct comparisons, limited reporting quality in some trials, and possible publication bias.

**Conclusion:**

In patients with PAD, ER + SET + MT may offer a favorable ranking profile for all-cause mortality and limb perfusion, ER + MT appears to rank favorably for cardiovascular and cerebrovascular events, and SET + MT may be particularly relevant for amputation prevention. These rankings should be interpreted cautiously because SUCRA values indicate relative ranking probability rather than definitive clinical superiority, and several comparisons were supported by limited direct evidence, heterogeneous populations, and moderate-to-low certainty. Further large, long-term, head-to-head RCTs are needed to confirm the optimal integrated treatment strategy for different PAD phenotypes.

## Introduction

Peripheral artery disease (PAD), also referred to in some studies as lower extremity arteriosclerosis obliterans, is a chronic progressive disease caused mainly by atherosclerosis (1). Its global prevalence has increased with population aging and with the rising burden of diabetes, smoking, dyslipidemia, and other atherosclerotic risk factors (2, 3). Clinically, PAD may present as intermittent claudication, rest pain, ischemic ulceration, or limb-threatening ischemia, and it can substantially reduce mobility and quality of life (4).

PAD is also a marker of systemic vascular disease. Patients with PAD have an elevated risk of myocardial infarction (5), stroke (6), and all-cause mortality (7). Long-term management therefore needs to address both limb-related outcomes and systemic cardiovascular risk. Current strategies include medical therapy (MT), supervised exercise therapy (SET), and endovascular reconstruction/revascularization (ER) (8). These treatments are intended to relieve ischemic symptoms, slow disease progression, improve walking capacity, and reduce cardiovascular, cerebrovascular, and limb events (9).

MT is the foundation of PAD care and commonly includes antiplatelet therapy, lipid-lowering therapy, and management of blood pressure, glycemia, smoking, and other risk factors. Best medical therapy (BMT) further emphasizes intensive lipid lowering, antithrombotic optimization, and comprehensive risk-factor control (10). SET, usually delivered as structured intermittent walking or aerobic exercise, improves walking performance and is recommended for symptomatic PAD, particularly intermittent claudication (11). In practice, however, uptake and adherence remain suboptimal (12). For patients with intermittent claudication, current guideline-based management generally prioritizes risk-factor modification, antithrombotic and lipid-lowering therapy, and structured or supervised exercise before revascularization when symptoms are not limb-threatening (13). ER, owing to its minimally invasive nature and shorter recovery time compared with open surgery, remains important for selected patients, particularly those with chronic limb-threatening ischemia or functionally limiting symptoms despite optimized conservative care (14).

Recent guideline recommendations provide an important clinical context for interpreting comparative effectiveness. The 2024 ESC guideline emphasizes long-term multidisciplinary management, preventive strategies, lifestyle modification, physical activity, and risk-factor control across the full PAD pathway (3). The 2024 ACC/AHA multisociety guideline similarly frames PAD care around guideline-directed medical therapy, structured exercise, and selective revascularization, with revascularization considered when functionally limiting claudication persists despite optimized medical and exercise therapy (9). Therefore, network rankings in this review were interpreted in relation to guideline-based sequencing rather than as a replacement for individualized clinical decision-making.

Although these interventions are widely used, direct comparisons among ER, SET, MT, and combined strategies remain fragmented. Most previous evidence has focused on single interventions or pairwise comparisons, and fewer studies have integrated hard long-term outcomes such as mortality, major cardiovascular and cerebrovascular events, and amputation. A network meta-analysis can synthesize both direct and indirect evidence and rank competing strategies across clinically relevant outcomes. We therefore conducted a systematic review and network meta-analysis of RCTs to compare ER, SET, MT, and their combinations in patients with PAD, with a focus on long-term cardiovascular, cerebrovascular, mortality, and limb outcomes.

## Materials and methods

### Protocol registration and reporting

The protocol was prospectively registered in the International Prospective Register of Systematic Reviews (PROSPERO; registration number CRD420261362458). The review was conducted and reported in accordance with the PRISMA extension for network meta-analysis (PRISMA-NMA) (15) and the methodological guidance of the Cochrane Collaboration (16).

### Literature search strategy

A comprehensive search was conducted in seven English-language databases (Embase, Scopus, Web of Science, Cochrane Library, Ovid, PubMed, and ProQuest) and six Chinese-language databases (DuXiu Academic, CNKI, Wanfang, VIP, SinoMed, and the Chinese Clinical Trial Registry) from database inception to April 8, 2026. Medical Subject Headings and free-text terms were combined and adapted to each database. Core terms included “peripheral arterial disease,” “arteriosclerosis obliterans,” “intermittent claudication,” “angioplasty,” “stent,” “supervised exercise,” “medical therapy,” and “randomized controlled trial.” Boolean operators were used to maximize sensitivity and specificity. The full search strategies should be provided as a Supporting Information file.

### Eligibility criteria

Studies were eligible if they met the following criteria: (1) RCTs published in English or Chinese, regardless of blinding; (2) adult patients aged 18 years or older with a confirmed diagnosis of PAD based on ABI, duplex ultrasound, computed tomography angiography, magnetic resonance angiography, or other accepted vascular imaging methods; (3) comparisons involving ER, MT, SET, or combinations of these interventions, with follow-up of at least 6 months; and (4) reporting at least one core safety or prognostic outcome, including all-cause mortality, amputation, or major cardiovascular/cerebrovascular events. Efficacy outcomes such as ABI, maximal walking distance, and pain-free walking distance were considered when available. Studies were excluded if they were duplicate publications, lacked extractable data, had unavailable full text, or did not provide sufficient information after attempts to obtain missing data from authors or Supplementary material.

### Study selection and data extraction

Two reviewers independently screened titles, abstracts, and full texts. Search results were imported into NoteExpress 3.7 for deduplication. Records that were clearly irrelevant were removed after title and abstract screening, and potentially eligible reports were assessed in full text against the prespecified criteria. Disagreements were resolved by discussion or by consultation with a third reviewer. The selection process was summarized in a PRISMA flow diagram.

Data were extracted independently by two reviewers using a standardized form. Extracted information included study characteristics, first author, publication year, country, sample size, follow-up duration, patient characteristics, clinical presentation, PAD severity when reported, lesion location, comorbidities, baseline ABI, intervention details, medication intensity, SET protocol, outcome definitions, measurement time points, effect estimates, missing data handling, randomization procedures, allocation concealment, blinding, and attrition. We specifically compared trial-level characteristics relevant to the transitivity assumption, including intermittent claudication versus more advanced PAD, lesion distribution, ER technique, medication intensity, exercise supervision, and follow-up time. Discrepancies were checked against the source reports and resolved by consensus.

Outcome harmonization was performed before analysis. Cardiovascular events were extracted according to each trial’s prespecified definition, usually including myocardial infarction, unstable angina, cardiovascular hospitalization, or other major cardiovascular complications when reported. Cerebral infarction, all-cause mortality, amputation, and ABI were extracted as reported. Because definitions of cardiovascular events and amputation were not fully uniform across trials, we avoided reclassifying outcomes beyond the information available in the original reports and treated this variation as a source of clinical heterogeneity and indirectness.

### Risk of bias assessment

Risk of bias in the included RCTs was assessed using the Cochrane Risk of Bias 2.0 (RoB 2.0) tool (17). The following domains were evaluated: bias arising from the randomization process, bias due to deviations from intended interventions, bias due to missing outcome data, bias in outcome measurement, and bias in selection of the reported result. Each domain was judged as “low risk,” “some concerns,” or “high risk.” Two reviewers completed the assessment independently, and disagreements were resolved through discussion or third-party adjudication. For transparency, we summarized both domain-level and overall judgments, including the number of studies judged as low risk, some concerns, or high risk in each RoB 2.0 domain.

### Statistical analysis

The statistical analysis followed PRISMA-NMA and Cochrane recommendations (15, 16). Binary outcomes, including all-cause mortality, cardiovascular events, cerebral infarction, and amputation, were summarized using odds ratios (ORs) with 95% confidence intervals (CIs). The continuous ABI outcome was summarized using mean differences (MDs) with 95% CIs. When necessary, effect estimates were calculated or converted from the available study-level data.

Pairwise heterogeneity was evaluated using the I2 statistic. A fixed-effect model was used when heterogeneity was low or moderate (I2 = 50%), and a random-effects model was used when substantial heterogeneity was present (I2 > 50%). Network meta-analysis was performed within a frequentist framework in Stata 19.0. Network geometry was visualized with evidence network plots, in which node size reflects the number of participants and edge thickness reflects the amount of direct comparative evidence. Consistency between direct and indirect evidence was examined using the node-splitting method; *P* > 0.05 was interpreted as no statistically detectable inconsistency. Relative treatment rankings were summarized using SUCRA values, where values closer to 100% suggest a higher probability of being among the better-ranked treatments. SUCRA results were interpreted as ranking probabilities rather than direct proof of superiority.

Publication bias and small-study effects were explored using comparison-adjusted funnel plots. Sensitivity analyses were conducted by excluding individual studies sequentially, by reassessing potentially heterogeneous or influential studies, and, where the available data permitted, by examining medication intensity and follow-up duration. A sensitivity analysis excluding higher-risk or methodologically less clearly reported studies was also considered; where the network became too sparse to support a stable estimate, this was reported as a limitation rather than used for overinterpretation. Certainty of evidence for each outcome was assessed using the CINeMA framework (18) and graded as high, moderate, low, or very low.

### Intervention node definition and transitivity rationale

The original reports included four core intervention classes: ordinary medical therapy (OMT), best medical therapy (BMT), endovascular reconstruction/revascularization (ER), and supervised exercise therapy (SET). These corresponded to six specific regimens: OMT alone, BMT alone, ER + OMT, ER + BMT, SET + BMT, and ER + SET + BMT. To construct a clinically interpretable and statistically stable evidence network, OMT and BMT were merged into a single MT node, yielding four final nodes: MT, ER + MT, SET + MT, and ER + SET + MT. This decision was made before interpretation of treatment rankings because separate OMT and BMT nodes would have produced a fragmented network with sparse or disconnected comparisons for several outcomes.

This merging was based on clinical and methodological considerations. OMT and BMT share the same foundational therapeutic objectives in PAD: antiplatelet therapy, lipid lowering, risk-factor modification, and prevention of systemic atherosclerotic events (3, 9). Their main difference lies in treatment intensity, target achievement, and implementation rather than in therapeutic mechanism. Subgroup analyses comparing ER + OMT and ER + BMT showed a broadly consistent direction of effect, supporting the clinical acceptability of a combined MT node for the primary network. Nevertheless, the merged MT node may dilute differences between less intensive and optimized pharmacotherapy, and the resulting rankings should not be interpreted as showing the effect of a uniform medication package. Subgroup analyses by medication intensity were therefore retained to test whether the magnitude of treatment effects differed between OMT and BMT.

The transitivity assumption was evaluated by comparing patient populations, clinical presentation, baseline ABI, comorbidities, lesion location, follow-up duration, outcome definitions, and intervention characteristics across studies. The included trials all enrolled patients with clinically confirmed PAD or intermittent claudication and reported vascular or limb-related outcomes relevant to PAD management. However, clinical heterogeneity remained important: some trials focused mainly on intermittent claudication, whereas others included patients with more advanced arterial occlusive disease; ER techniques, lesion sites, SET protocols, medication intensity, and follow-up duration also varied. Node-splitting analyses did not show statistically significant inconsistency (*P* > 0.05), but absence of statistical inconsistency does not eliminate concerns about clinical transitivity. We therefore interpreted indirect comparisons cautiously and emphasized uncertainty when evidence was sparse or heterogeneous.

Because subgroup separation by disease severity or clinical presentation was not feasible for all outcomes, we did not perform a complete disease-severity network. Instead, we summarized available severity-related information in the study characteristics table and considered disease phenotype, lesion location, follow-up duration, and intervention implementation when interpreting transitivity and certainty.

## Results

### Literature search results

**FIGURE 1:**
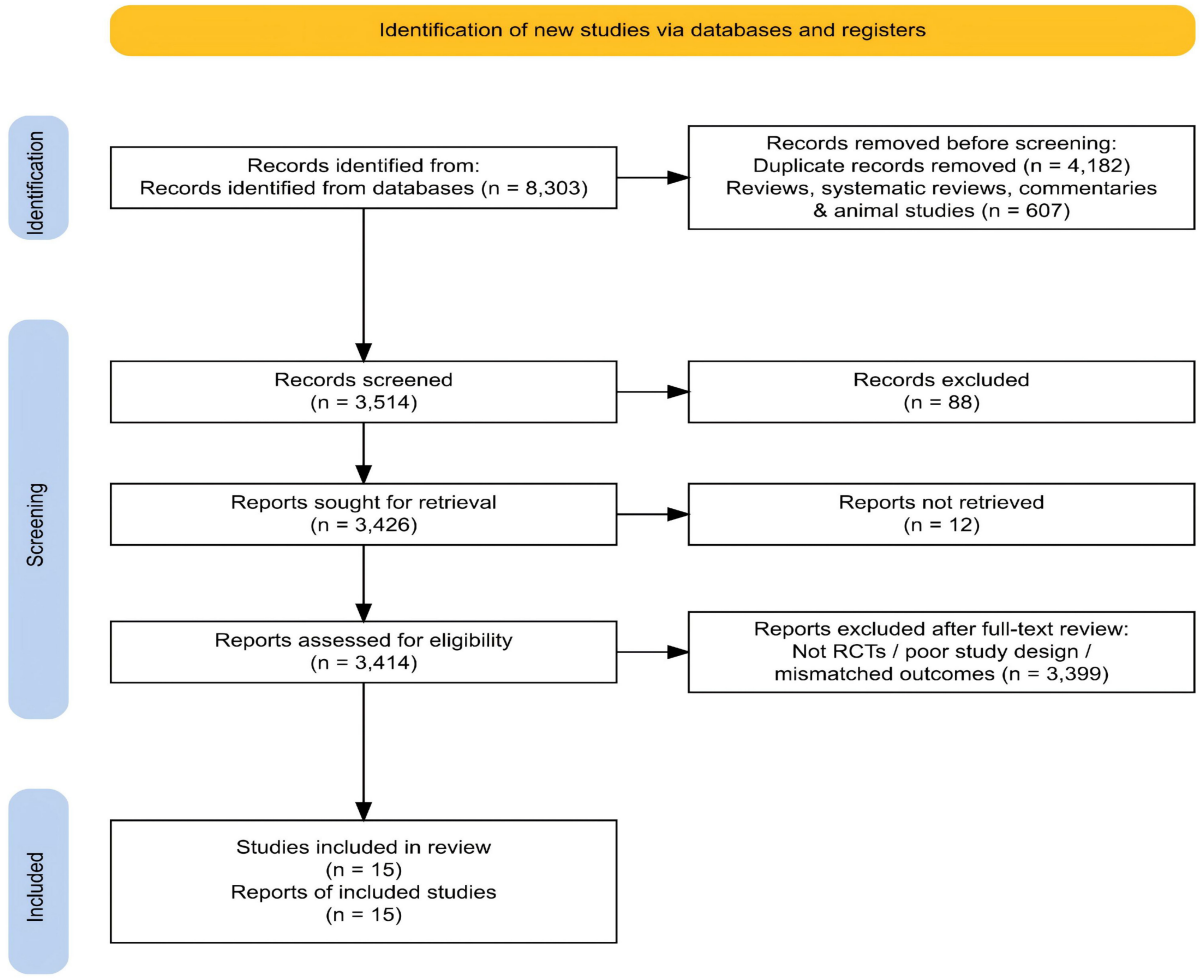
Literature search and study selection flow diagram. RCT, randomized controlled trial.

The search identified 8,303 records, and no additional records were identified from other sources. After deduplication in NoteExpress 3.7, 4,121 unique records remained. Of these, 607 reviews, systematic reviews, meta-analyses, and animal studies were excluded. A further 88 records were removed after title and abstract screening. Among 3,414 records assessed in full text, 12 were excluded because the full text was unavailable, and 3,399 were excluded because they were not RCTs, had unsuitable designs, or did not report relevant outcomes. Ultimately, 15 RCTs met the eligibility criteria and were included in the network meta-analysis. The study selection process is shown in Figure 1.

### Characteristics of included studies

The 15 eligible RCTs were published between 2013 and 2023 and included 1,461 patients with PAD or intermittent claudication. Baseline characteristics, including age, sex distribution, comorbidities, and ABI, were generally comparable between study arms within individual trials. Across trials, however, there was clinically relevant variation in disease severity, lesion location, intervention technique, medication intensity, SET protocol, and follow-up duration. The studies evaluated OMT, BMT, ER, SET, and their combined strategies. For network construction, OMT and BMT were merged into the MT node, as described above. Twelve studies compared ER + MT with MT alone (19, 20), two compared ER + MT with SET + MT (21, 22), and one compared ER + SET + MT with SET + MT (23). The baseline characteristics of the included studies are summarized in Table 1, and these characteristics were used to judge the plausibility of transitivity.

**TABLE 1:** Baseline characteristics of included studies.

Author reference (trial name)	Country	Patient population	EG n	CG n	Age EG	Age CG	Male EG (%)	Male CG (%)	Intervention EG	Intervention CG	Outcomes
Fakhry et al. (21)	Netherlands	IC (>= 3 months) with iliac or femoral stenosis	75	75	65 ± 11.4	66 ± 9.1	58.7	52.0	ER + BMT	SET + BMT	1,2,3
Kong (19)	China	ASO, ABI <= 0.9	78	78	62.3 ± 13.7	Not clear	60.0	Not clear	ER + OMT	OMT	1, 2, 3, 4, 5, 6
Weiming et al. (27)	China	ASO, iliac or femoral stenosis > 50%	41	41	63.24 ± 5.40	62.98 ± 5.31	70.7	65.9	ER + BMT	BMT	2, 3, 4, 5, 6
Huiliang et al. (28)	China	ASO, ABI < 0.9, arterial stenosis = 50%	51	51	68.3 ± 7.4	68.5 ± 7.2	62.7	58.8	ER + OMT	OMT	1, 2
Longhu et al. (29)	China	ASO, ABI < 0.9, arterial stenosis > 50%	115	65	68.53 ± 8.96	Not clear	60.6	Not clear	ER + BMT	BMT	1, 2, 3, 6
Dong (30)	China	ASO	45	45	58.2 ± 5.1	57.9 ± 4.6	60.0	64.4	ER + OMT	OMT	3
Zhang et al. (24)	China	ASO	30	30	70.7 ± 5.2	71.1 ± 5.9	66.7	60.0	ER + OMT	OMT	1, 2, 3, 4, 5, 6
Zhang et al. (25)	China	ASO, ABI < 0.9, arterial stenosis >50%	34	34	64.1 ± 5.2	64.3 ± 5.1	55.9	58.8	ER + OMT	OMT	2, 3, 5, 6
Jiao (31)	China	ASO, arterial stenosis > = 50%	39	39	73.02 ± 5.27	72.35 ± 5.21	61.5	59.0	ER + OMT	OMT	1, 2, 3, 4, 5, 6
Wu et al. (32)	China	ASO	30	30	66.1 ± 12.2	67.3 ± 12.5	63.3	56.7	ER + OMT	OMT	1, 2, 3, 4, 5, 6
Dong and Zhang (33)	China	ASO	90	90	Not clear	Not clear	64.4	Not clear	ER + OMT	OMT	3
Koelemay et al. (22)	Netherlands	IC with iliac stenosis > 50%	114	126	61 ± 9	63 ± 8	55.3	65.9	ER + BMT	SET + BMT	2, 3, 4, 5
Klaphake et al. (23)	Netherlands	IC (= 3 months) with iliac or femoral stenosis	106	106	64 ± 9	66 ± 10	56.6	68.0	ER + SET + BMT	SET + BMT	1, 2, 3
Liu (26)	China	ASO	30	30	62.55 ± 5.74	62.41 ± 5.96	56.7	53.3	ER + OMT	OMT	1, 2, 3, 4, 5, 6
Gunnarsson et al. (20)	Sweden	IC ( > = 6 months) with femoral stenosis; treadmill walking distance < 500 m	48	52	71.3 ± 5.3	69.8 ± 5.8	45.8	53.8	ER + BMT	BMT	2, 3

IC, intermittent claudication; EG, experimental group; CG, control group; sten., stenosis; Outc., outcome; ER, endovascular revascularization; OMT, ordinary medical therapy; BMT, best medical therapy; SET, supervised exercise therapy; ASO, arteriosclerosis obliterans; TWD, treadmill walking distance; 1, ABI; 2, mortality rate; 3, amputation rate; 4, cardiovascular event incidence; 5, cerebral infarction incidence; 6, cerebral hemorrhage incidence.

### Risk of bias in included studies

**FIGURE 2:**
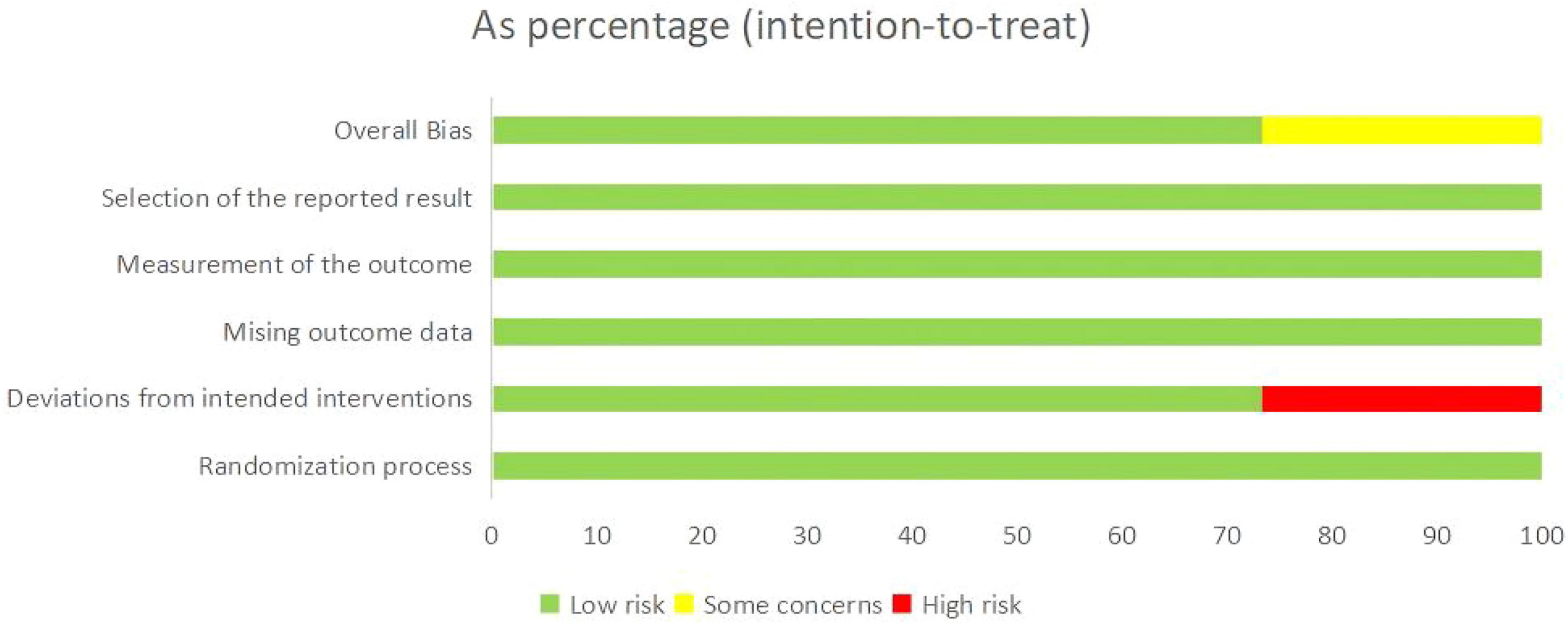
Risk of bias summary for included studies assessed using the Cochrane risk of Bias tool, version 2.0 (RoB 2.0).

Risk-of-bias assessment with RoB 2.0 showed that several studies reported adequate random sequence generation, including random number tables, computer-generated sequences, or sealed envelopes (19–26). Other studies described randomization but provided limited procedural details (27–33), resulting in some concerns for the randomization domain. Overall, most studies were judged as low risk for missing outcome data, outcome measurement, and selection of the reported result because the main outcomes were objective and follow-up reporting was generally complete. The main methodological concern was bias due to deviations from intended interventions, particularly where implementation bias, crossover, incomplete adherence to SET, or incomplete medication adherence could not be excluded. In summary, approximately half of the trials provided clear randomization details, most outcome-measurement and missing-data domains were judged low risk, and a smaller subset had concerns related to treatment implementation. The risk-of-bias summary is presented in Figure 2.

### Network meta-analysis results

#### All-cause mortality

**FIGURE 3:**
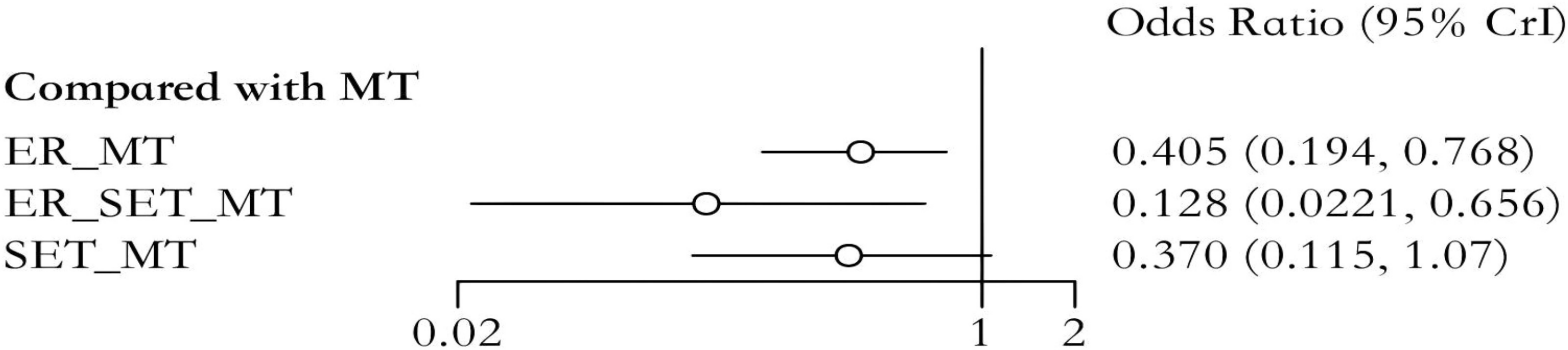
Forest plot of network meta-analysis for all-cause mortality, with MT as the common control. OR < 1 indicates lower mortality risk in the intervention group. ER + MT, endovascular revascularization plus medication; ER + SET + MT, endovascular revascularization plus supervised exercise plus medication; SET + MT, supervised exercise plus medication; MT, medication.

**FIGURE 4:**
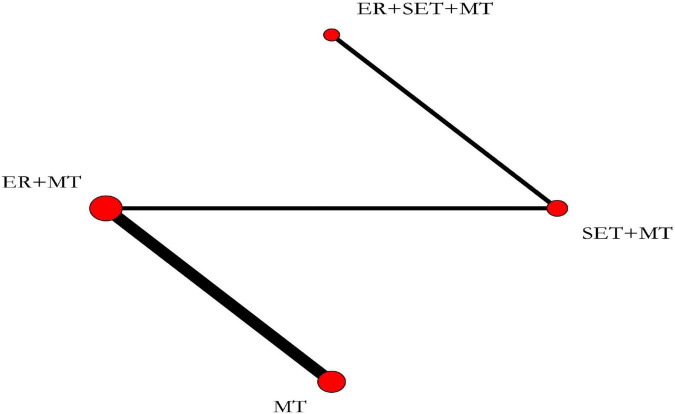
Network plot for all-cause mortality. Node size is proportional to sample size, and line thickness is proportional to the number of studies.

Thirteen studies (19–29, 31, 32) reported all-cause mortality and contributed to a network containing four interventions: MT, ER + MT, SET + MT, and ER + SET + MT. The forest plot with MT as the reference is shown in Figure 3. The network plot (Figure 4) shows that ER + MT had the largest sample size and that the MT versus ER + MT comparison provided the greatest amount of direct evidence.

**FIGURE 5:**
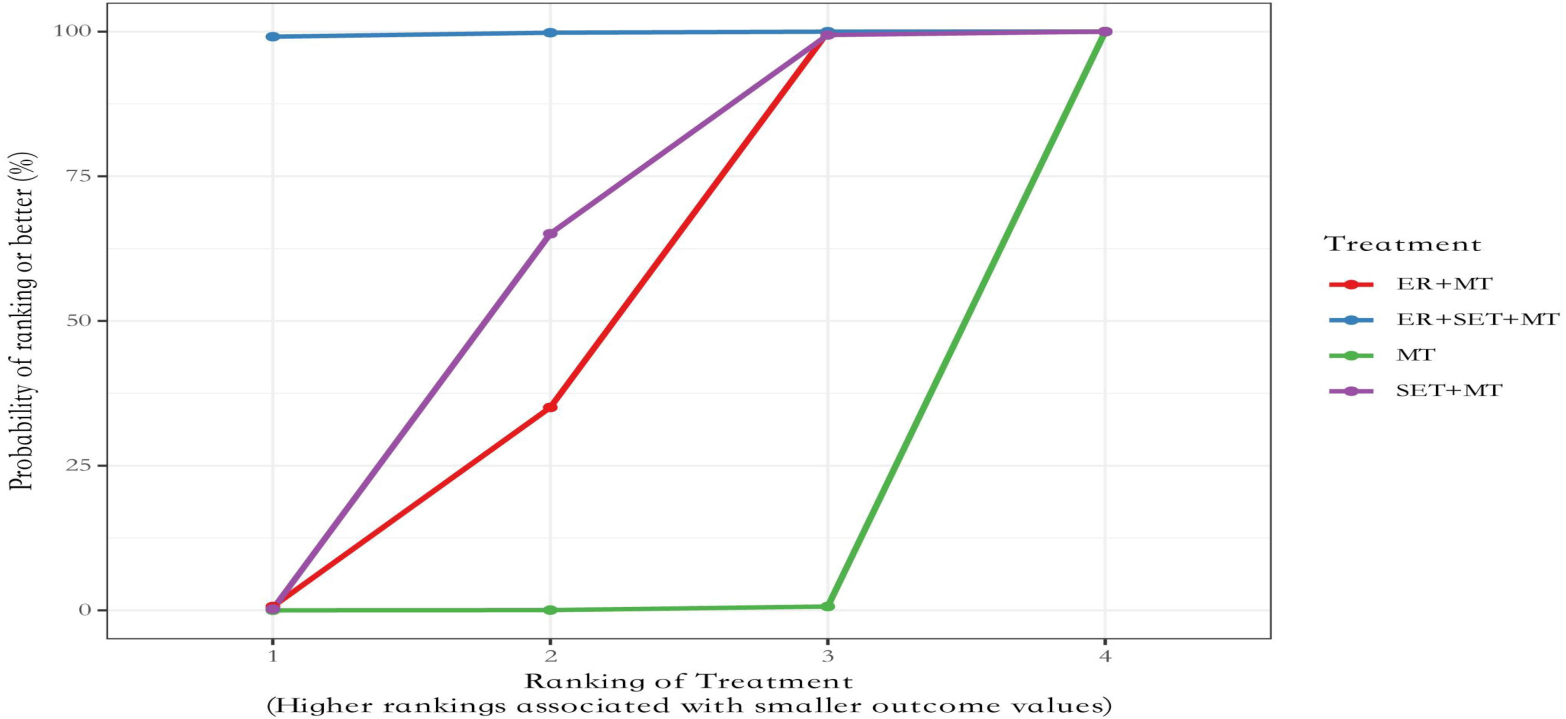
Ranking probability plot for all-cause mortality. Higher rankings are associated with lower outcome values.

**FIGURE 6:**
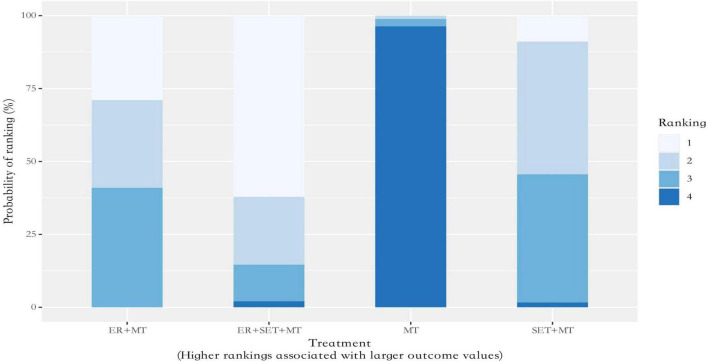
Ranking probability distribution for all-cause mortality. Lighter colors represent higher treatment rankings and lower mortality risk.

Heterogeneity was low (I2 = 8.9%, *P* = 0.356), and node-splitting analysis showed no statistically significant inconsistency (*P* > 0.05). A consistency fixed-effect model was therefore used. SUCRA rankings for reducing all-cause mortality were ER + SET + MT (99.64%) > SET + MT (54.90%) > ER + MT (45.22%) > MT (0.23%). This ranking suggests that ER + SET + MT occupied the most favorable position for mortality reduction in the available network, whereas MT alone ranked lowest. Because the ER + SET + MT node was supported by limited direct evidence, this ranking should be interpreted as a probability signal rather than definitive proof of superiority. Pairwise network estimates are summarized in Table 2, and rank probability plots are shown in Figures 5, 6.

**TABLE 2:** League table for all-cause mortality.

ER_MT	ER_SET_MT	MT	SET_MT
ER_MT	0.318 (0.065, 1.510)	2.482 (1.300, 5.148)	0.912 (0.375, 2.246)
3.149 (0.662, 15.497)	ER_SET_MT	7.811 (1.504, 46.188)	2.861 (0.801, 10.759)
0.403 (0.194, 0.769)	0.128 (0.022, 0.665)	MT	0.369 (0.114, 1.081)
1.097 (0.445, 2.665)	0.350 (0.093, 1.249)	2.710 (0.925, 8.785)	SET_MT

Values are odds ratios with 95% confidence intervals.

**FIGURE 7:**
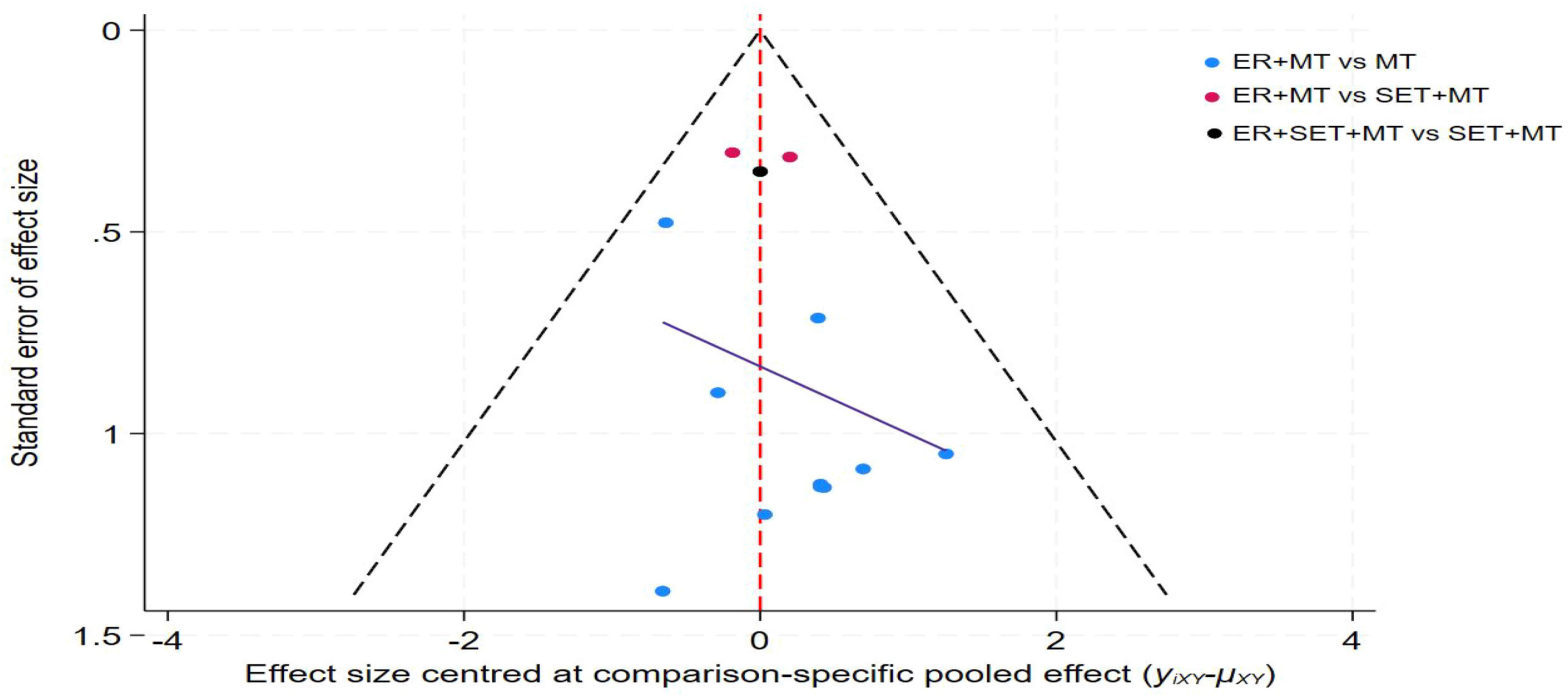
Comparison-adjusted funnel plot for all-cause mortality. Asymmetry in effect-size distribution and an angled regression line suggest possible publication bias or small-study effects.

The comparison-adjusted funnel plot showed asymmetry in effect-size distribution and a tilted regression line (Figure 7), suggesting possible publication bias or small-study effects. Sequential sensitivity analyses did not materially alter the main ranking. After excluding three studies with potential heterogeneity concerns (20, 26, 28), SUCRA rankings remained directionally consistent with the primary analysis. Subgroup analysis by medication intensity for the ER + MT versus MT comparison showed a protective direction in both the BMT subgroup (I2 = 76%, *P* = 0.03; OR = 0.47, 95% CI: 0.24–0.94, *P* = 0.02) and the OMT subgroup (I2 = 78%, *P* < 0.0001; OR = 0.38, 95% CI: 0.24–0.62, *P* = 0.0001), with no statistically significant subgroup difference (I2 = 0%, *P* = 0.63). Subgroup analysis by follow-up duration showed similar protective directions in studies with = 6 months of follow-up (I2 = 75%, *P* = 0.008; OR = 0.40, 95% CI: 0.23–0.73, *P* = 0.003) and > 6 months of follow-up (I2 = 44%, *P* = 0.17; OR = 0.42, 95% CI: 0.27–0.66, *P* = 0.0002).

#### Cardiovascular event incidence

**FIGURE 8:**
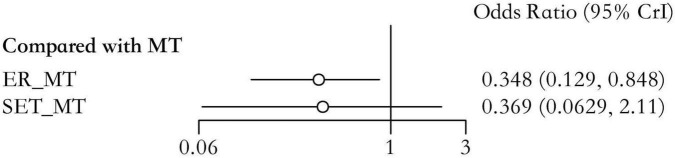
Forest plot of network meta-analysis for cardiovascular event incidence, with MT as the common control. OR < 1 indicates lower cardiovascular event risk in the intervention group. ER + MT, endovascular revascularization plus medication; SET + MT, supervised exercise plus medication; MT, medication.

**FIGURE 9:**
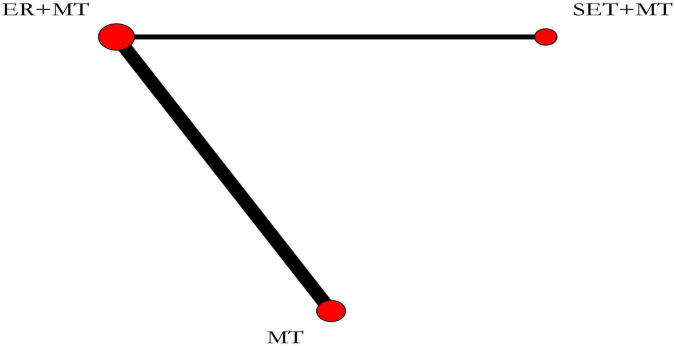
Network plot for cardiovascular event incidence. Node size is proportional to sample size, and line thickness is proportional to the number of studies.

Seven studies (19, 22, 24, 26, 27, 31, 32) reported cardiovascular event incidence and contributed to a three-node network including MT, ER + MT, and SET + MT. The forest plot with MT as the reference is shown in Figure 8, and the network plot is shown in Figure 9. ER + MT had the largest node and the MT versus ER + MT comparison formed the core evidence link.

**FIGURE 10:**
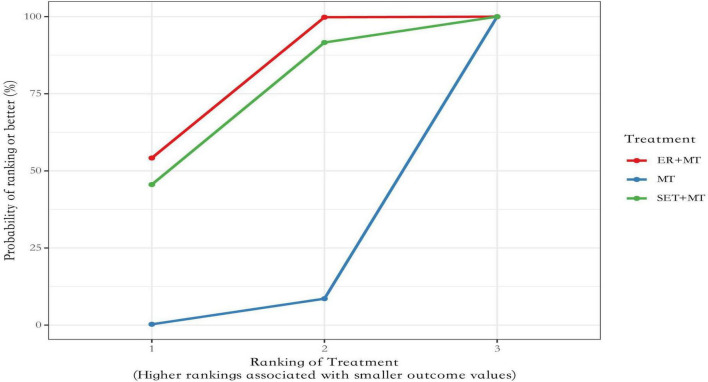
Ranking probability plot for cardiovascular event incidence. Higher rankings are associated with lower cardiovascular event risk.

**FIGURE 11:**
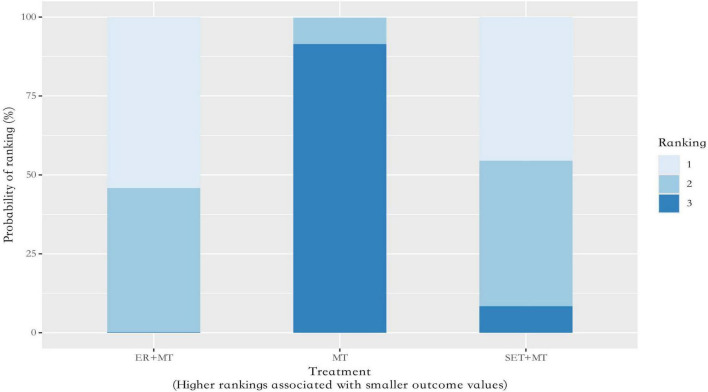
Ranking probability distribution for cardiovascular event incidence. Lighter colors represent higher treatment rankings and lower cardiovascular event risk.

Heterogeneity was negligible (I2 = 0.0%, *P* = 0.803), and node-splitting analysis indicated no statistically significant inconsistency (*P* > 0.05). A consistency fixed-effect model was used. SUCRA rankings for reducing cardiovascular events were ER + MT (77.00%) > SET + MT (68.59%) > MT (4.41%), suggesting that ER + MT ranked most favorably for this outcome within the observed network. This ranking should be interpreted alongside the limited number of contributing trials and the possibility that background medication intensity and baseline cardiovascular risk differed across studies. The league table is provided in Table 3. Cumulative ranking and rank probability distributions are shown in Figures 10, 11.

**TABLE 3:** League table for cardiovascular event incidence.

ER_MT	MT	SET_MT
ER_MT	2.890 (1.183, 7.892)	1.060 (0.241, 4.841)
0.346 (0.127, 0.845)	MT	0.364 (0.062, 2.138)
0.943 (0.207, 4.146)	2.747 (0.468, 16.151)	SET_MT

Values are odds ratios with 95% confidence intervals.

**FIGURE 12:**
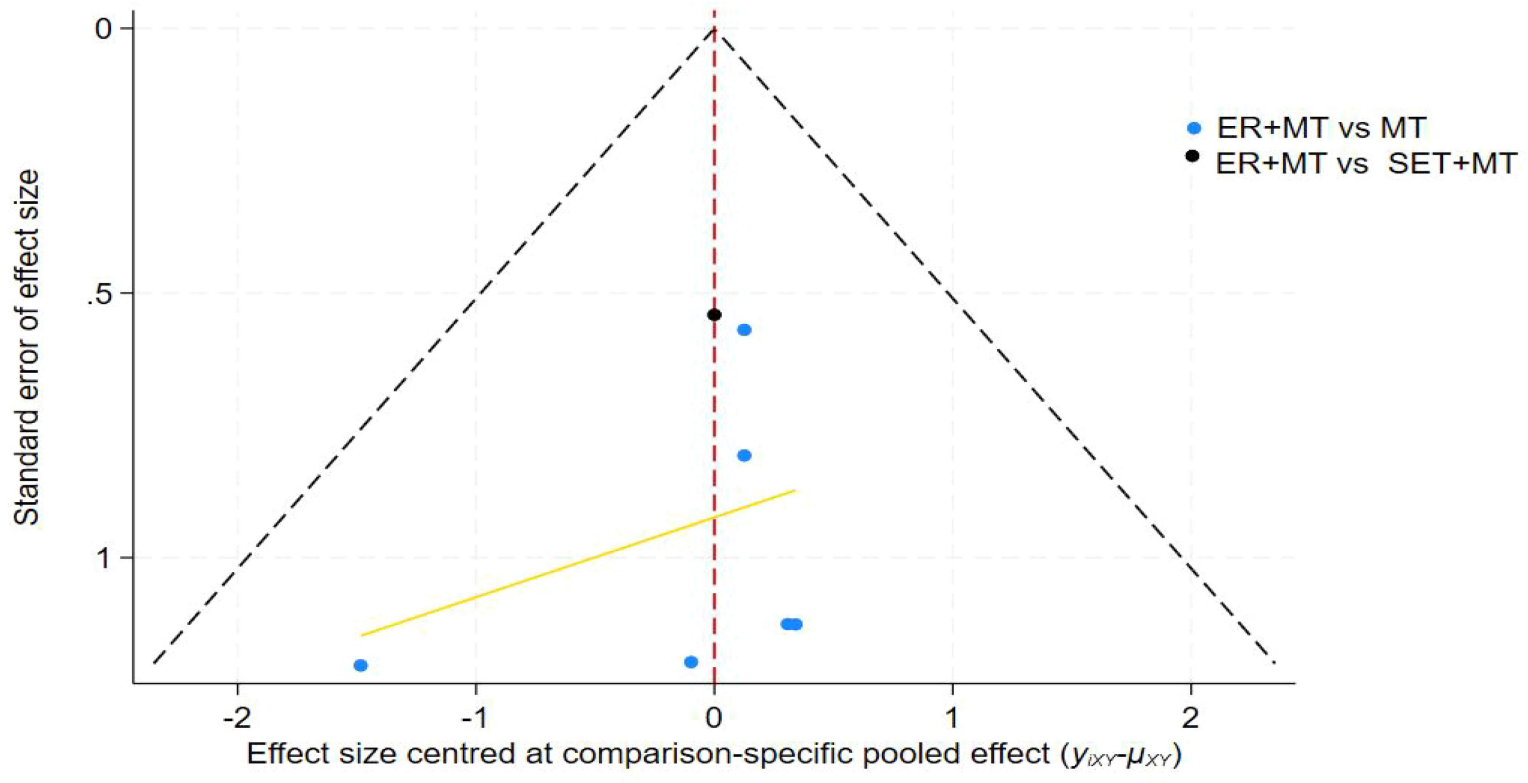
Comparison-adjusted funnel plot for cardiovascular event incidence. Asymmetrical clustering and a tilted regression line suggest possible publication bias or small-study effects.

The comparison-adjusted funnel plot showed asymmetrical clustering of effect points and a tilted regression line (Figure 12), suggesting possible publication bias or small-study effects. Sensitivity analysis excluding studies with a SET + MT comparator and subsequently removing one study with potential heterogeneity concerns (31) reduced heterogeneity from I2 = 81 to 0%. The recalculated pooled effect estimate (OR = 0.39, 95% CI: 0.30–0.50) and repeated network meta-analysis remained directionally consistent with the primary analysis. A subgroup analysis limited to OMT studies after excluding one BMT study showed I2 = 0% and a protective effect for ER + MT (OR = 0.38, *P* < 0.00001). Subgroup analysis by follow-up duration showed consistent effects for = 6 months (I2 = 0%, *P* = 0.50; OR = 0.38, 95% CI: 0.27–0.53, *P* < 0.00001) and > 6 months (I2 = 0%, *P* = 1.00; OR = 0.40, 95% CI: 0.26–0.61, *P* < 0.0001).

#### Cerebral infarction incidence

**FIGURE 13:**
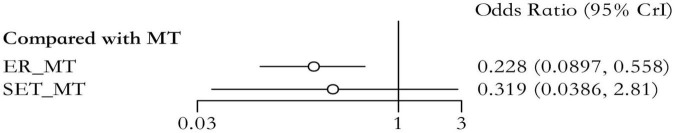
Forest plot of network meta-analysis for cerebral infarction incidence, with MT as the common control. OR < 1 indicates lower cerebral infarction risk in the intervention group. ER + MT, endovascular revascularization plus medication; SET + MT, supervised exercise plus medication; MT, medication.

**FIGURE 14:**
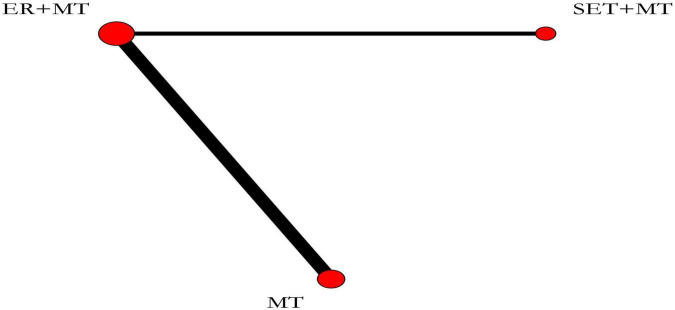
Network plot for cerebral infarction incidence. Node size is proportional to sample size, and line thickness is proportional to the number of studies.

Nine studies (19, 22, 24–27, 29, 31, 32) reported cerebral infarction incidence and formed a three-node network of MT, ER + MT, and SET + MT. The forest plot (Figure 13) and network plot (Figure 14) again showed ER + MT as the largest node and the MT versus ER + MT comparison as the principal direct evidence link.

**FIGURE 15:**
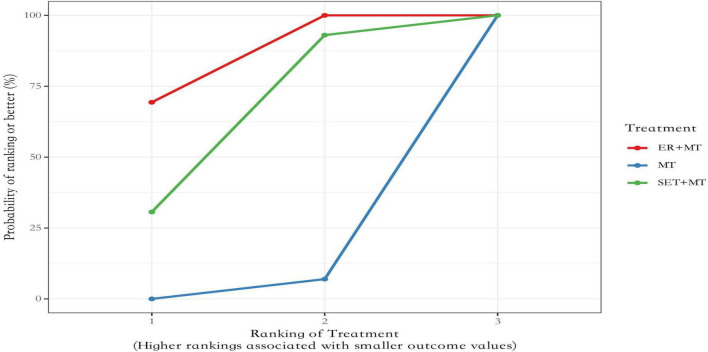
Ranking probability plot for cerebral infarction incidence. Higher rankings are associated with lower cerebral infarction risk.

**FIGURE 16:**
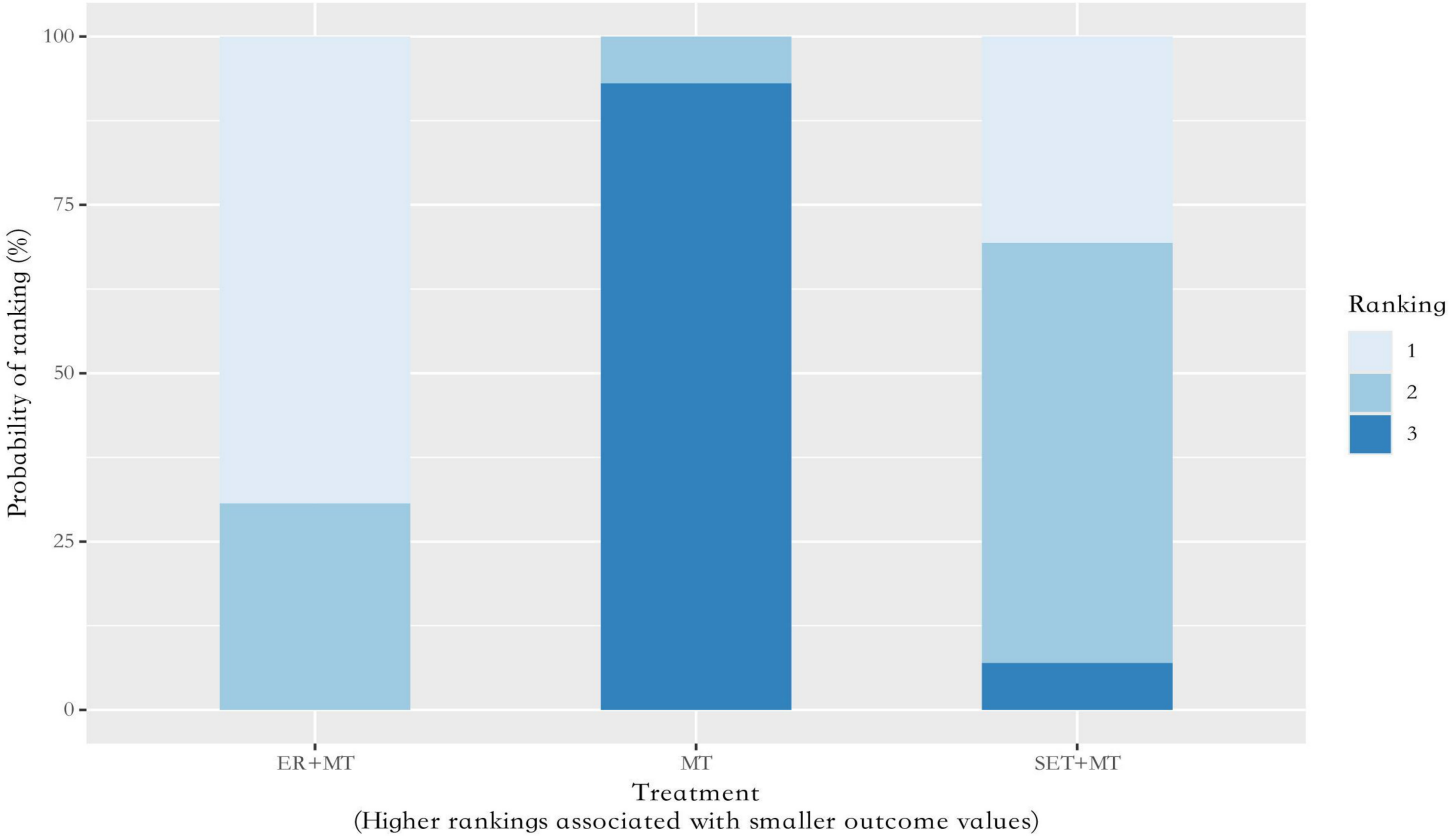
Ranking probability distribution for cerebral infarction incidence. Lighter colors represent higher treatment rankings and lower cerebral infarction risk.

Heterogeneity was low (I2 = 7.7%, *P* = 0.371), and node-splitting analysis showed no significant inconsistency (*P* > 0.05). A consistency fixed-effect model was used. SUCRA rankings for reducing cerebral infarction risk were ER + MT (84.67%) > SET + MT (61.85%) > MT (3.48%). ER + MT had a 70.6% probability of being the best-ranked strategy and a mean rank of 1.3. These results indicate a favorable ranking signal for ER + MT but should not be interpreted as conclusive evidence of superiority because several comparisons were informed mainly by indirect evidence. Pairwise estimates are shown in Table 4, and ranking plots are presented in Figures 15, 16.

**TABLE 4:** League table for cerebral infarction incidence.

ER_MT	MT	SET_MT
ER_MT	4.414 (1.764, 11.012)	1.425 (0.213, 10.108)
0.227 (0.091, 0.567)	MT	0.321 (0.039, 2.841)
0.702 (0.099, 4.703)	3.112 (0.352, 25.497)	SET_MT

Values are odds ratios with 95% confidence intervals.

**FIGURE 17:**
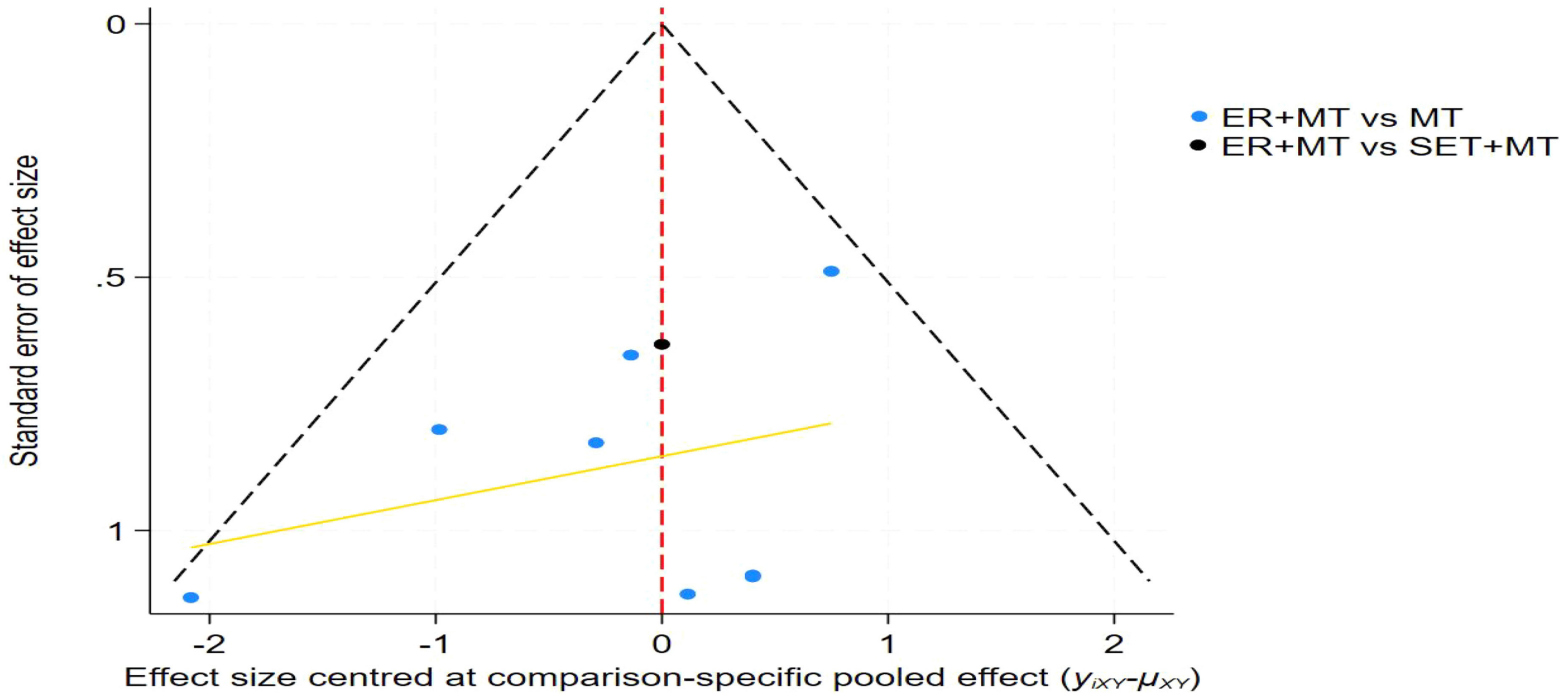
Comparison-adjusted funnel plot for cerebral infarction incidence. A slight asymmetry and angled regression line suggest possible publication bias.

The comparison-adjusted funnel plot showed points largely within the 95% confidence limits, but a slight asymmetry and an angled regression line suggested possible publication bias (Figure 17). Sequential sensitivity analyses did not materially change the conclusions. Excluding two studies identified as possible sources of heterogeneity (19, 31) reduced I2 from 89 to 40%, and SUCRA rankings remained consistent with the main analysis. Subgroup analysis by medication intensity showed a protective direction in both the BMT subgroup (I2 = 77%, *P* = 0.04; OR = 0.28, 95% CI: 0.12–0.65, *P* = 0.003) and the OMT subgroup (I2 = 18%, *P* = 0.30; OR = 0.32, 95% CI: 0.23–0.44, *P* < 0.00001), with no significant subgroup interaction (I2 = 0%, *P* = 0.75). Subgroup analysis by follow-up duration also showed consistent effects in < = 6 months (I2 = 27%, *P* = 0.25; OR = 0.35, 95% CI: 0.23–0.52, *P* < 0.00001) and > 6 months (I2 = 77%, *P* = 0.04; OR = 0.28, 95% CI: 0.12–0.65, *P* = 0.003).

#### Amputation incidence

**FIGURE 18:**
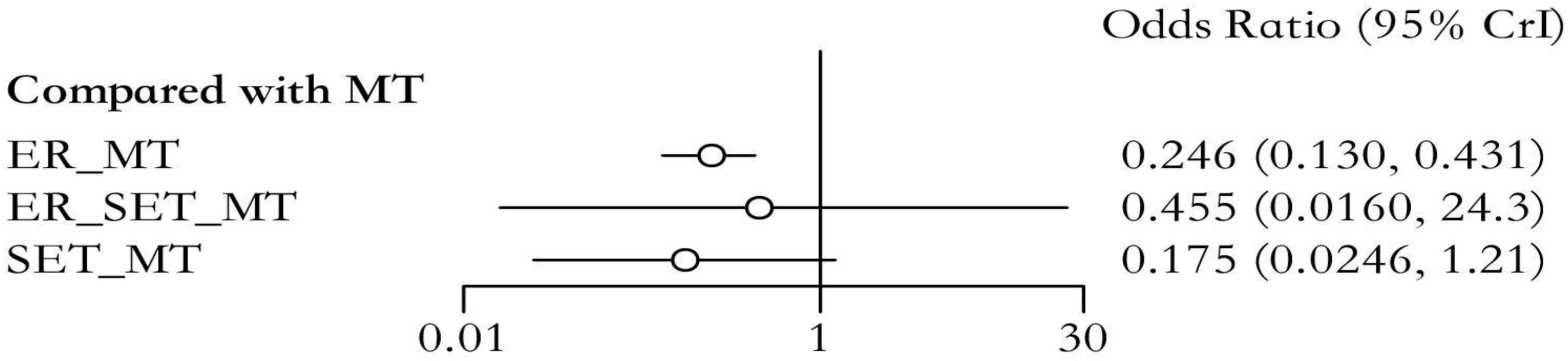
Forest plot of network meta-analysis for amputation incidence, with MT as the common control. OR < 1 indicates lower amputation risk in the intervention group. ER + MT, endovascular revascularization plus medication; ER + SET + MT, endovascular revascularization plus supervised exercise plus medication; SET + MT, supervised exercise plus medication; MT, medication.

**FIGURE 19:**
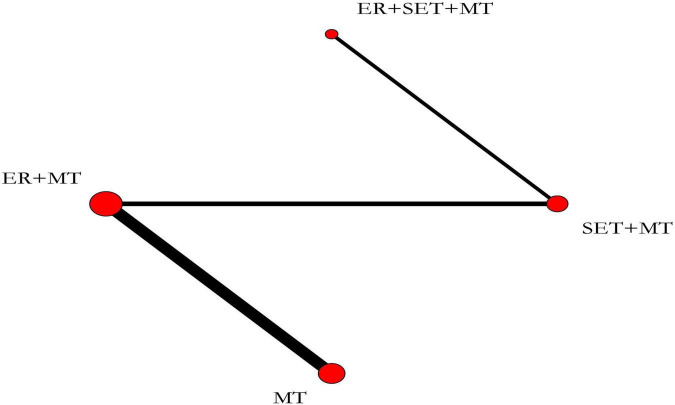
Network plot for amputation incidence. Node size is proportional to sample size, and line thickness is proportional to the number of studies.

Fourteen studies (19–27, 29–33) reported amputation incidence and contributed to a four-node network including MT, ER + MT, SET + MT, and ER + SET + MT. The forest plot is shown in Figure 18. The network plot (Figure 19) showed a connected structure, with the ER + MT versus MT comparison forming the core link.

**FIGURE 20:**
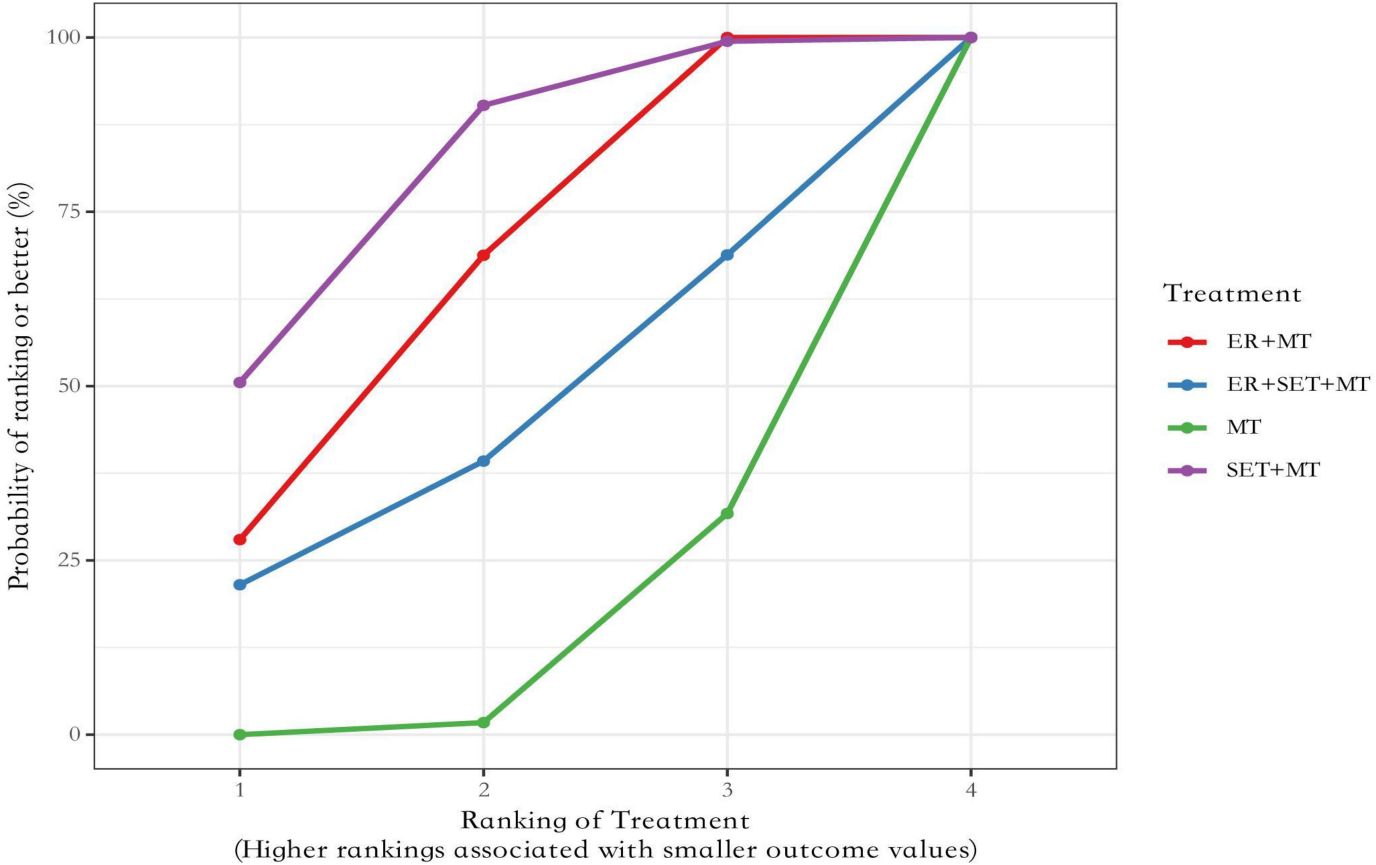
Ranking probability plot for amputation incidence. Higher rankings are associated with lower amputation risk.

**FIGURE 21:**
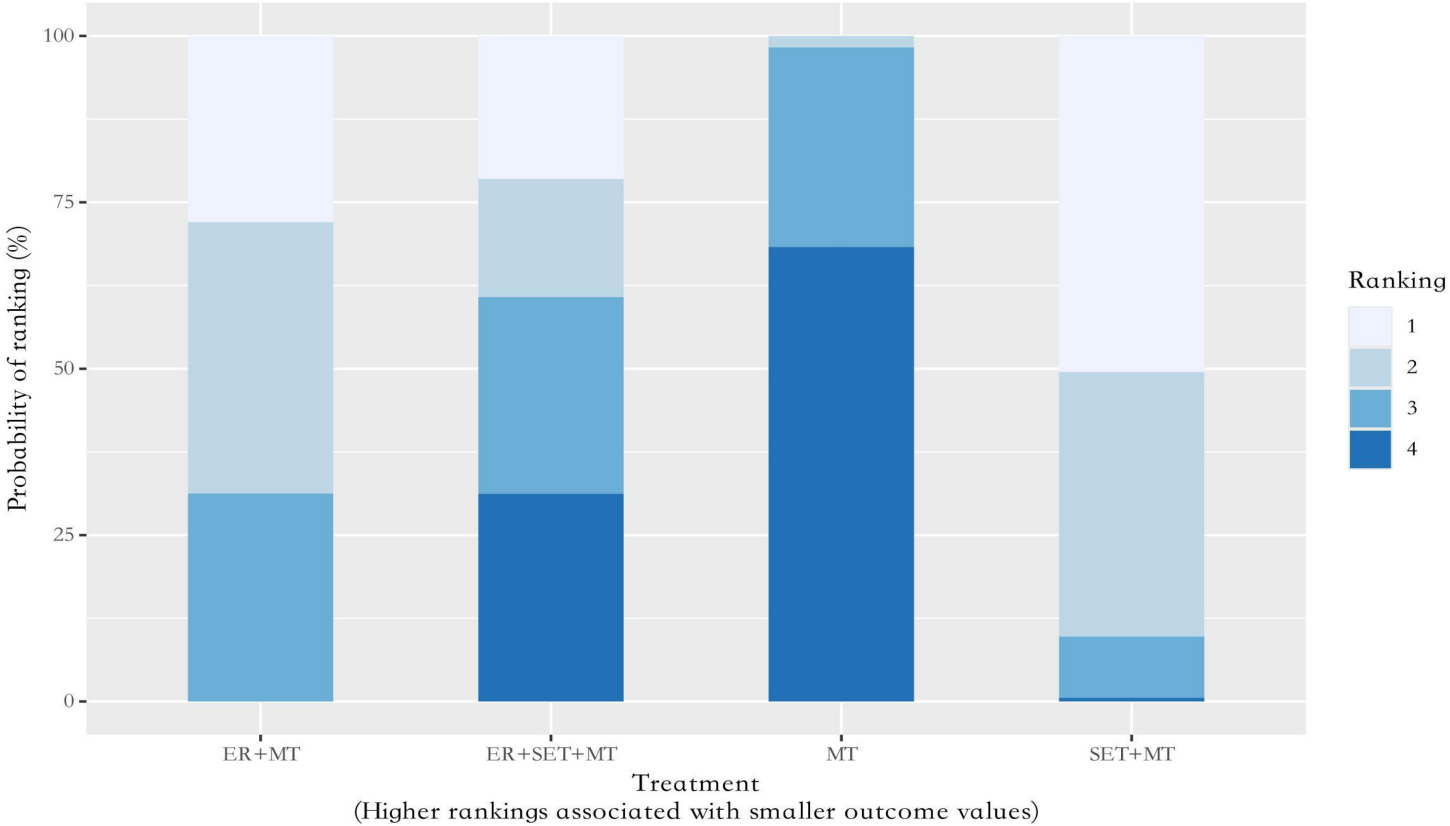
Ranking probability distribution for amputation incidence. Lighter colors represent higher treatment rankings and lower amputation risk.

Heterogeneity was minimal (I2 = 0.0%, *P* = 0.785), and node-splitting analysis indicated no significant inconsistency (*P* > 0.05). A consistency fixed-effect model was used. SUCRA rankings for reducing amputation risk were SET + MT (80.08%) > ER + MT (65.58%) > ER + SET + MT (43.19%) > MT (11.15%). SET + MT therefore ranked most favorably for limb-salvage probability, with a mean rank of 1.7. Given the small number of direct comparisons involving SET-based strategies and the possibility of differences in baseline limb threat, this ranking should be interpreted cautiously. Pairwise estimates are shown in Table 5, and ranking results are shown in Figures 20, 21.

**TABLE 5:** League table for amputation incidence.

ER_MT	ER_SET_MT	MT	SET_MT
ER_MT	1.885 (0.070, 94.009)	4.035 (2.293, 7.618)	0.699 (0.106, 3.823)
0.531 (0.011, 14.294)	ER_SET_MT	2.150 (0.040, 60.170)	0.376 (0.010, 5.552)
0.248 (0.131, 0.436)	0.465 (0.017, 24.981)	MT	0.173 (0.023, 1.021)
1.431 (0.262, 9.470)	2.658 (0.180, 95.410)	5.768 (0.979, 42.934)	SET_MT

Values are odds ratios with 95% confidence intervals.

**FIGURE 22:**
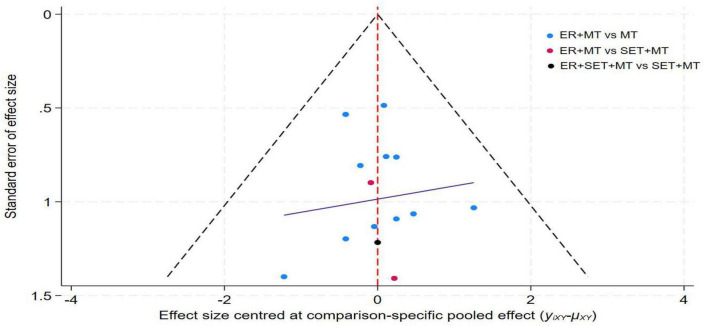
Comparison-adjusted funnel plot for amputation incidence. The generally symmetrical distribution and minimal regression-line tilt suggest a low likelihood of substantial publication bias.

The comparison-adjusted funnel plot showed a largely symmetrical distribution of points within the 95% confidence region and minimal tilt of the regression line, suggesting a lower risk of substantial publication bias for this outcome (Figure 22). Sensitivity analyses supported the robustness of the overall findings. Subgroup analysis by medication intensity, after excluding one potential outlier (20), showed protective effects in both the BMT subgroup (I2 = 0.0%, *P* = 0.65; OR = 0.44, 95% CI: 0.29–0.67, *P* = 0.0001) and the OMT subgroup (I2 = 62%, *P* = 0.01; OR = 0.25, 95% CI: 0.18–0.35, *P* < 0.00001). A statistically significant subgroup difference was observed (*P* = 0.04), suggesting that the magnitude of amputation protection appeared larger for ER + OMT than for ER + BMT. This does not necessarily indicate a different direction of treatment effect; rather, BMT may already provide substantial baseline protection, thereby reducing the incremental benefit of ER. Subgroup analysis by follow-up duration showed consistent effects for < = 6 months (I2 = 33%, *P* = 0.21; OR = 0.31, 95% CI: 0.23–0.42, *P* < 0.00001) and > 6 months (I2 = 87%, *P* < 0.00001; OR = 0.33, 95% CI: 0.26–0.42, *P* < 0.00001), with no significant subgroup difference (I2 = 0%, *P* = 0.72).

For this outcome, the statistical difference in magnitude between the OMT and BMT subgroups should be interpreted carefully. The direction of effect remained protective across subgroups, and both OMT and BMT represent pharmacologic foundations for PAD care. However, BMT may provide stronger baseline vascular protection than OMT, which could reduce the apparent incremental benefit of adding ER. Therefore, the combined MT node remains clinically interpretable for network construction, but medication intensity should be considered when applying the findings to practice and when interpreting ER-based rankings.

#### Ankle-brachial index

**FIGURE 23:**
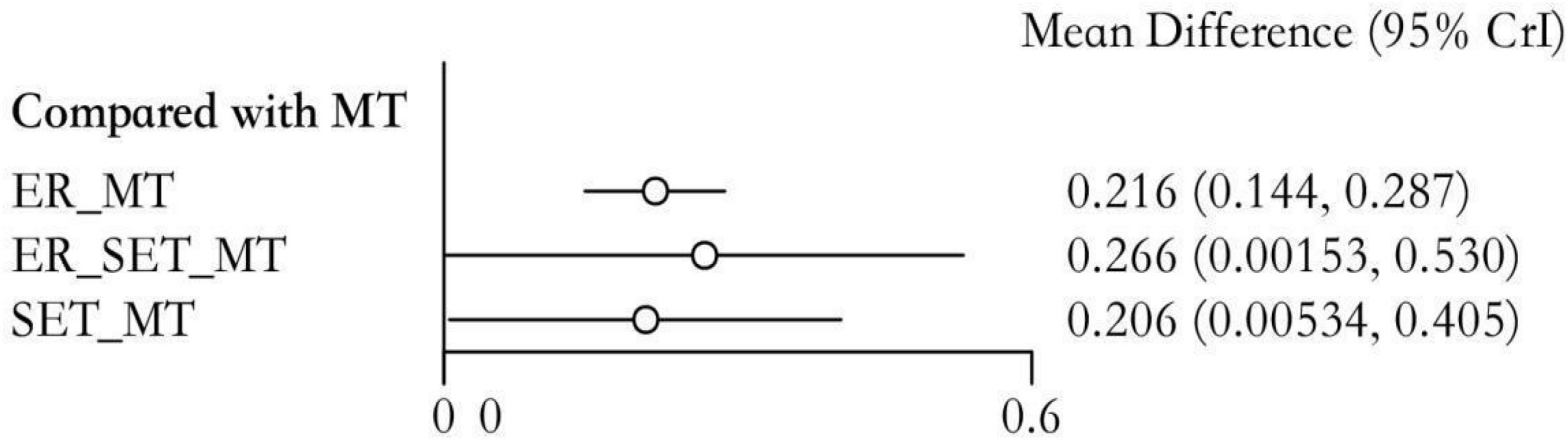
Forest plot of network meta-analysis for ankle-brachial index (ABI) improvement, with MT as the common control. MD > 0 indicates greater improvement in ABI in the intervention group. ER + MT, endovascular revascularization plus medication; ER + SET + MT, endovascular revascularization plus supervised exercise plus medication; SET + MT, supervised exercise plus medication; MT, medication.

**FIGURE 24:**
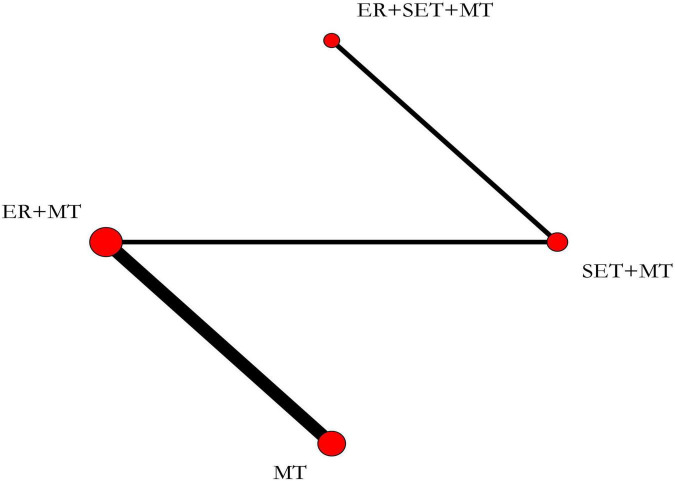
Network plot for ankle-brachial index. Node size is proportional to sample size, and line thickness is proportional to the number of studies.

Nine studies (19, 21, 23, 24, 26, 28, 29, 31, 32) reported ABI as a continuous outcome. The network included MT, ER + MT, SET + MT, and ER + SET + MT. The forest plot is shown in Figure 23. The network plot (Figure 24) was connected, with ER + MT versus MT as the principal comparison.

**FIGURE 25:**
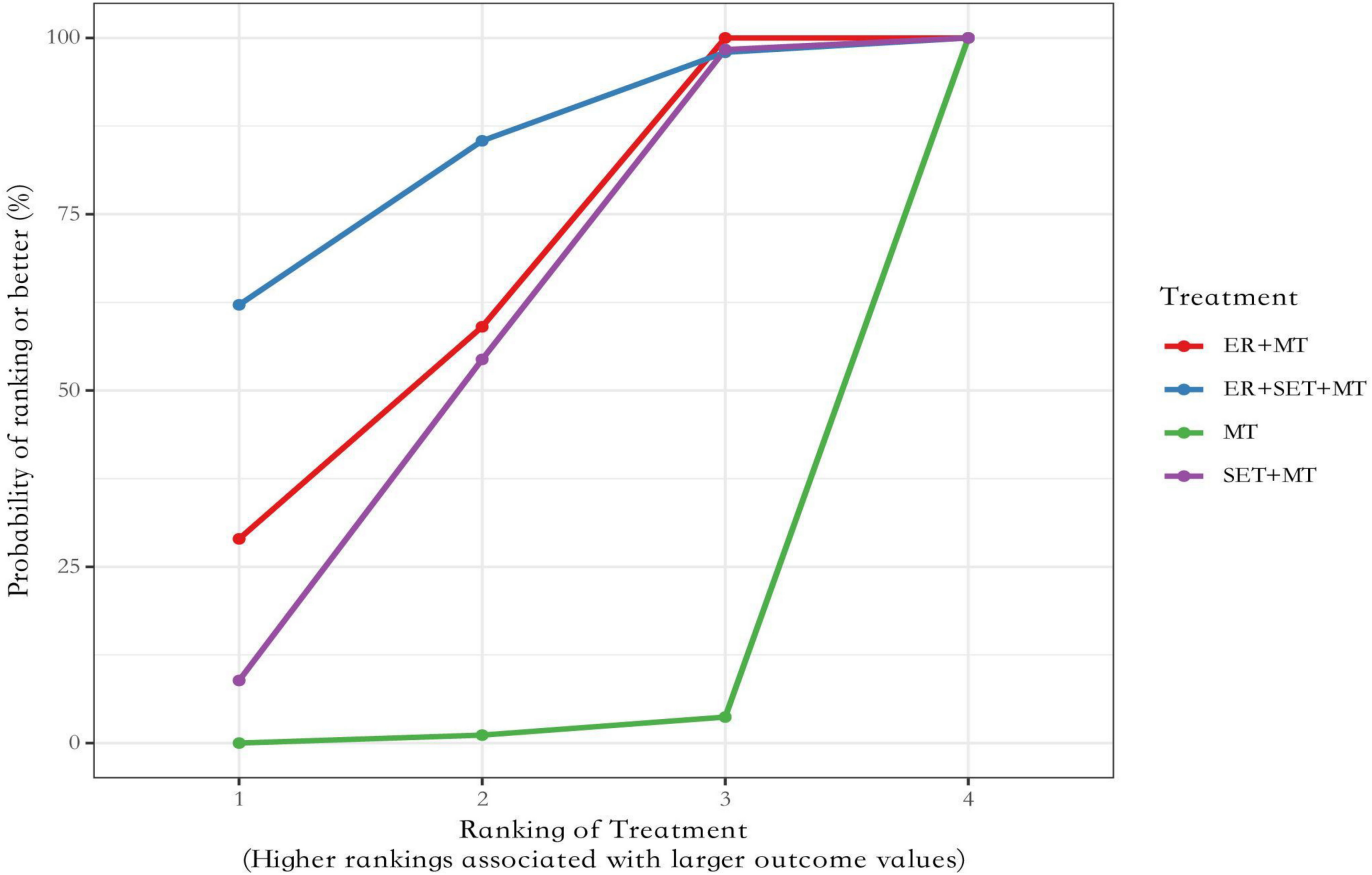
Ranking probability plot for ankle-brachial index improvement. Higher rankings are associated with greater ABI improvement.

**FIGURE 26:**
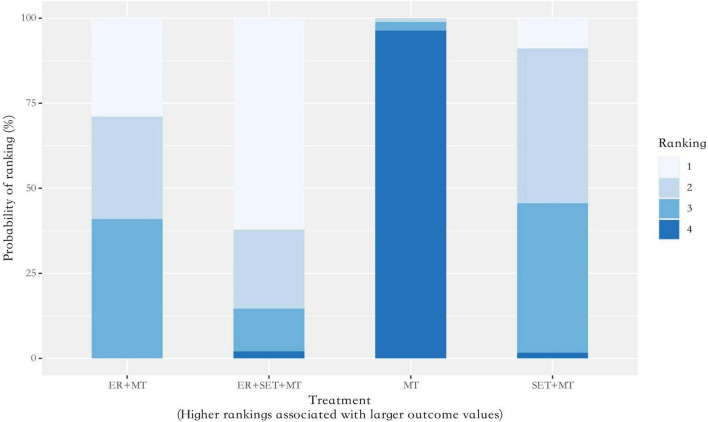
Ranking probability distribution for ankle-brachial index improvement. Lighter colors represent higher treatment rankings and greater ABI improvement.

Substantial heterogeneity was observed (I2 = 91.1%, *P* = 0.000). Node-splitting analysis did not detect statistically significant inconsistency (*P* > 0.05), and a consistency random-effects model was therefore used. SUCRA rankings for ABI improvement were ER + SET + MT (81.84%) > ER + MT (62.67%) > SET + MT (53.88%) > MT (1.61%). ER + SET + MT ranked most favorably for ABI improvement, with a mean rank of 1.4. Because ABI change is strongly influenced by lesion anatomy, revascularization method, baseline perfusion deficit, and follow-up duration, the magnitude of this benefit should be considered heterogeneous rather than uniform across PAD populations. Pairwise estimates are shown in Table 6, and ranking results are shown in Figures 25, 26.

**TABLE 6:** League table for ankle-brachial index improvement.

ER_MT	ER_SET_MT	MT	SET_MT
ER_MT	0.050 (−0.202, 0.303)	−0.216 (−0.287, −0.144)	−0.010 (−0.197, 0.176)
−0.050 (−0.303, 0.202)	ER_SET_MT	−0.265 (−0.527, −0.004)	−0.060 (−0.231, 0.111)
0.216 (0.144, 0.287)	0.265 (0.004, 0.527)	MT	0.206 (0.005, 0.404)
0.010 (−0.176, 0.197)	0.060 (−0.111, 0.231)	−0.206 (−0.404, −0.005)	SET_MT

Values are mean differences with 95% confidence intervals.

**FIGURE 27:**
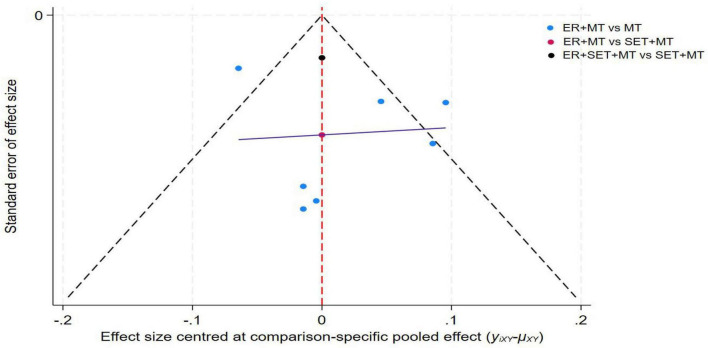
Comparison-adjusted funnel plot for ankle-brachial index. The generally symmetrical point distribution and nearly horizontal regression line suggest a low likelihood of substantial publication bias.

The comparison-adjusted funnel plot displayed a generally symmetrical point distribution and a nearly horizontal regression line, indicating a low likelihood of substantial publication bias (Figure 27). Sensitivity analyses excluding studies with a SET + MT comparator and removing one study identified as a possible source of heterogeneity (28) markedly reduced heterogeneity (from I2 = 93% to 7%, *P* = 0.37), and the recalculated effect estimate remained favorable (MD = 0.18, 95% CI: 0.15–0.21, *P* < 0.00001). Repeated network meta-analysis produced rankings consistent with the main analysis. A subgroup analysis limited to OMT studies after excluding one BMT study showed I2 = 0% and a sustained improvement with ER + OMT (MD = 0.20, *P* < 0.00001). Subgroup analysis by follow-up duration showed significant ABI improvement with ER + MT at = 6 months (I2 = 0%, *P* = 0.44; MD = 0.21, 95% CI: 0.15–0.26, *P* < 0.00001) and > 6 months (I2 = 24%, *P* = 0.25; MD = 0.17, 95% CI: 0.12–0.21, *P* < 0.00001), with no significant subgroup interaction (I2 = 7%, *P* = 0.24).

## Discussion

This systematic review and network meta-analysis synthesized evidence from 15 RCTs involving 1,461 patients with PAD. The analysis compared four clinically relevant strategies-MT, ER + MT, SET + MT, and ER + SET + MT-across long-term systemic and limb-specific outcomes. Overall, combined strategies generally ranked above MT alone, but the most favorable regimen differed by outcome. ER + SET + MT ranked highest for all-cause mortality and ABI improvement, ER + MT ranked highest for cardiovascular events and cerebral infarction, and SET + MT ranked highest for amputation prevention. These findings should be read as ranking patterns within a sparse and clinically heterogeneous evidence network rather than as definitive proof that any one strategy is universally superior.

For all-cause mortality, ER + SET + MT had the highest SUCRA probability. This finding is clinically plausible because the triple strategy combines rapid hemodynamic improvement, structured functional rehabilitation, and pharmacologic risk-factor control. However, the direct evidence supporting ER + SET + MT was limited, and SUCRA values should not be interpreted as proof of statistically significant superiority. Rather, they indicate the probability of a treatment occupying a favorable rank within the available evidence network. The conclusion that ER + SET + MT may reduce mortality therefore remains hypothesis-generating and should be confirmed by larger head-to-head trials.

ER + MT ranked highest for cardiovascular events and cerebral infarction. This pattern suggests that revascularization combined with pharmacotherapy may provide systemic vascular benefit in addition to local limb perfusion improvement. The effect was directionally consistent across medication-intensity and follow-up subgroups. Nevertheless, cardiovascular and cerebrovascular events are influenced by background risk-factor control, adherence to antiplatelet and lipid-lowering therapy, diabetes control, smoking status, and baseline atherosclerotic burden. These factors were not uniformly reported across trials and may contribute to residual heterogeneity.

The amputation results require careful interpretation. SET + MT ranked highest, followed by ER + MT and ER + SET + MT, while MT alone ranked lowest. This does not mean that ER lacks limb-salvage value. Rather, the included studies differed in disease severity, lesion anatomy, background medication intensity, and indications for revascularization. Patients selected for ER may have more severe limb ischemia or anatomically significant lesions. The subgroup finding that ER + OMT showed a larger magnitude of amputation protection than ER + BMT suggests that optimized medical therapy itself may reduce the marginal benefit of revascularization for limb outcomes. Clinically, SET remains essential for functional improvement and limb preservation, but ER may still be needed for patients with severe ischemia, unacceptable symptoms, or threatened limbs.

For ABI, ER + SET + MT ranked highest. This result is consistent with the mechanistic expectation that endovascular treatment improves macroscopic perfusion, while SET improves walking efficiency, skeletal muscle metabolism, endothelial function, and collateral circulation. The high heterogeneity for ABI indicates that the magnitude of improvement varied across studies, likely because of differences in lesion site, baseline ABI, ER technique, follow-up duration, and exercise protocol. Therefore, the ABI findings support a combined-treatment concept but should be applied with attention to patient selection and treatment feasibility.

The present findings align with previous research while extending it to a broader set of long-term outcomes. Previous network meta-analyses and pairwise meta-analyses reported that ER + SET can improve walking distance and quality of life in intermittent claudication (34, 35). Pandey et al. (36) found that ER + SET improved maximal walking distance compared with SET alone and may reduce amputation risk, while ER alone did not consistently improve functional capacity. Other evidence indicates that SET is among the few interventions that reliably improves daily physical activity and walking performance, whereas home-based exercise or walking advice alone may be less effective (37, 38). Shirasu et al. (39) reported that long-term risks of amputation and death may not differ substantially between invasive revascularization and non-invasive management in intermittent claudication, although revascularization may increase reintervention risk. Weissler et al. (40) showed that PAD patients remain at high risk of cardiovascular and limb events after ER, and that guideline-recommended BMT remains underused. The 2024 ESC guideline, the 2024 ACC/AHA multisociety guideline, and the 2024 ESVS guideline all emphasize comprehensive risk-factor management, antithrombotic and lipid-lowering therapy, structured exercise for symptomatic PAD or intermittent claudication, and selective revascularization for patients with chronic limb-threatening ischemia or persistent functionally limiting symptoms despite guideline-directed care (3, 9, 41). Together, these data support the view that PAD management should not rely on one modality alone but should integrate revascularization, structured exercise, and pharmacologic prevention according to patient phenotype, lesion anatomy, limb threat, functional limitation, and cardiovascular risk.

This analysis has several strengths. It focused on RCTs, used PRISMA-NMA reporting principles, assessed risk of bias using RoB 2.0, explored inconsistency with node-splitting, conducted sensitivity and subgroup analyses, and evaluated confidence in the evidence using CINeMA. These features improve transparency and allow readers to assess the reliability of each outcome-specific ranking.

Additional caution is warranted for treatment rankings. SUCRA values are useful for summarizing relative ranking probabilities, but they do not account fully for clinical heterogeneity, absolute effect size, quality of direct evidence, or patient-level applicability. For this reason, rankings in this manuscript are presented as supportive signals and are discussed together with confidence intervals, directness of evidence, consistency tests, sensitivity analyses, and CINeMA certainty.

Several limitations should also be acknowledged. First, the number of included studies and the sample size for some comparisons were modest, especially for ER + SET + MT. Sparse direct evidence may reduce the precision of network estimates and make treatment rankings unstable. Second, clinical heterogeneity was unavoidable. Trials differed in PAD phenotype, disease severity, lesion location, baseline ABI, ER technique, medication regimen, SET intensity, adherence, and follow-up duration. These differences may affect the transitivity assumption even when node-splitting tests do not detect statistical inconsistency. Third, OMT and BMT were merged into a single MT node to maintain network stability. This approach was necessary because the available data did not support a fully separated network, but it may mask differences in pharmacologic intensity and may influence indirect comparisons and ER-based rankings. Fourth, some studies did not report randomization, allocation concealment, blinding, adherence, and missing-data handling in sufficient detail, which may introduce bias. Fifth, comparison-adjusted funnel plots suggested possible publication bias or small-study effects for several outcomes, particularly mortality, cardiovascular events, and cerebral infarction. Sixth, outcome definitions such as cardiovascular events, cerebral infarction, and amputation were not fully standardized across trials, and harmonization relied on the definitions reported in the original studies. Seventh, this analysis did not stratify by specific ER method, lesion anatomy, chronic limb-threatening ischemia versus intermittent claudication, or exercise protocol. Finally, aggregate study-level data do not allow individualized treatment-effect estimation according to patient-level risk factors.

Clinically, the findings suggest that treatment selection should be outcome oriented and guideline consistent. ER + MT may be reasonable when revascularization is indicated and cardiovascular or cerebrovascular risk reduction is a key priority, SET + MT should remain central for intermittent claudication and functional improvement, and ER + SET + MT may be considered for selected patients who have persistent symptoms, significant hemodynamic impairment, or higher limb risk and who can tolerate integrated care. Because certainty was mainly moderate to low, these results should be viewed as supportive comparative evidence rather than definitive clinical rules. Decisions should still be individualized according to PAD phenotype, lesion anatomy, limb threat, comorbidity burden, patient preference, and local access to supervised exercise and endovascular expertise.

## Conclusion

Among patients with PAD, combined treatment strategies generally ranked above MT alone. ER + SET + MT appeared to rank favorably for all-cause mortality and ABI improvement, ER + MT ranked favorably for cardiovascular and cerebrovascular outcomes, and SET + MT ranked favorably for amputation prevention. These findings support integrated PAD management built around pharmacologic risk-factor control, structured exercise, and selective revascularization. However, SUCRA rankings should be interpreted as probability-based summaries rather than definitive evidence of superiority, and sparse direct evidence, clinical heterogeneity, merged OMT/BMT definitions, variable endpoint definitions, and possible publication bias limit certainty. Future large, long-term, head-to-head RCTs should clarify which PAD phenotypes benefit most from each combined strategy and whether these benefits are sustained across disease severity, lesion anatomy, and treatment settings.

## Data Availability

Publicly available datasets were analyzed in this study. This data can be found at: All data used in this study are derived from published randomized controlled trials, which are publicly available in peer-reviewed journals. No new datasets or accession numbers are associated with this analysis.
