# Supplementary material for: Impact of endovascular revascularization, pharmacotherapy, and supervised exercise therapy on long-term cardiovascular, cerebrovascular, mortality, and limb outcomes in patients with peripheral artery disease: a systematic review and network meta-analysis
Source: Front Med (Lausanne). 2026 Jul 1;13:1874951. doi: 10.3389/fmed.2026.1874951 (PMC13370290; doi:10.3389/fmed.2026.1874951)
Supplement: Supplementary file 1 [file Data_Sheet_1.PDF]

Search Strategies for Systematic Review and Meta-Analysis

Title: Endovascular Therapy Versus Best Medical Therapy, Supervised Exercise Therapy, or Conservative Treatment for Patients With Lower Extremity Arteriosclerosis Obliterans and Intermittent Claudication: A Meta-Analysis of Long-Term Major Adverse Limb Events, Amputation, and New Ulceration

Databases searched: PubMed, Web of Science, Scopus, Ovid, Embase, Cochrane Library, CBM, Wanfang, VIP, CNKI.(**Additional relevant databases were also searched but yielded no eligible records.**)

Search date: Up to April 8, 2026

Study type: Randomized controlled trials (RCTs)

1. PubMed

Population (Disease)

((peripheral arterial disease[MeSH Terms]) OR (arteriosclerosis obliterans[MeSH Terms]) OR (Intermittent Claudication[MeSH Terms])) OR (((((((((((((((((Arterial Disease, Peripheral[Title/Abstract]) OR (Arterial Diseases, Peripheral[Title/Abstract]) OR (Disease, Peripheral Arterial[Title/Abstract]) OR (Diseases, Peripheral Arterial[Title/Abstract]) OR (Peripheral Arterial Diseases[Title/Abstract]) OR (Peripheral Artery Disease[Title/Abstract]) OR (Artery Disease, Peripheral[Title/Abstract]) OR (Artery Diseases, Peripheral[Title/Abstract]) OR (Disease, Peripheral Artery[Title/Abstract]) OR (Diseases, Peripheral Artery[Title/Abstract]) OR (Peripheral Artery Diseases[Title/Abstract]) OR (lower extremity arterial occlusive disease[Title/Abstract]) OR (peripheral arterial occlusive disease[Title/Abstract]) OR (PAD[Title/Abstract]) OR (ASO[Title/Abstract]) OR (Obliterans, Arteriosclerosis[Title/Abstract]) OR (Claudication, Intermittent[Title/Abstract])))

Intervention

(Angioplasty[MeSH Terms]) OR ((((((((((((((((((Angioplasties[Title/Abstract]) OR (Endoluminal Repair[Title/Abstract]) OR (Endoluminal Repairs[Title/Abstract]) OR (Repair, Endoluminal[Title/Abstract]) OR (Repairs, Endoluminal[Title/Abstract]) OR (Angioplasty, Transluminal[Title/Abstract]) OR (Transluminal Angioplasty[Title/Abstract]) OR (Percutaneous Transluminal Angioplasty[Title/Abstract]) OR (Angioplasty, Percutaneous Transluminal[Title/Abstract]) OR (Transluminal Angioplasty, Percutaneous[Title/Abstract]) OR (Endovascular intervention[Title/Abstract]) OR (PTA[Title/Abstract]) OR (Stenting[Title/Abstract]) OR (Peripheral artery stenting[Title/Abstract]) OR (Lower extremity endovascular treatment[Title/Abstract]) OR (Lower limb angioplasty[Title/Abstract]) OR (Percutaneous transluminal revascularization[Title/Abstract]) OR (PTR[Title/Abstract])))

Study Type

randomized controlled trial[Publication Type] OR randomized[Title/Abstract] OR placebo[Title/Abstract]

Final Search

(Population) AND (Intervention) AND (Study Type)

2. Web of Science

#1 TS=(peripheral arterial disease OR arteriosclerosis obliterans OR Intermittent Claudication OR Arterial Disease, Peripheral OR Arterial Diseases, Peripheral OR Disease, Peripheral Arterial OR Diseases, Peripheral Arterial OR Peripheral Arterial Diseases OR Peripheral Artery Disease OR

Artery Disease, Peripheral OR Artery Diseases, Peripheral OR Disease, Peripheral Artery OR Diseases, Peripheral Artery OR Peripheral Artery Diseases OR lower extremity arterial occlusive disease OR peripheral arterial occlusive disease OR PAD OR ASO OR Obliterans, Arteriosclerosis OR Claudication, Intermittent)

#2 TS=(Angioplasty OR Angioplasties OR Endoluminal Repair OR Endoluminal Repairs OR Repair, Endoluminal OR Repairs, Endoluminal OR Angioplasty, Transluminal OR Transluminal Angioplasty OR Percutaneous Transluminal Angioplasty OR Angioplasty, Percutaneous Transluminal OR Transluminal Angioplasty, Percutaneous OR Endovascular intervention OR PTA OR Stenting OR Peripheral artery stenting OR Lower extremity endovascular treatment OR Lower limb angioplasty OR Percutaneous transluminal revascularization OR PTR)

#3 TS=(randomized controlled trial OR randomized OR placebo OR random OR randomised)

#4 #1 AND #2 AND #3

3. Scopus

("peripheral arterial disease" OR "arteriosclerosis obliterans" OR "Intermittent Claudication" OR "Arterial Disease, Peripheral" OR "Arterial Diseases, Peripheral" OR "Disease, Peripheral Arterial" OR "Diseases, Peripheral Arterial" OR "Peripheral Arterial Diseases" OR "Peripheral Artery Disease" OR "Artery Disease, Peripheral" OR "Artery Diseases, Peripheral" OR "Disease, Peripheral Artery" OR "Diseases, Peripheral Artery" OR "Peripheral Artery Diseases" OR "lower extremity arterial occlusive disease" OR "peripheral arterial occlusive disease" OR "PAD" OR "ASO" OR "Obliterans, Arteriosclerosis" OR "Claudication, Intermittent")

AND

("Angioplasty" OR "Angioplasties" OR "Endoluminal Repair" OR "Endoluminal Repairs" OR "Repair, Endoluminal" OR "Repairs, Endoluminal" OR "Angioplasty, Transluminal" OR "Transluminal Angioplasty" OR "Percutaneous Transluminal Angioplasty" OR "Angioplasty, Percutaneous Transluminal" OR "Transluminal Angioplasty, Percutaneous" OR "Endovascular intervention" OR "PTA" OR "Stenting" OR "Peripheral artery stenting" OR "Lower extremity endovascular treatment" OR "Lower limb angioplasty" OR "Percutaneous transluminal revascularization" OR "PTR")

AND

("randomized controlled trial" OR "randomized" OR "placebo")

4. Ovid

#1 (peripheral arterial disease or arteriosclerosis obliterans or Intermittent Claudication or Arterial Disease, Peripheral or Arterial Diseases, Peripheral or Disease, Peripheral Arterial or Diseases, Peripheral Arterial or Peripheral Arterial Diseases or Peripheral Artery Disease or Artery Disease, Peripheral or Artery Diseases, Peripheral or Disease, Peripheral Artery or Diseases, Peripheral Artery or Peripheral Artery Diseases or lower extremity arterial occlusive disease or peripheral arterial occlusive disease or PAD or ASO or Obliterans, Arteriosclerosis or Claudication, Intermittent).ti,ab,kw.

#2 (Angioplasty or Angioplasties or Endoluminal Repair or Endoluminal Repairs or Repair, Endoluminal or Repairs, Endoluminal or Angioplasty, Transluminal or Transluminal Angioplasty or Percutaneous Transluminal Angioplasty or Angioplasty, Percutaneous Transluminal or Transluminal Angioplasty, Percutaneous or Endovascular intervention or PTA or Stenting or Peripheral artery stenting or Lower extremity endovascular treatment or Lower limb angioplasty or Percutaneous transluminal revascularization or PTR).ti,ab,kw.

#3 (randomized controlled trial or randomized or placebo).ti,ab,kw.
#4 #1 AND #2 AND #3

5. Embase

#1 peripheral AND arterial AND ('disease'/exp OR disease)
#2 ('arteriosclerosis'/exp OR arteriosclerosis) AND obliterans
#3 intermittent AND ('claudication'/exp OR claudication)
#4 #1 OR #2 OR #3
#5 'arterial disease, peripheral':ab,ti OR 'arterial diseases, peripheral':ab,ti OR 'disease, peripheral arterial':ab,ti OR 'diseases, peripheral arterial':ab,ti OR 'peripheral arterial diseases':ab,ti OR 'peripheral artery disease':ab,ti OR 'artery disease, peripheral':ab,ti OR 'artery diseases, peripheral':ab,ti OR 'disease, peripheral artery':ab,ti OR 'diseases, peripheral artery':ab,ti OR 'peripheral artery diseases':ab,ti OR 'lower extremity arterial occlusive disease':ab,ti OR 'peripheral arterial occlusive disease':ab,ti OR 'pad':ab,ti OR 'aso':ab,ti OR 'obliterans, arteriosclerosis':ab,ti OR 'claudication, intermittent':ab,ti
#6 #4 OR #5
#7 angioplasty
#8 'angioplasties':ab,ti OR 'endoluminal repair':ab,ti OR 'endoluminal repairs':ab,ti OR 'repair, endoluminal':ab,ti OR 'repairs, endoluminal':ab,ti OR 'angioplasty, transluminal':ab,ti OR 'transluminal angioplasty':ab,ti OR 'percutaneous transluminal angioplasty':ab,ti OR 'angioplasty, percutaneous transluminal':ab,ti OR 'transluminal angioplasty, percutaneous':ab,ti OR 'endovascular intervention':ab,ti OR 'pta':ab,ti OR 'stenting':ab,ti OR 'peripheral artery stenting':ab,ti OR 'lower extremity endovascular treatment':ab,ti OR 'lower limb angioplasty':ab,ti OR 'percutaneous transluminal revascularization':ab,ti OR 'ptr':ab,ti
#9 #7 OR #8
#10 'randomized controlled trial':ab,ti OR 'randomized':ab,ti OR 'placebo':ab,ti
#11 #6 AND #9 AND #10

6. Cochrane Library

#1 MeSH descriptor: [Arteriosclerosis Obliterans] explode all trees
#2 MeSH descriptor: [Intermittent Claudication] explode all trees
#3 MeSH descriptor: [Peripheral Arterial Disease] explode all trees
#4 (Arterial Disease, Peripheral):ti,ab,kw OR (Arterial Diseases, Peripheral):ti,ab,kw OR (Disease, Peripheral Arterial):ti,ab,kw OR (Diseases, Peripheral Arterial):ti,ab,kw OR (Peripheral Arterial Diseases):ti,ab,kw OR (Peripheral Artery Disease):ti,ab,kw OR (Artery Disease, Peripheral):ti,ab,kw OR (Artery Diseases, Peripheral):ti,ab,kw OR (Disease, Peripheral Artery):ti,ab,kw OR (Diseases, Peripheral Artery):ti,ab,kw OR (Peripheral Artery Diseases):ti,ab,kw OR (lower extremity arterial occlusive disease):ti,ab,kw OR (peripheral arterial occlusive disease):ti,ab,kw OR (PAD):ti,ab,kw OR (ASO):ti,ab,kw OR (Obliterans, Arteriosclerosis):ti,ab,kw OR (Claudication, Intermittent):ti,ab,kw
#5 #1 OR #2 OR #3 OR #4
#6 MeSH descriptor: [Angioplasty] explode all trees
#7 (Angioplasties):ti,ab,kw OR (Endoluminal Repair):ti,ab,kw OR (Endoluminal Repairs):ti,ab,kw OR (Repair, Endoluminal):ti,ab,kw OR (Repairs, Endoluminal):ti,ab,kw OR (Angioplasty, Transluminal):ti,ab,kw OR (Transluminal Angioplasty):ti,ab,kw OR (Percutaneous Transluminal Angioplasty):ti,ab,kw OR (Angioplasty, Percutaneous Transluminal):ti,ab,kw OR

(Transluminal Angioplasty, Percutaneous):ti,ab,kw OR (Endovascular intervention):ti,ab,kw OR (PTA):ti,ab,kw OR (Stenting):ti,ab,kw OR (Peripheral artery stenting):ti,ab,kw OR (Lower extremity endovascular treatment):ti,ab,kw OR (Lower limb angioplasty):ti,ab,kw OR (Percutaneous transluminal revascularization):ti,ab,kw OR (PTR):ti,ab,kw

#8 #6 OR #7

#9 #5 AND #8

7. Chinese Databases (Brief English Summary for Supplementary Material)

Searched in CBM, Wanfang, VIP, CNKI

Population: lower extremity arteriosclerosis obliterans, intermittent claudication, lower extremity arterial occlusion, chronic lower limb ischemia

Intervention: endovascular therapy, angioplasty, stent, PTA, endovascular intervention

Study type: randomized controlled trial (random, RCT)
