# Supplementary figures and images for: Impact of endovascular revascularization, pharmacotherapy, and supervised exercise therapy on long-term cardiovascular, cerebrovascular, mortality, and limb outcomes in patients with peripheral artery disease: a systematic review and network meta-analysis
Source: Front Med (Lausanne). 2026 Jul 1;13:1874951. doi: 10.3389/fmed.2026.1874951 (PMC13370290; doi:10.3389/fmed.2026.1874951)

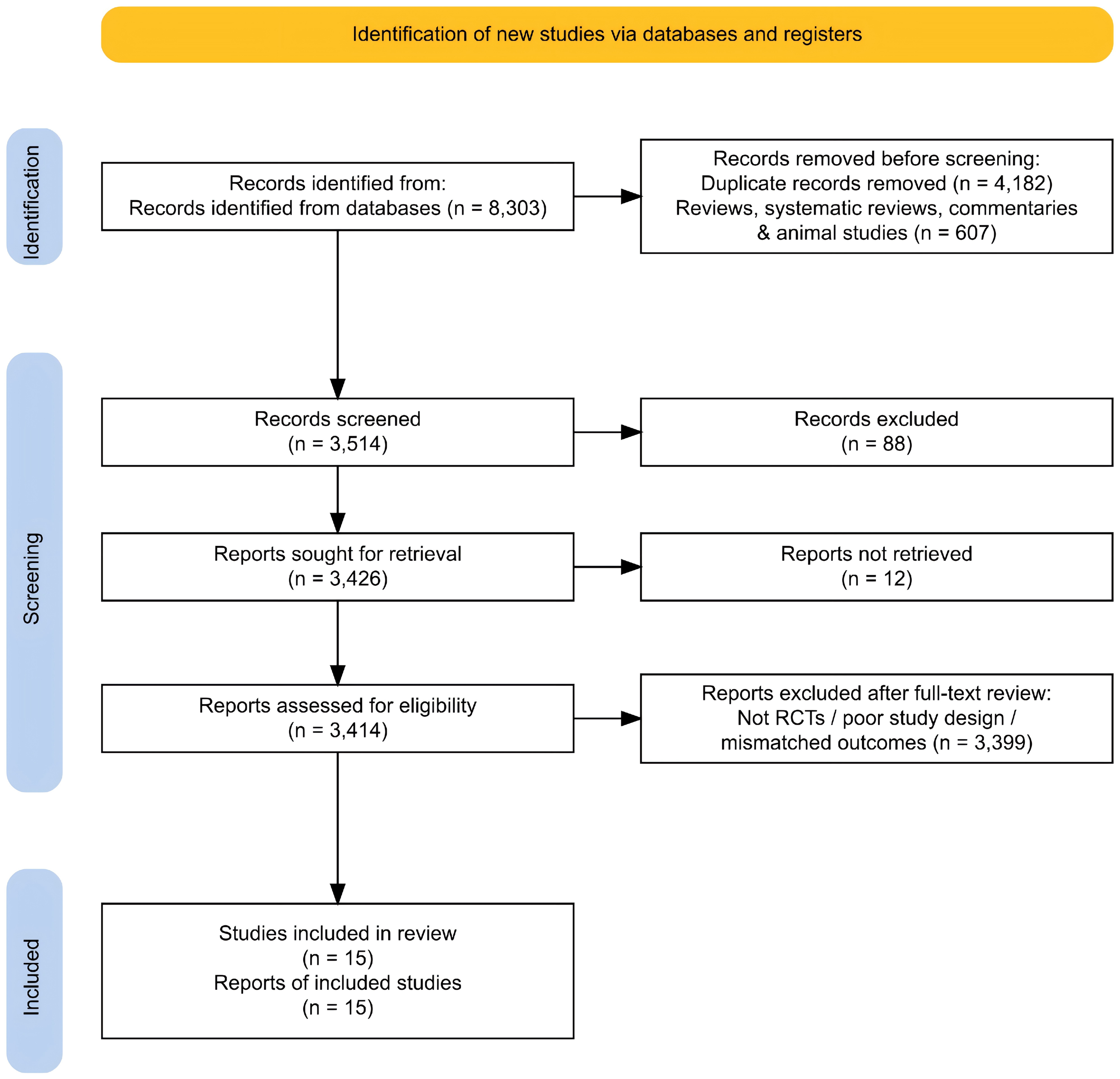

Supplement: Supplementary file 3 [file Data_Sheet_3.ZIP › Figure/Figure 1.tif]

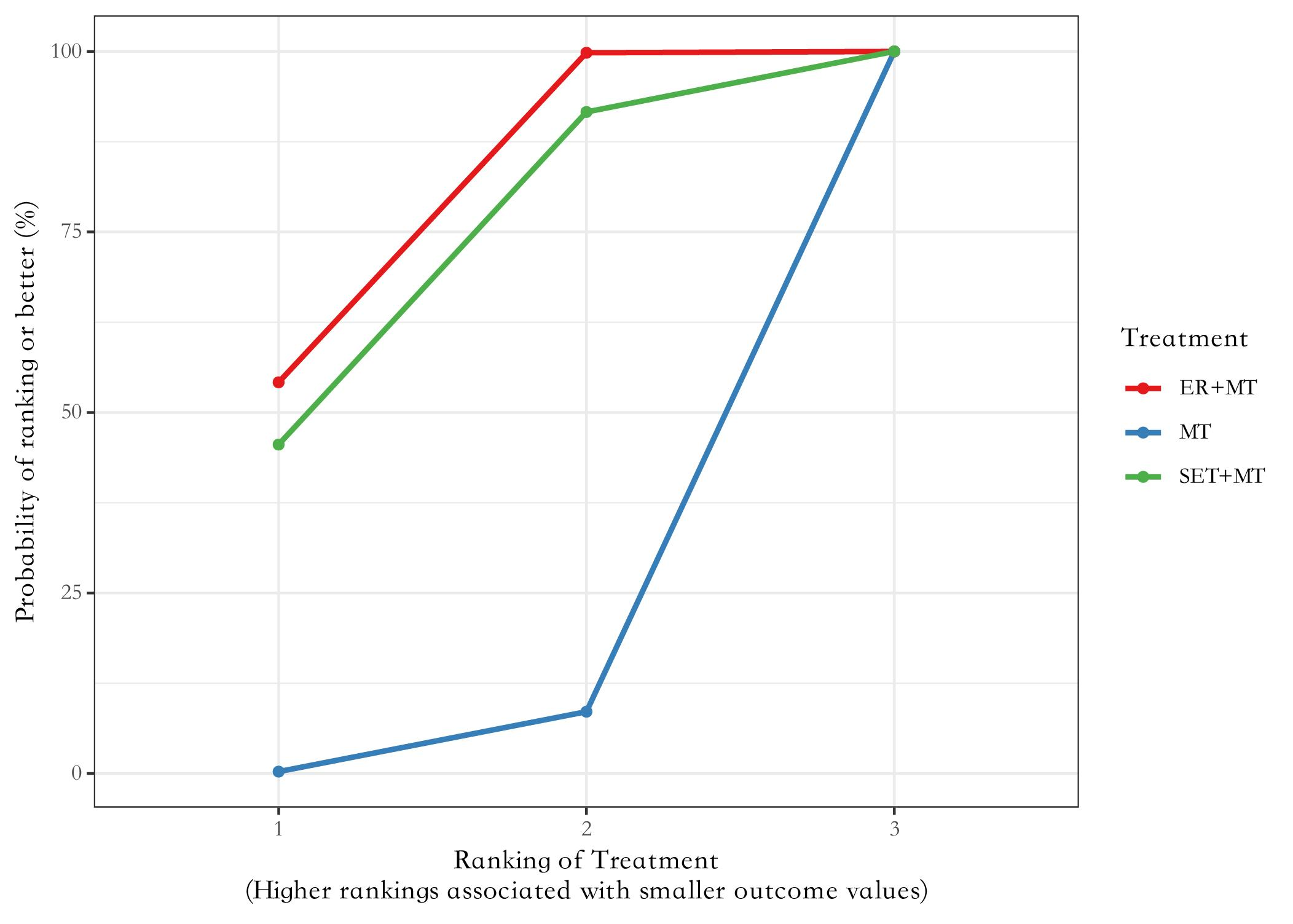

Supplement: Supplementary file 3 [file Data_Sheet_3.ZIP › Figure/Figure 10.tif]

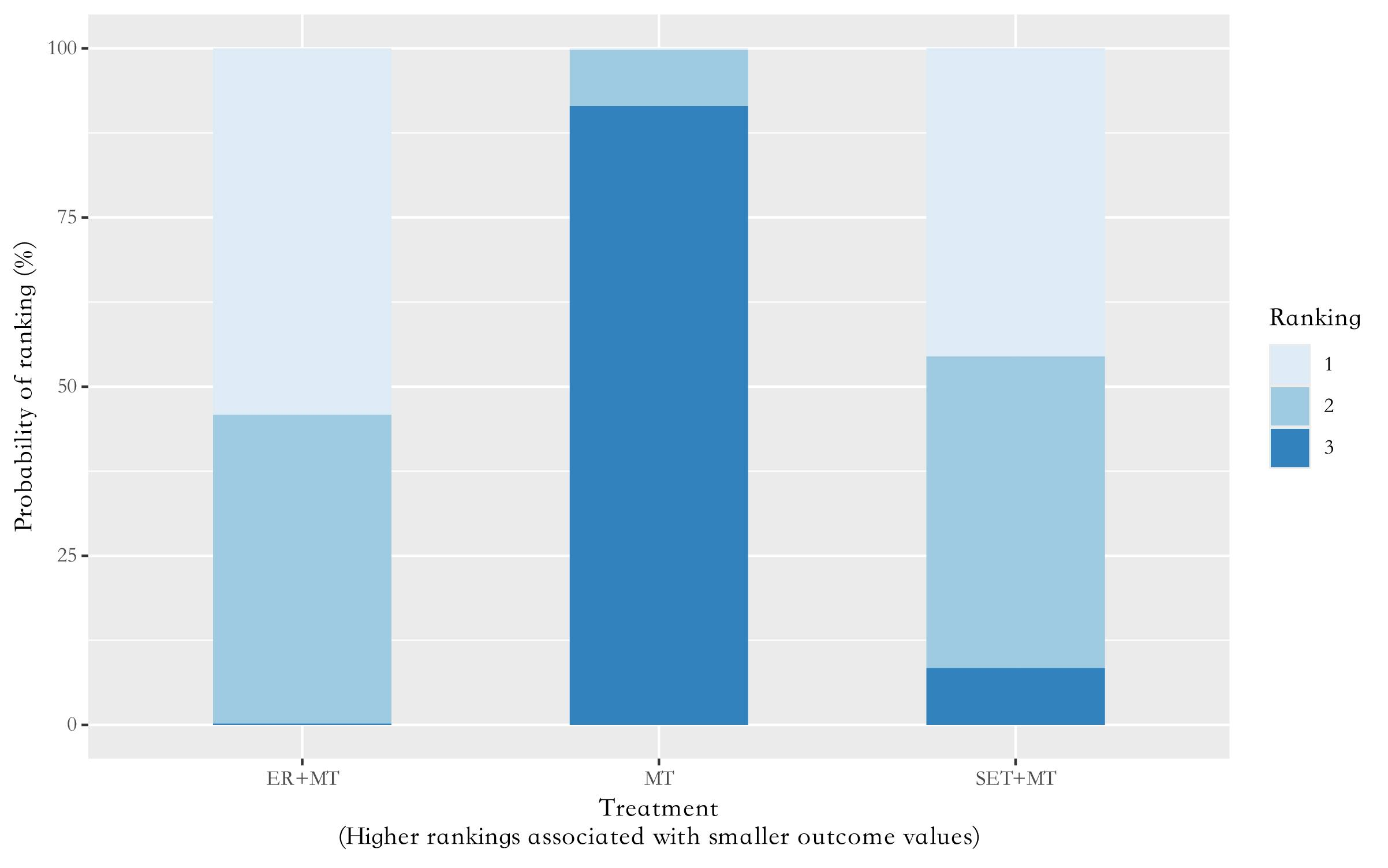

Supplement: Supplementary file 3 [file Data_Sheet_3.ZIP › Figure/Figure 11.tif]

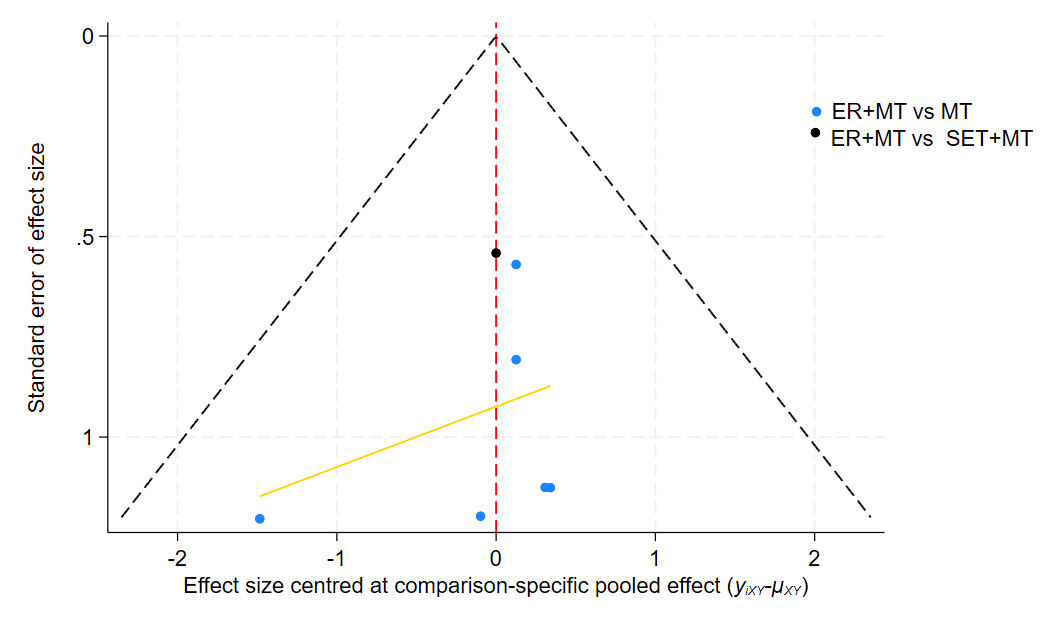

Supplement: Supplementary file 3 [file Data_Sheet_3.ZIP › Figure/Figure 12.tif]

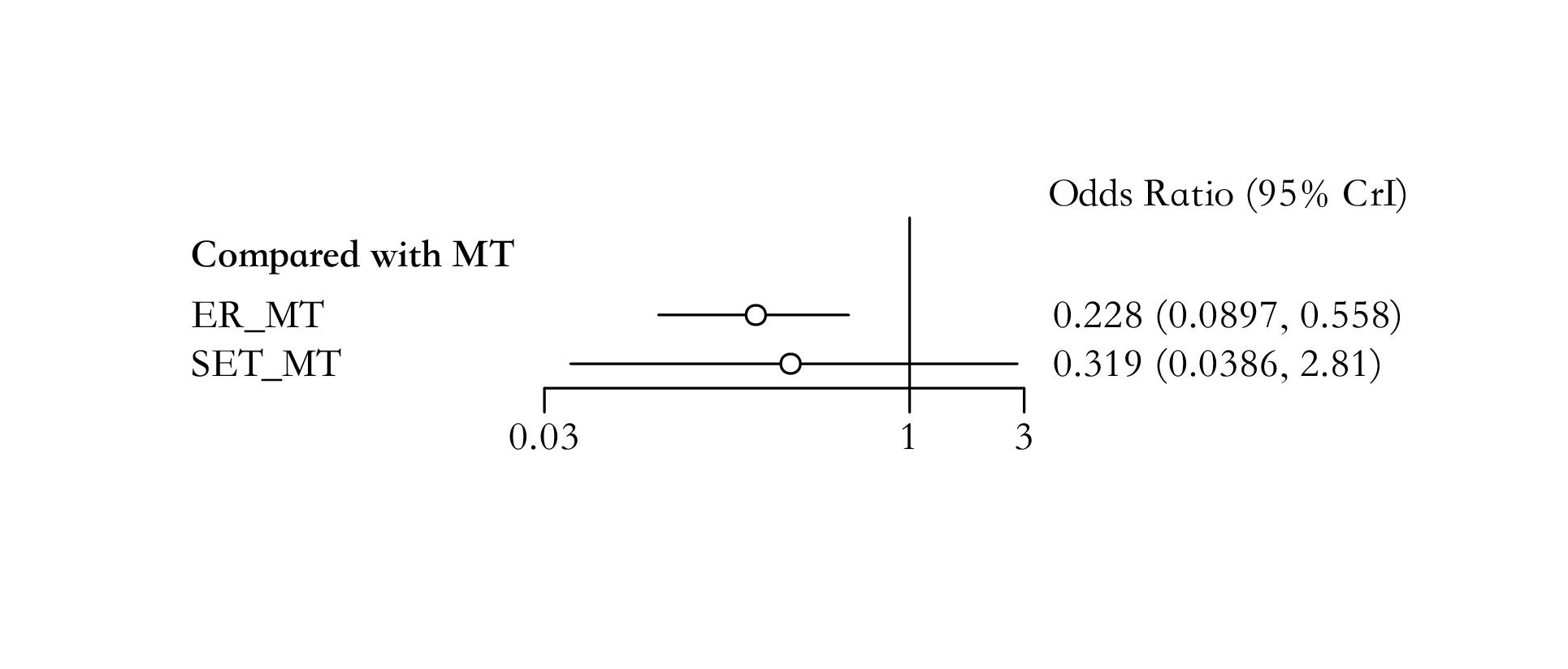

Supplement: Supplementary file 3 [file Data_Sheet_3.ZIP › Figure/Figure 13.tif]

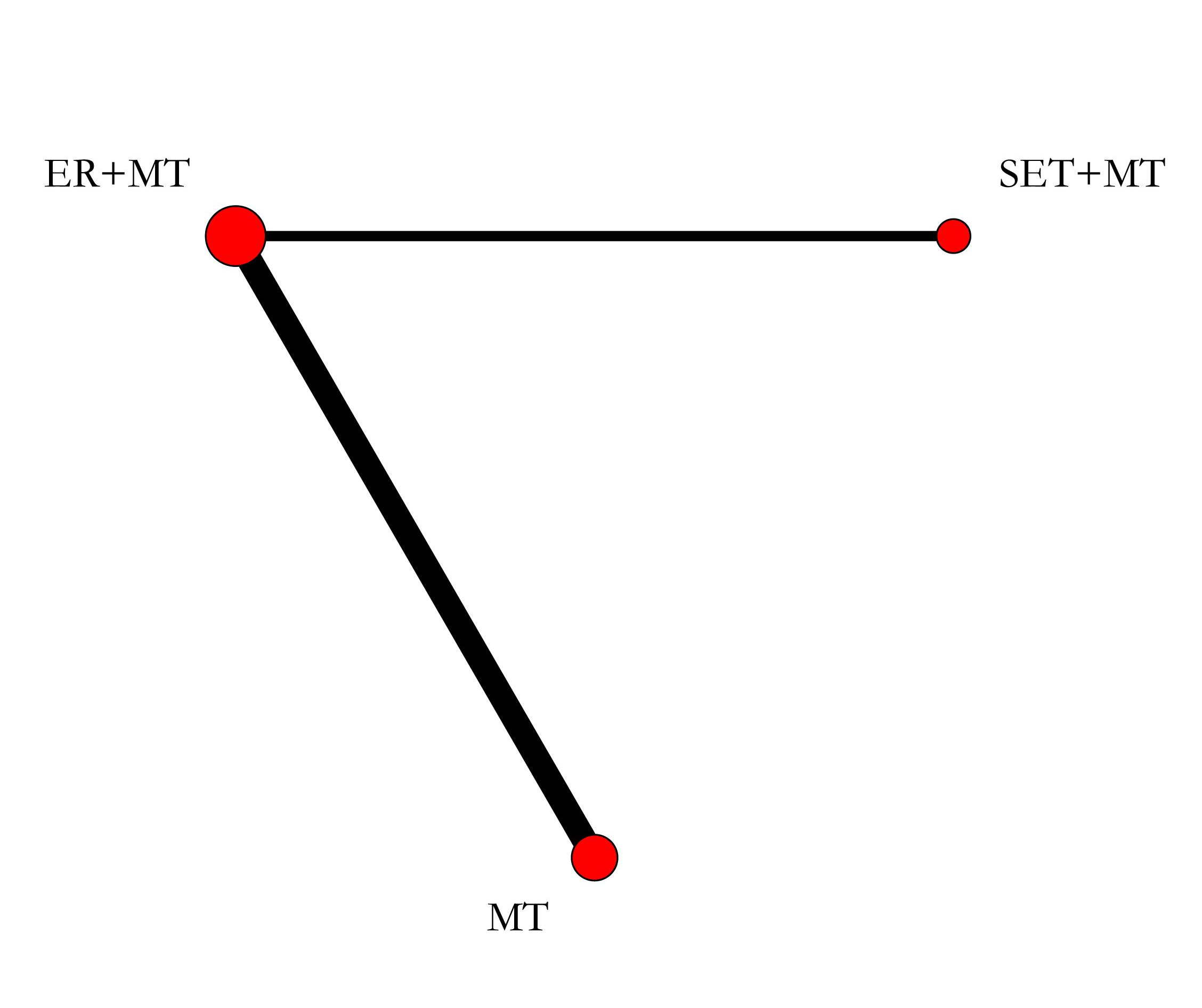

Supplement: Supplementary file 3 [file Data_Sheet_3.ZIP › Figure/Figure 14.tif]

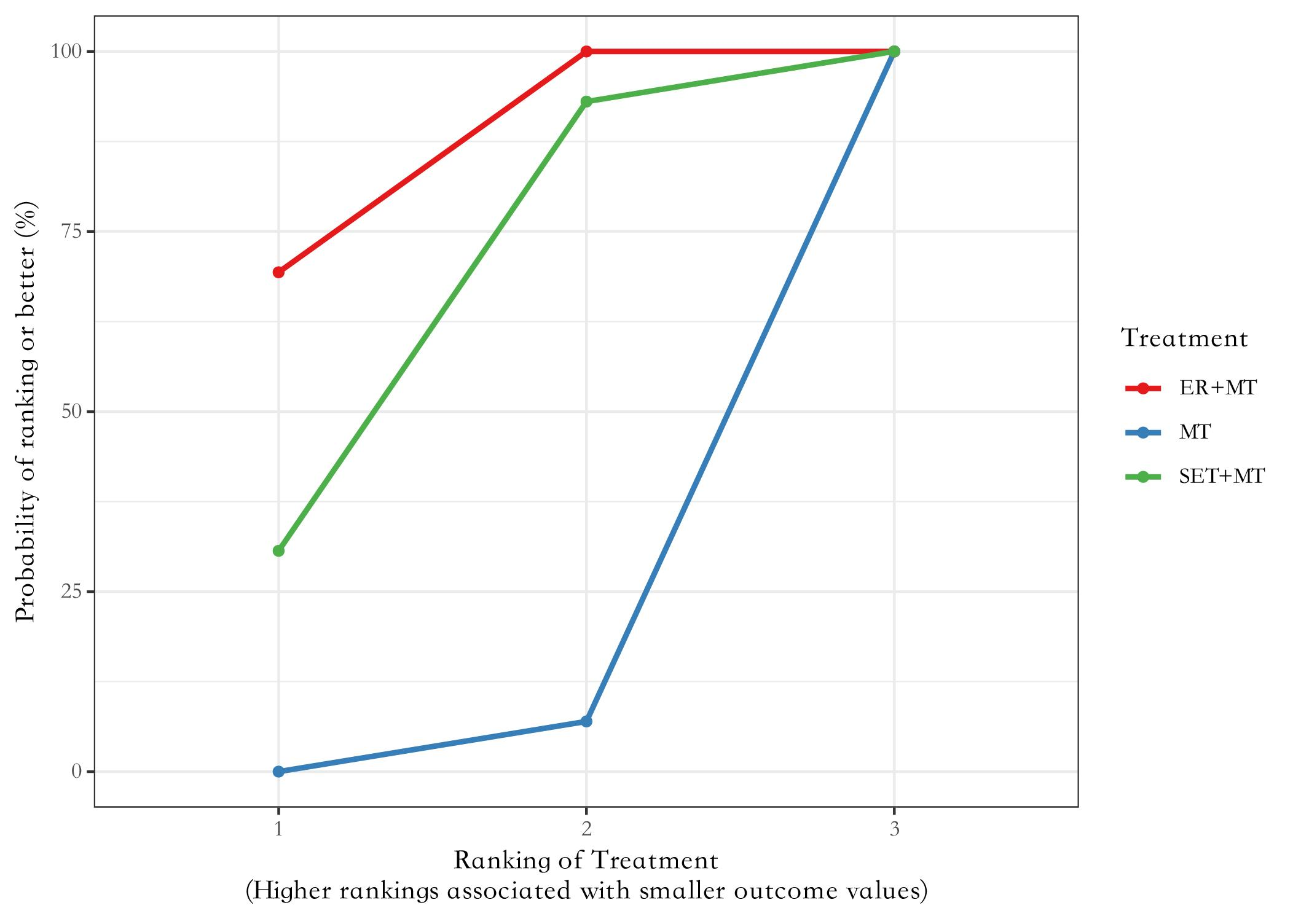

Supplement: Supplementary file 3 [file Data_Sheet_3.ZIP › Figure/Figure 15.tif]

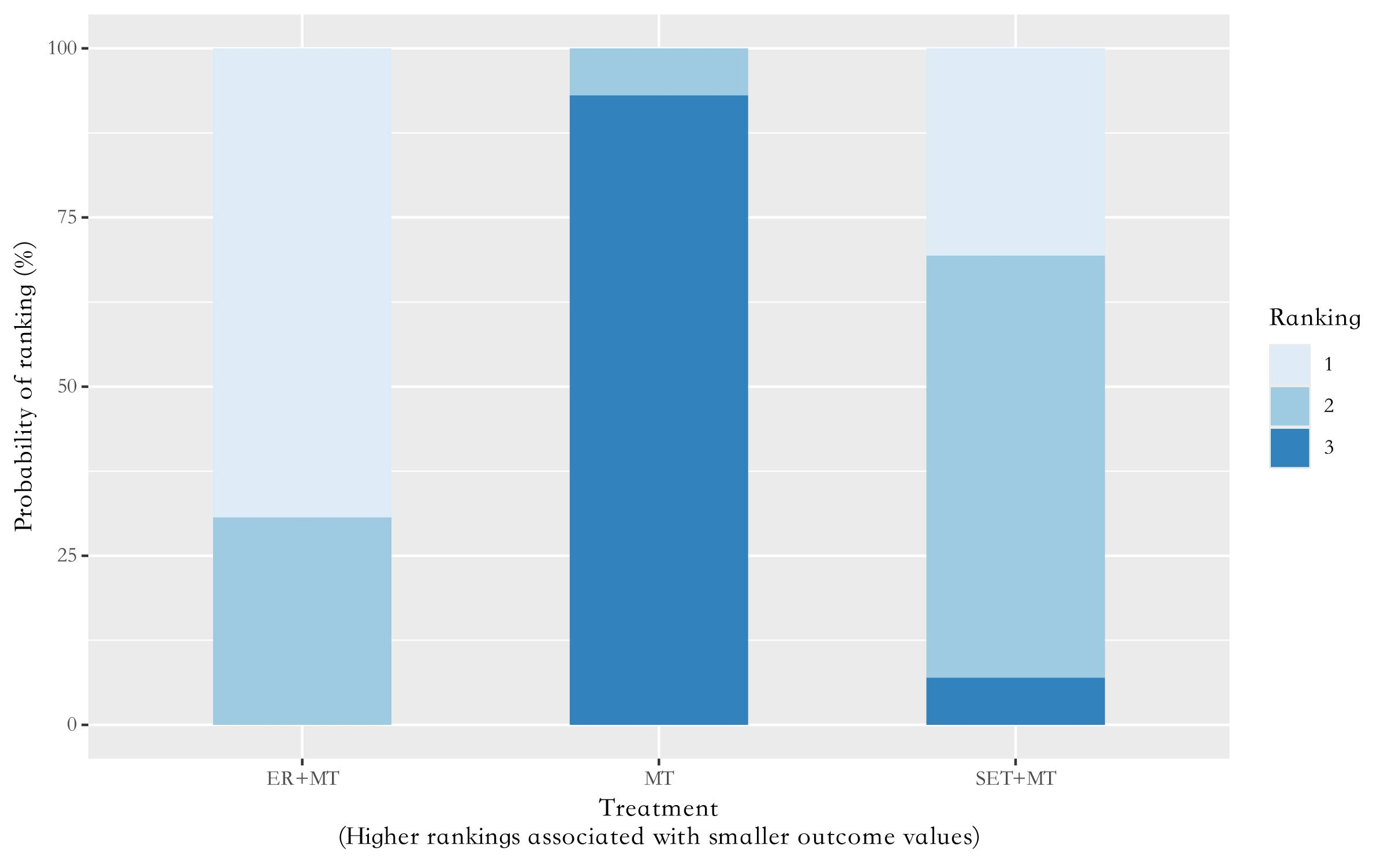

Supplement: Supplementary file 3 [file Data_Sheet_3.ZIP › Figure/Figure 16.tif]

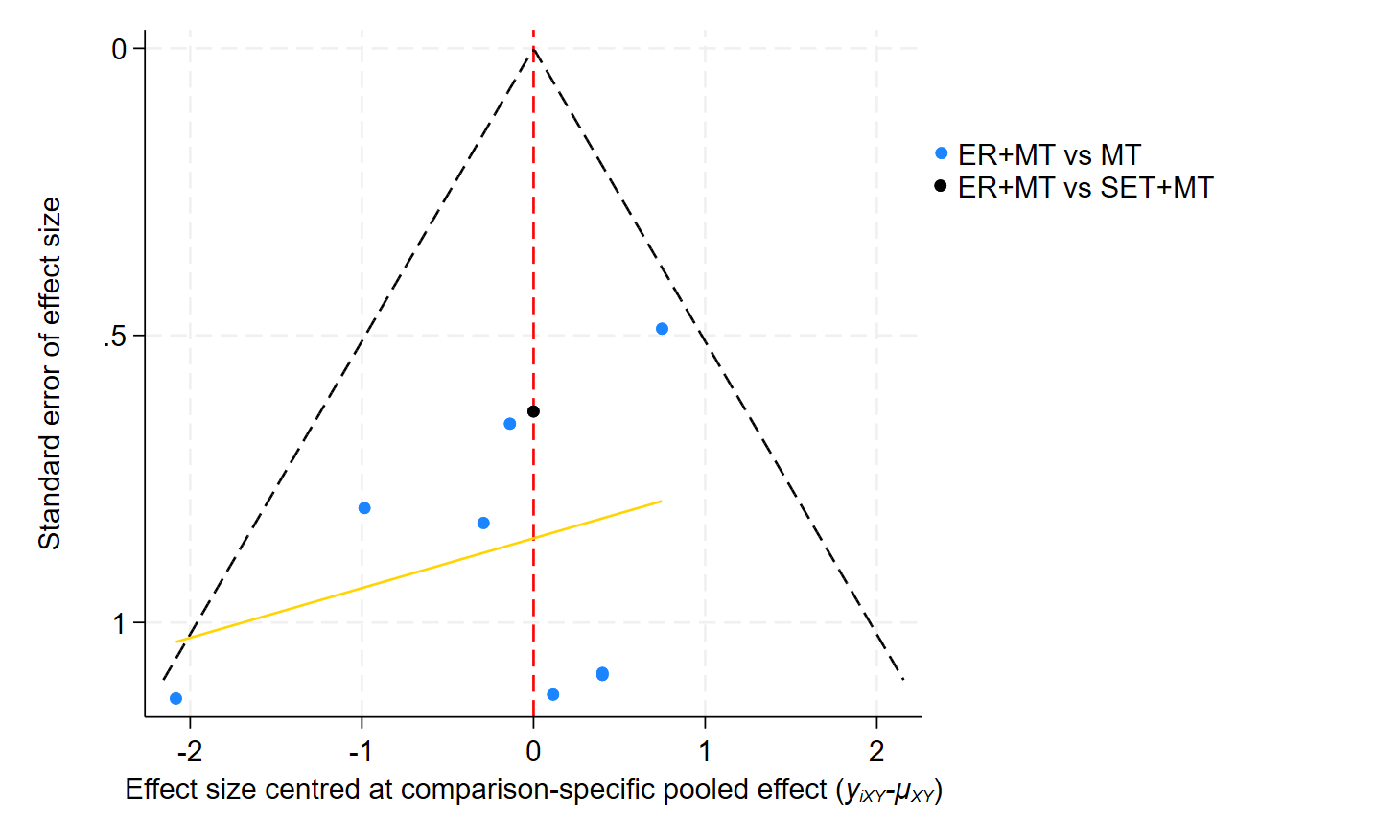

Supplement: Supplementary file 3 [file Data_Sheet_3.ZIP › Figure/Figure 17.tif]

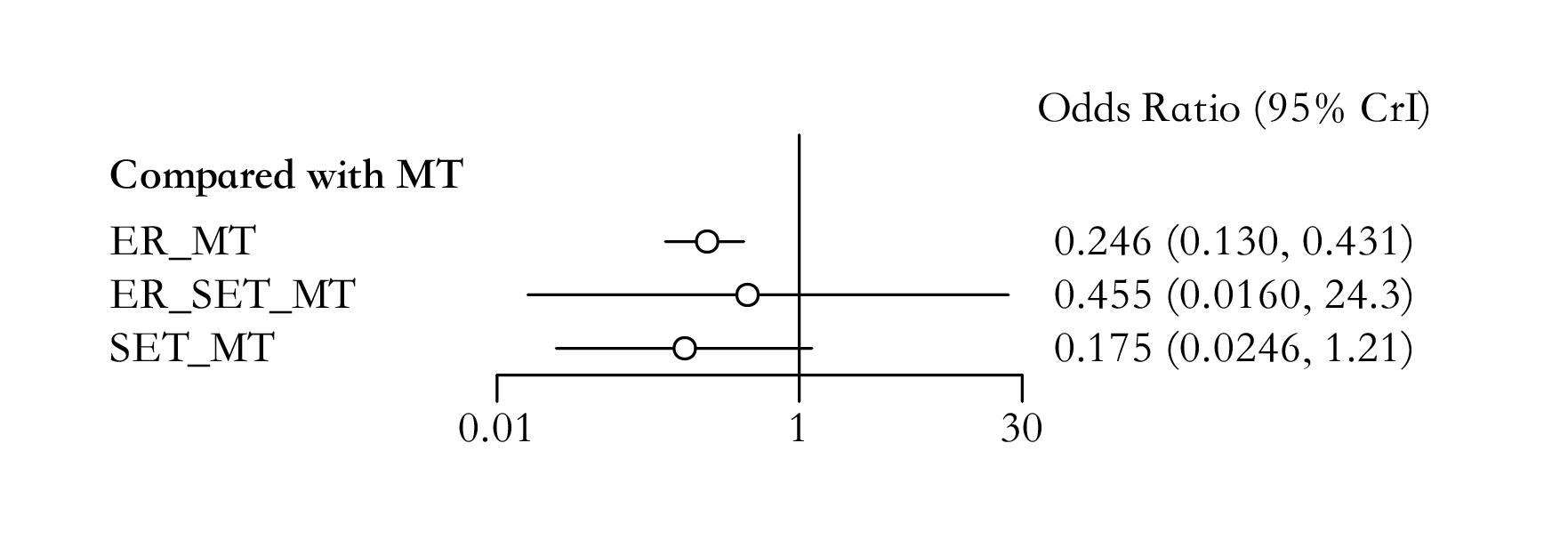

Supplement: Supplementary file 3 [file Data_Sheet_3.ZIP › Figure/Figure 18.tif]

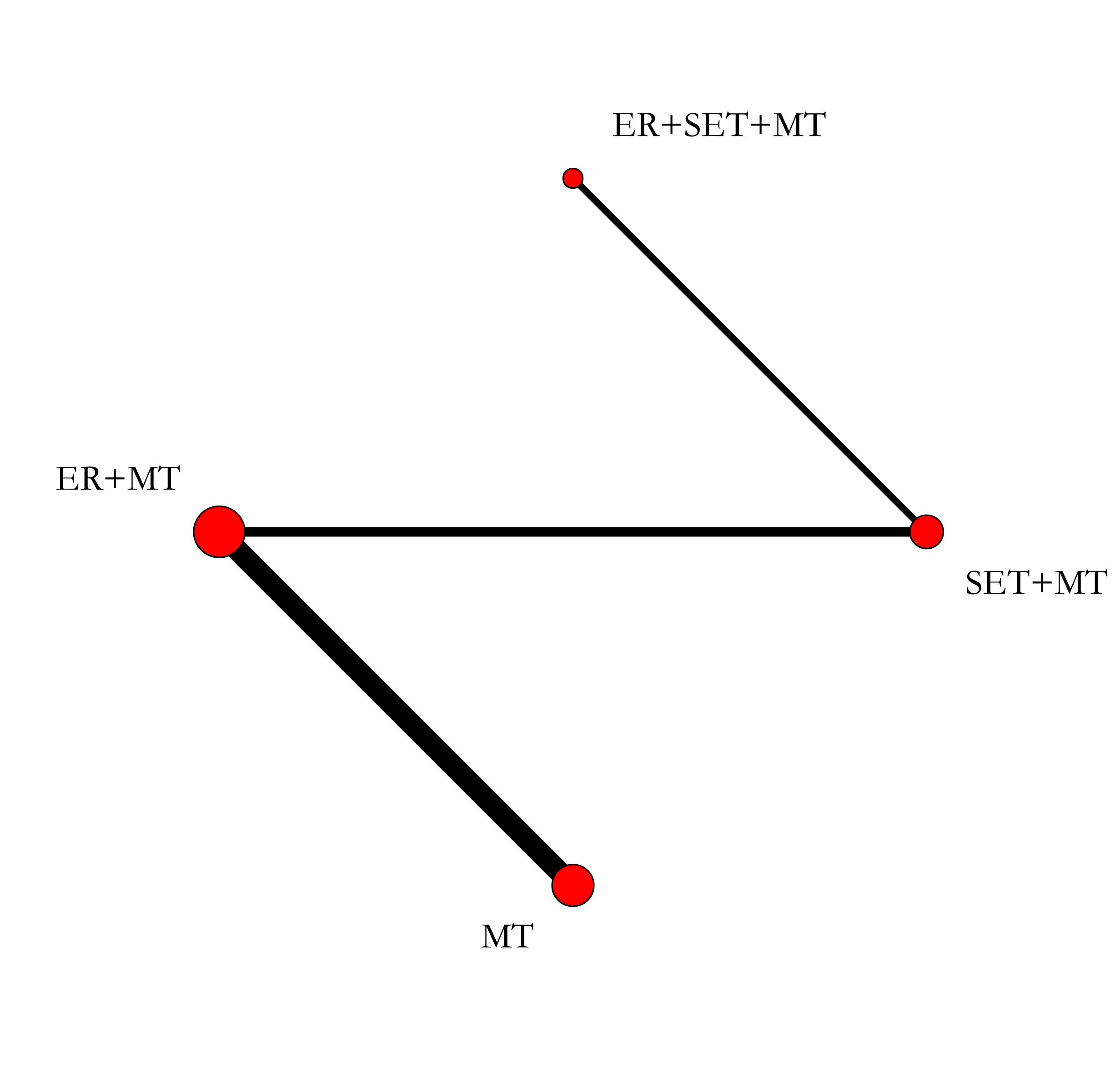

Supplement: Supplementary file 3 [file Data_Sheet_3.ZIP › Figure/Figure 19.tif]

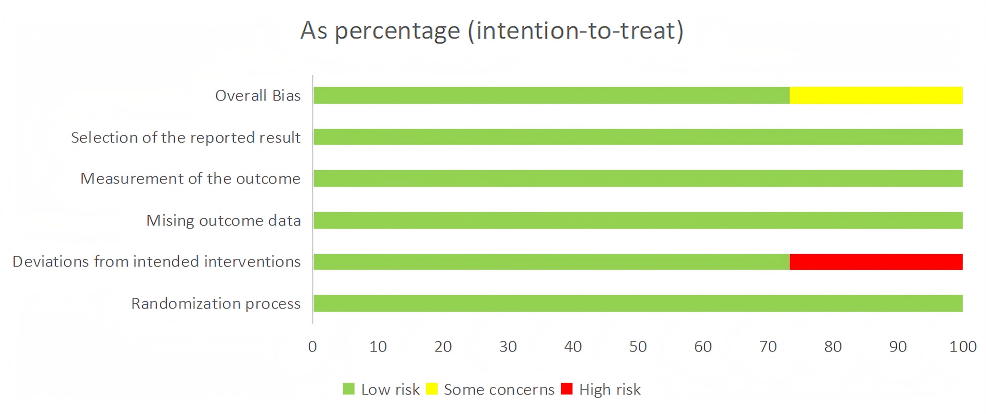

Supplement: Supplementary file 3 [file Data_Sheet_3.ZIP › Figure/Figure 2.tif]

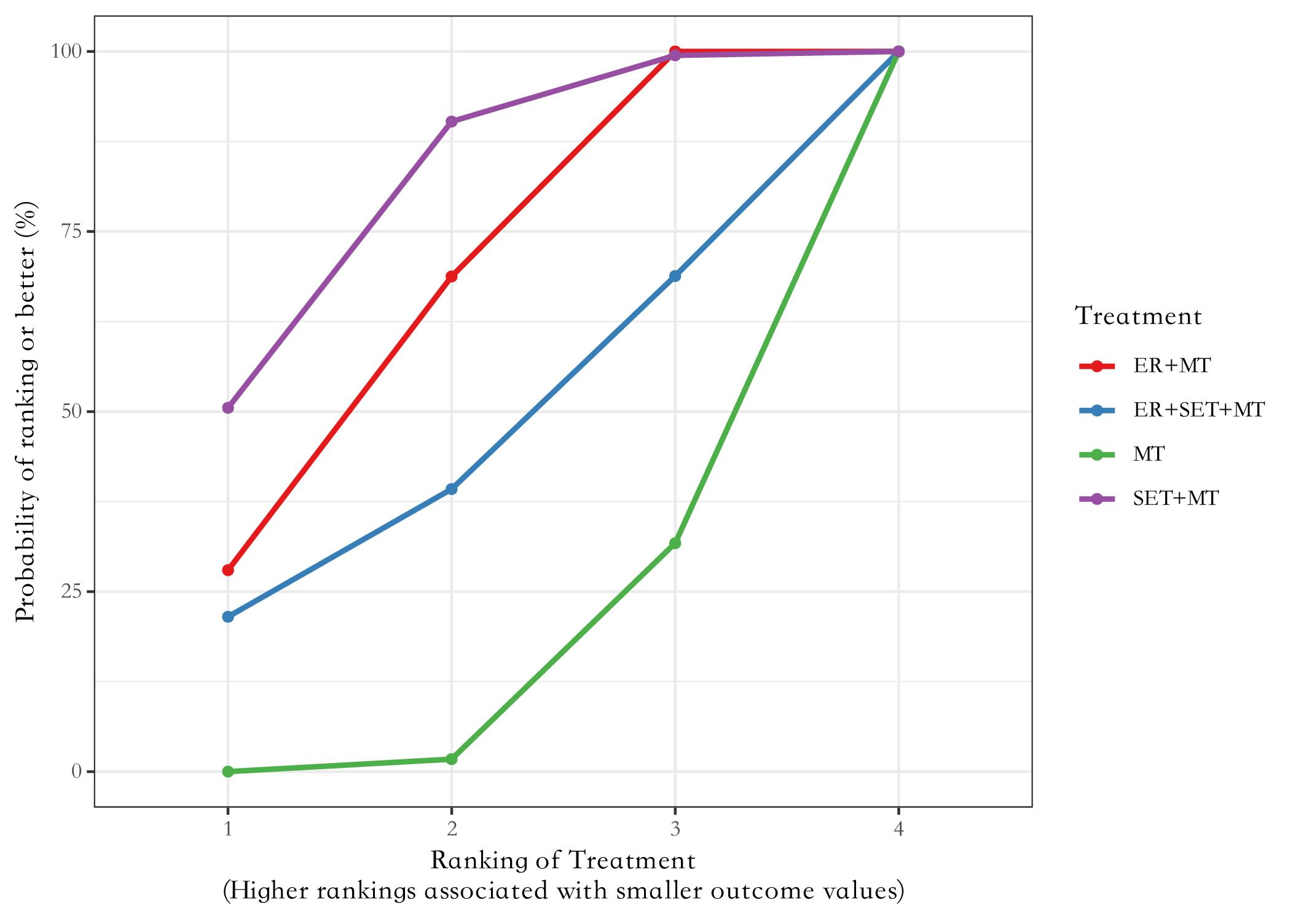

Supplement: Supplementary file 3 [file Data_Sheet_3.ZIP › Figure/Figure 20.tif]

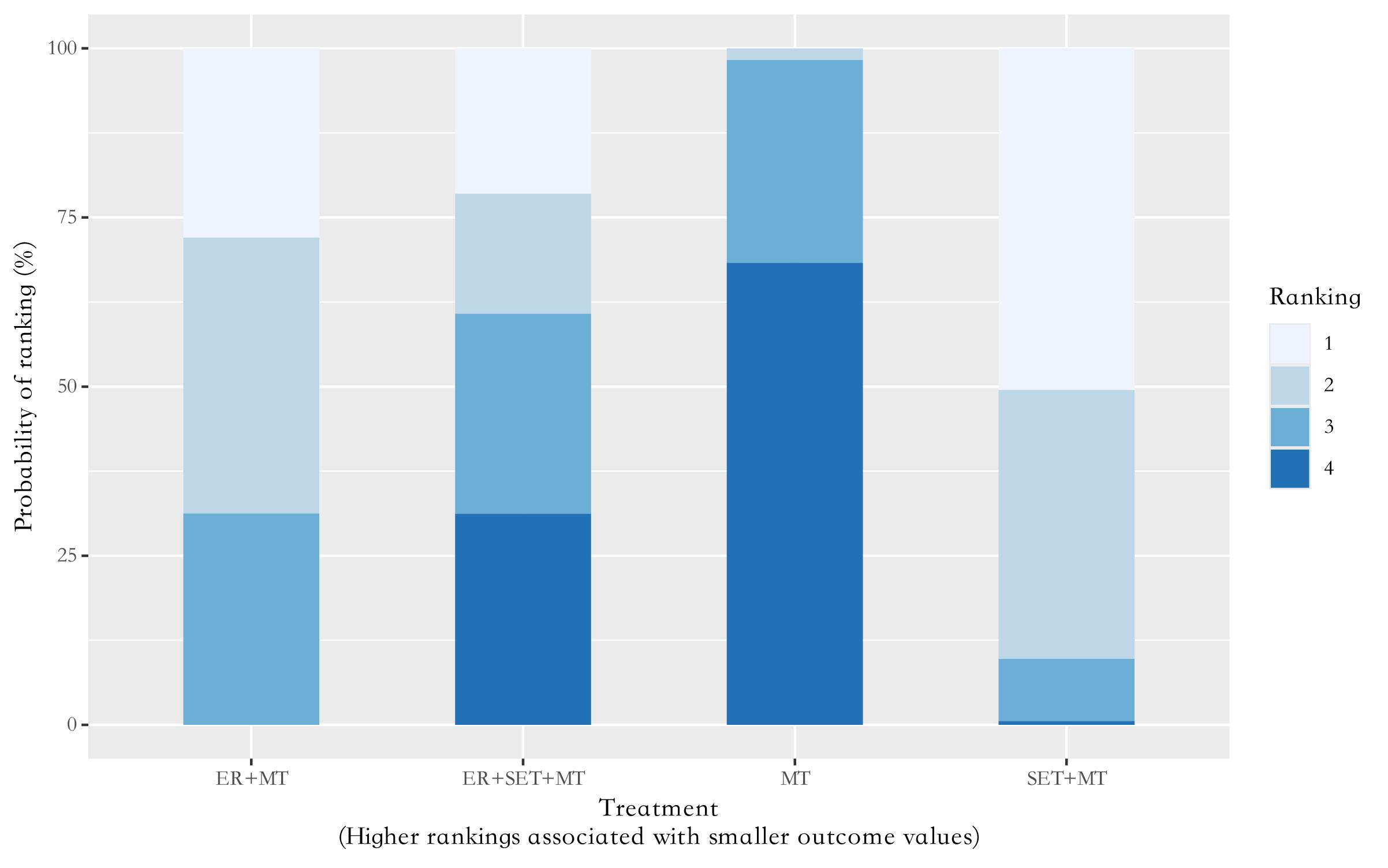

Supplement: Supplementary file 3 [file Data_Sheet_3.ZIP › Figure/Figure 21.tif]

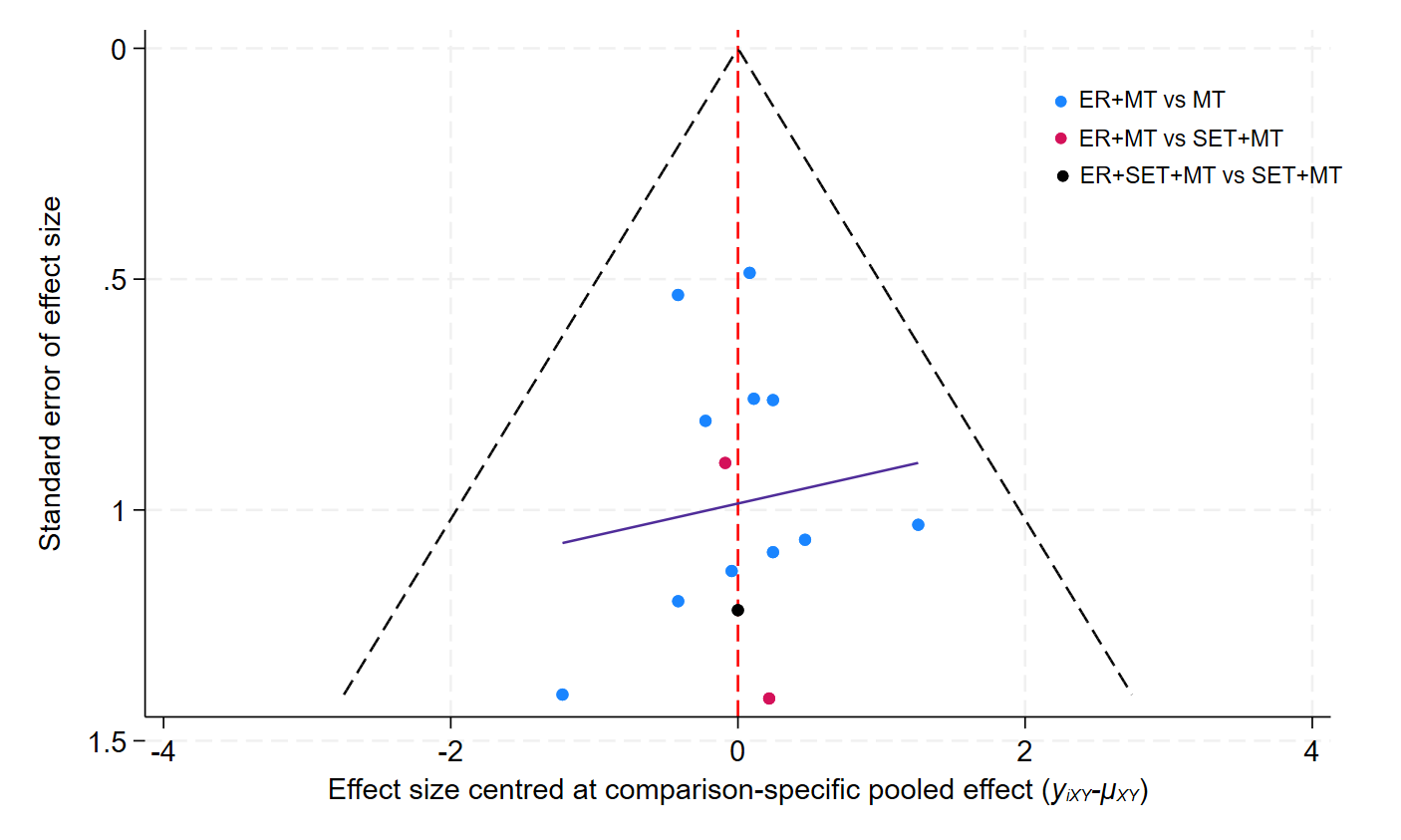

Supplement: Supplementary file 3 [file Data_Sheet_3.ZIP › Figure/Figure 22.tif]

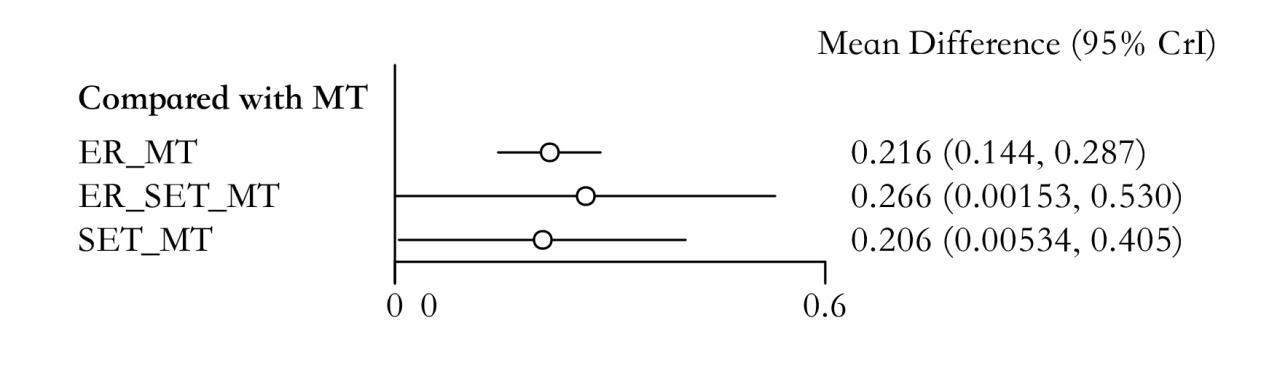

Supplement: Supplementary file 3 [file Data_Sheet_3.ZIP › Figure/Figure 23.tif]

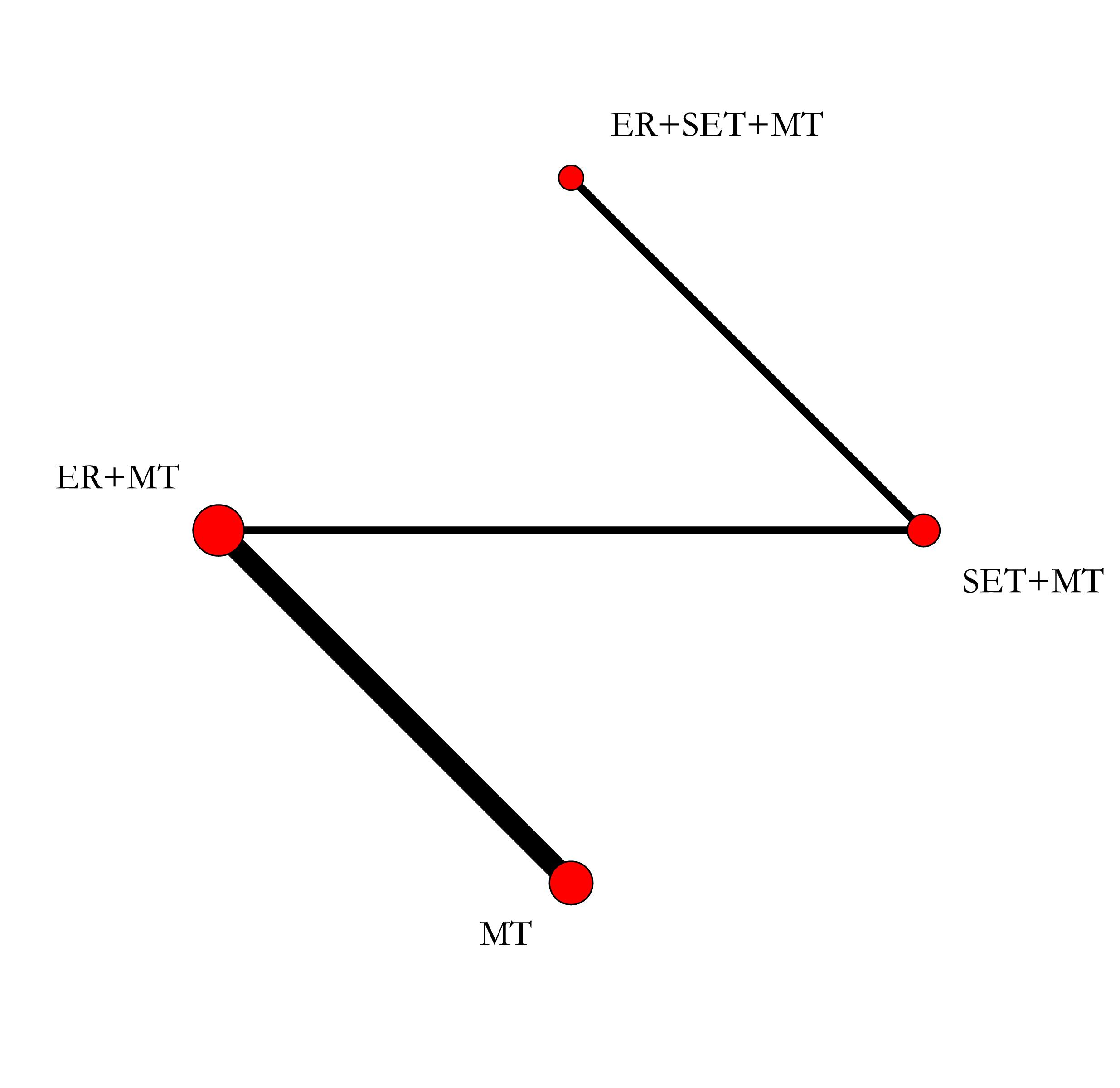

Supplement: Supplementary file 3 [file Data_Sheet_3.ZIP › Figure/Figure 24.tif]

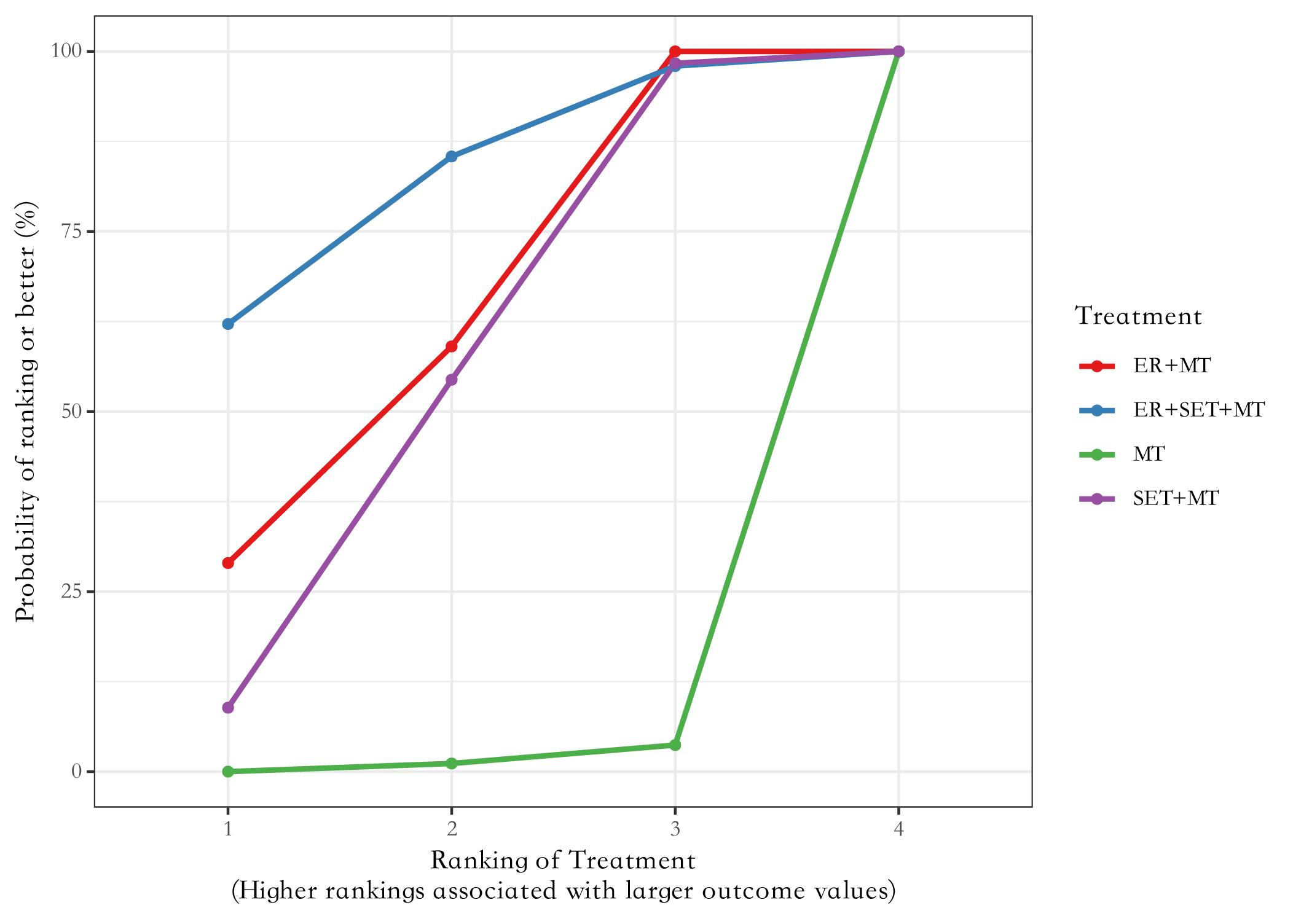

Supplement: Supplementary file 3 [file Data_Sheet_3.ZIP › Figure/Figure 25.tif]

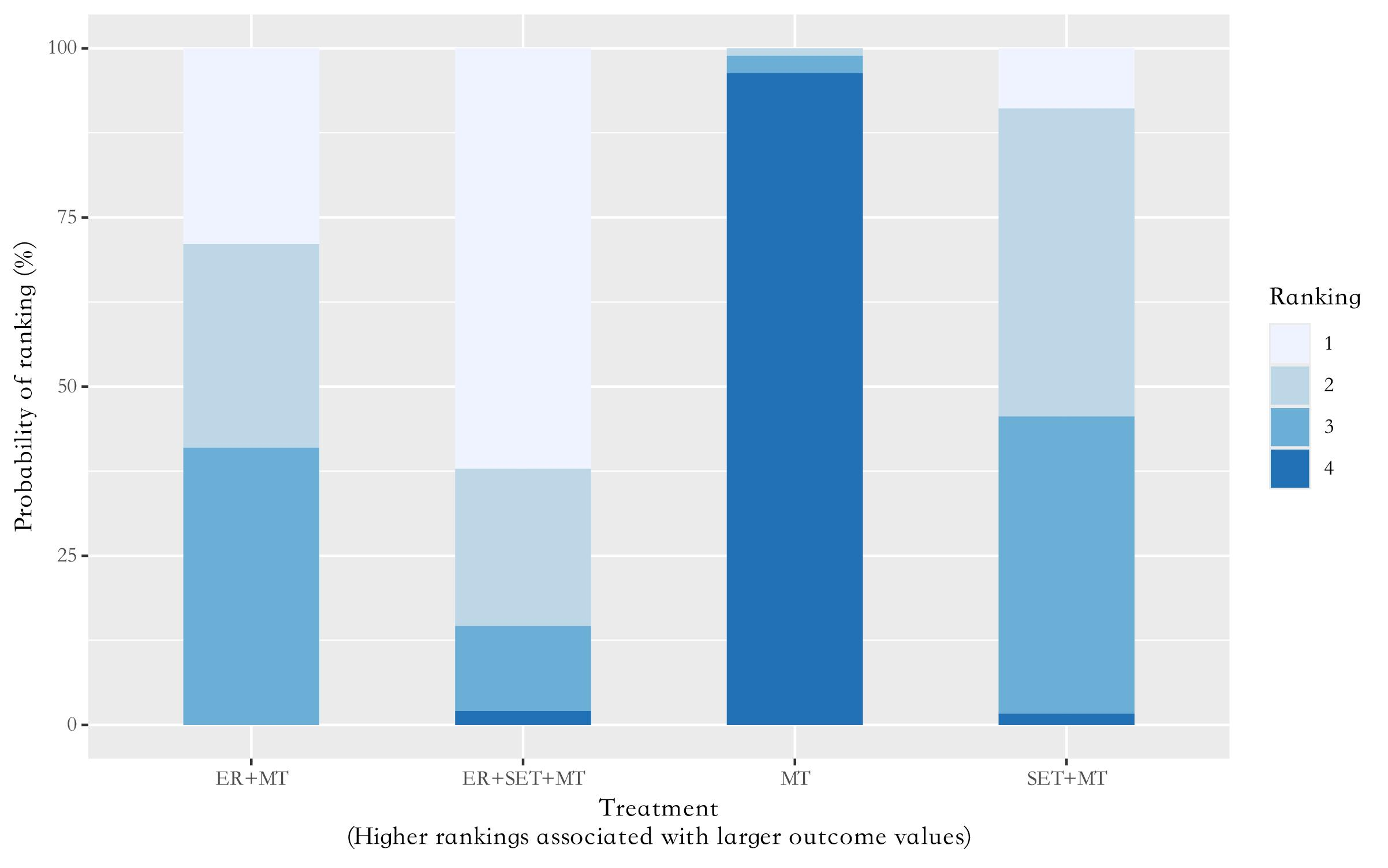

Supplement: Supplementary file 3 [file Data_Sheet_3.ZIP › Figure/Figure 26.tif]

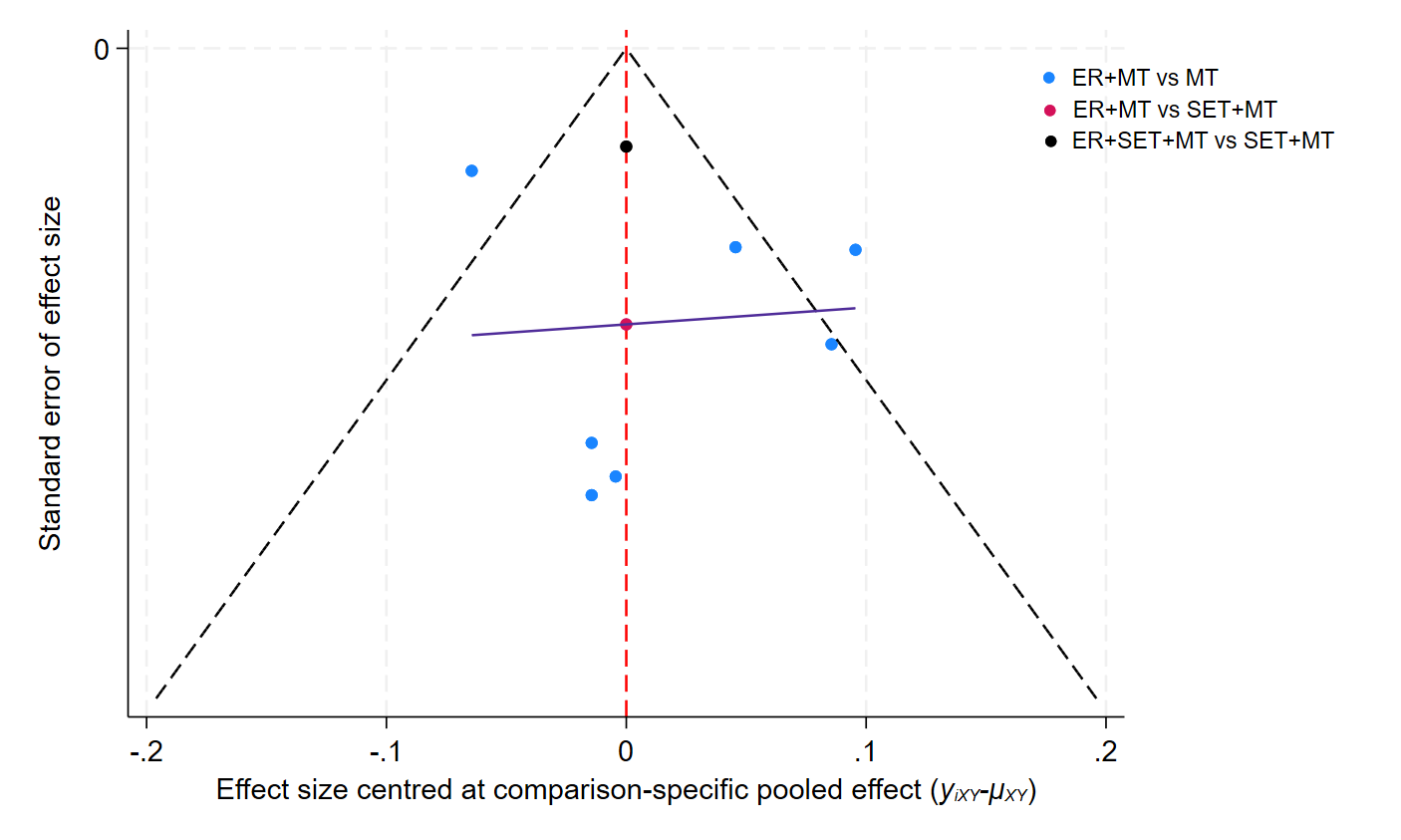

Supplement: Supplementary file 3 [file Data_Sheet_3.ZIP › Figure/Figure 27.tif]

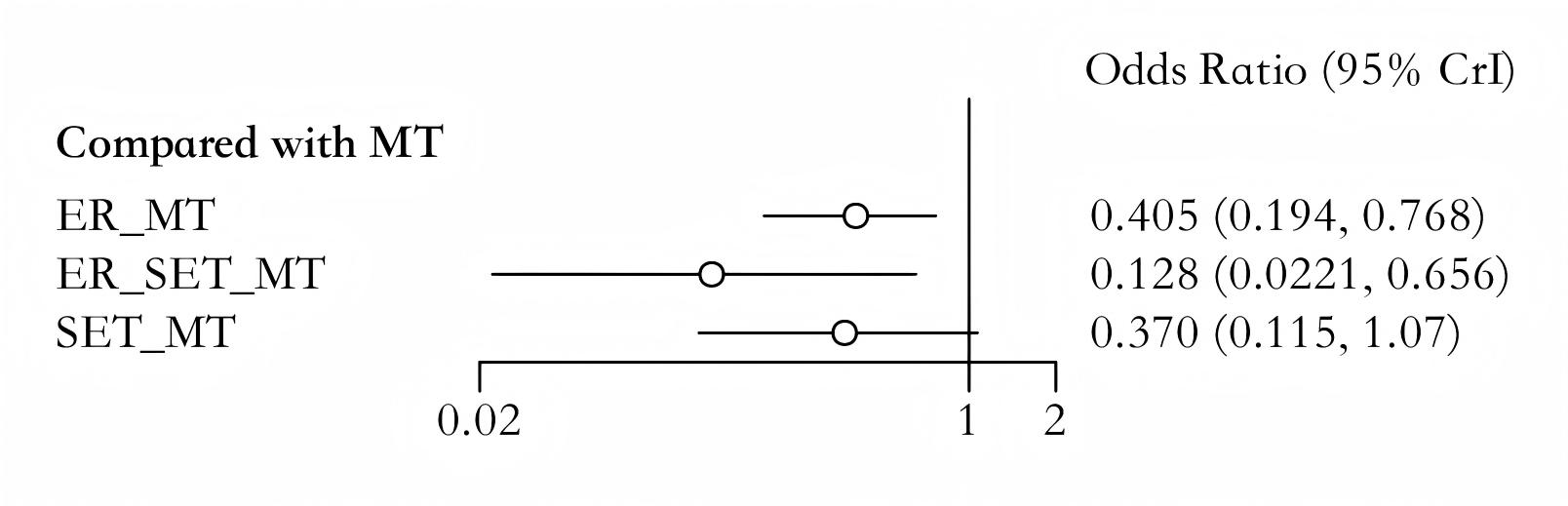

Supplement: Supplementary file 3 [file Data_Sheet_3.ZIP › Figure/Figure 3.tif]

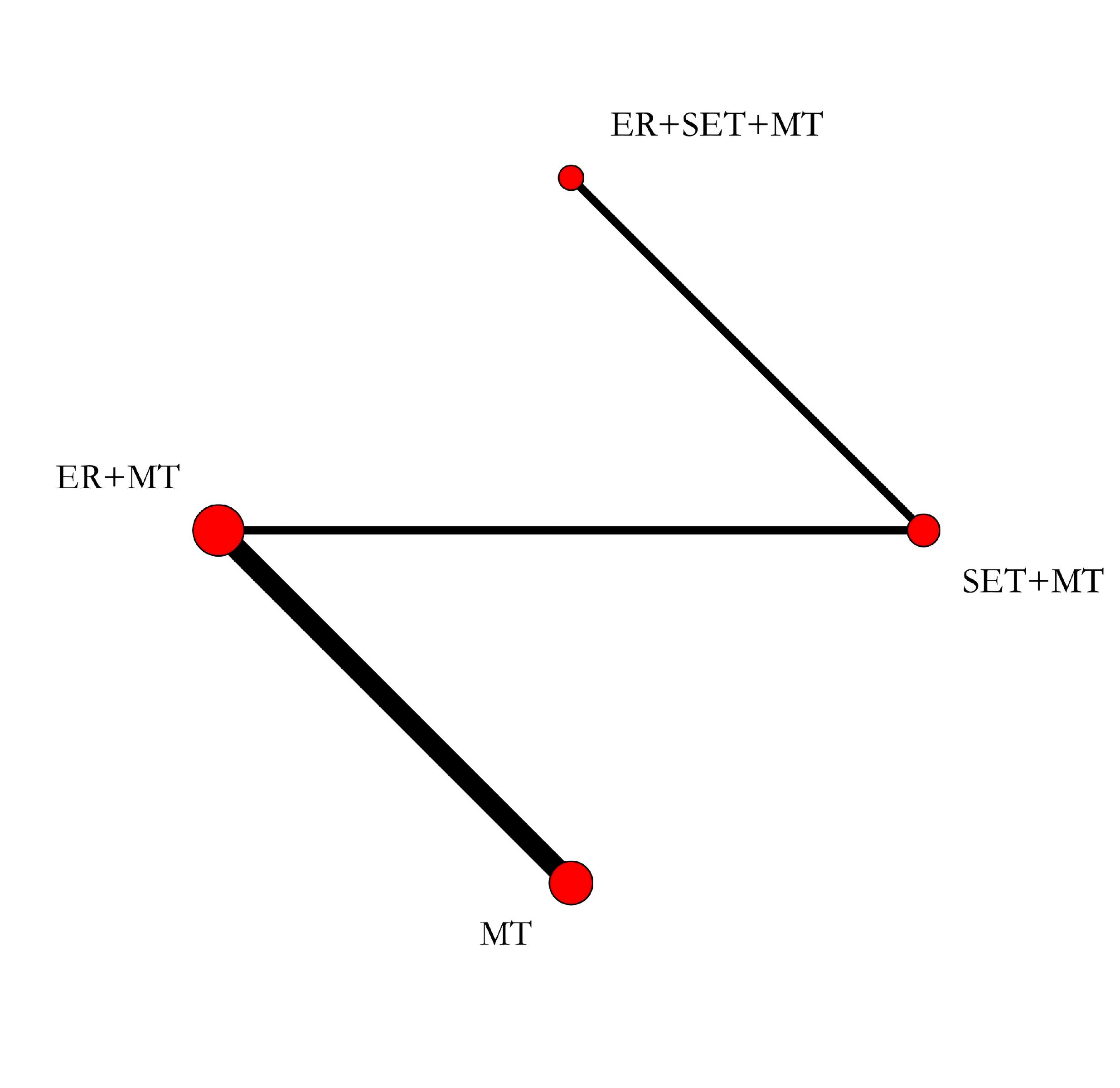

Supplement: Supplementary file 3 [file Data_Sheet_3.ZIP › Figure/Figure 4.tif]

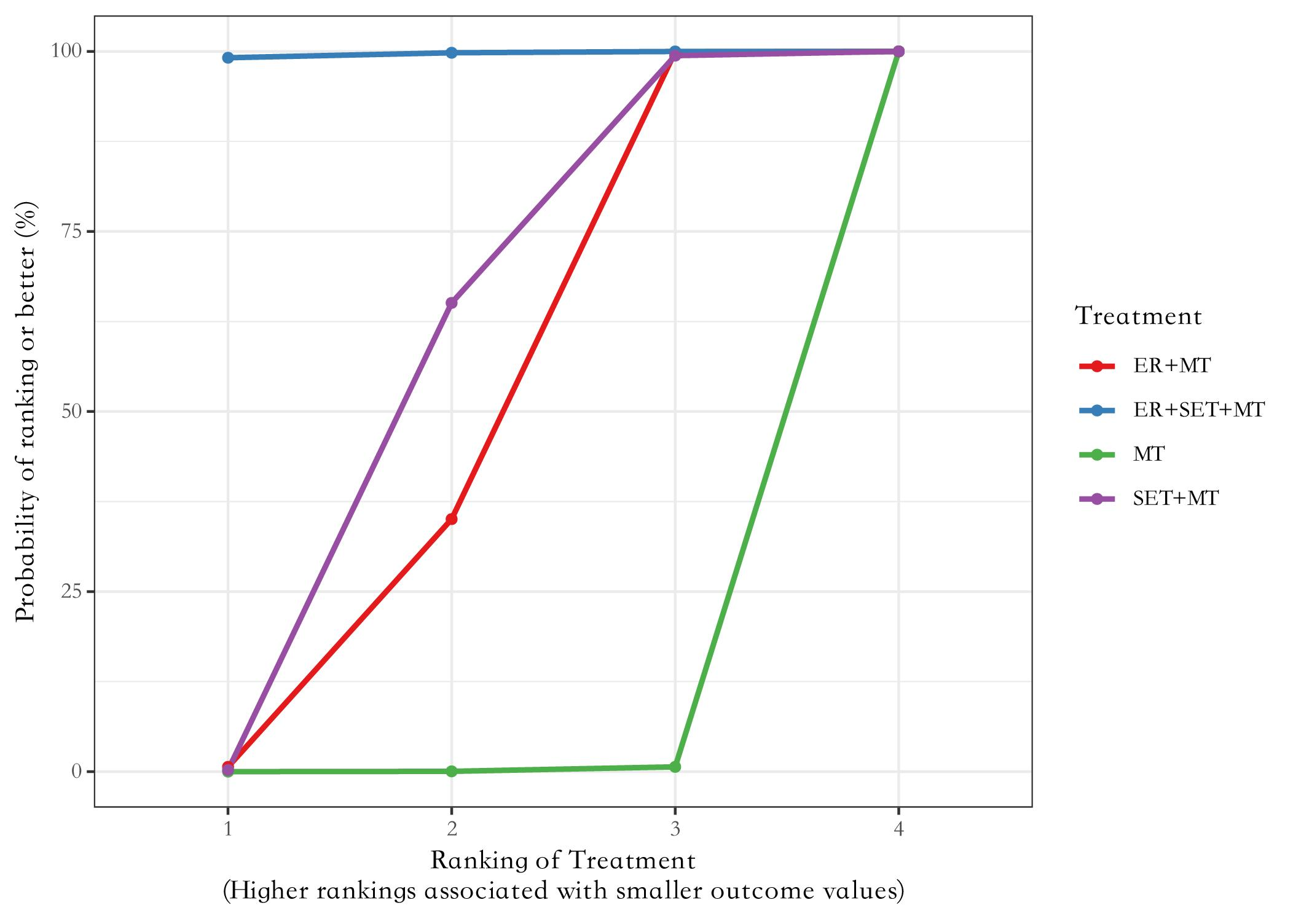

Supplement: Supplementary file 3 [file Data_Sheet_3.ZIP › Figure/Figure 5.tif]

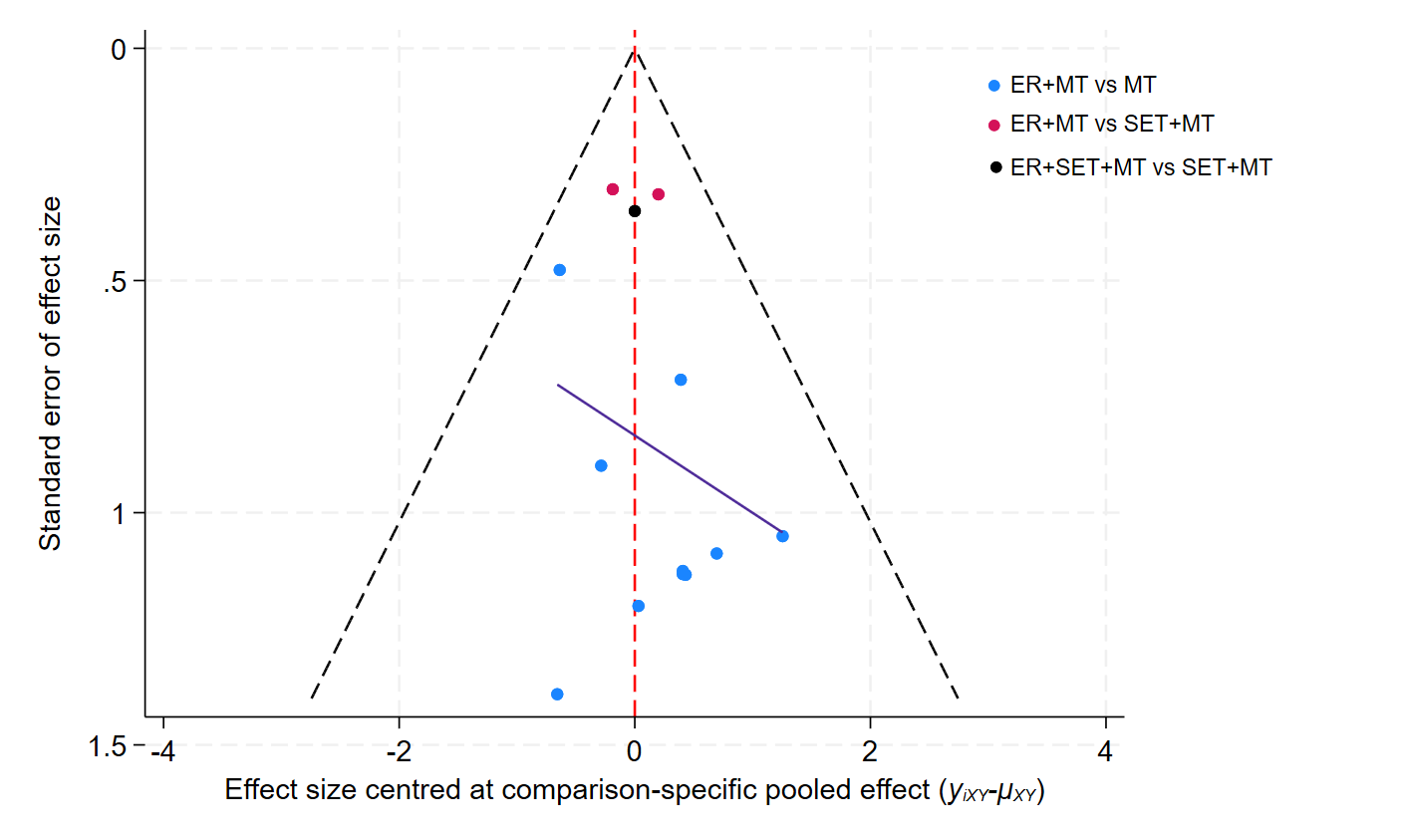

Supplement: Supplementary file 3 [file Data_Sheet_3.ZIP › Figure/Figure 7.tif]

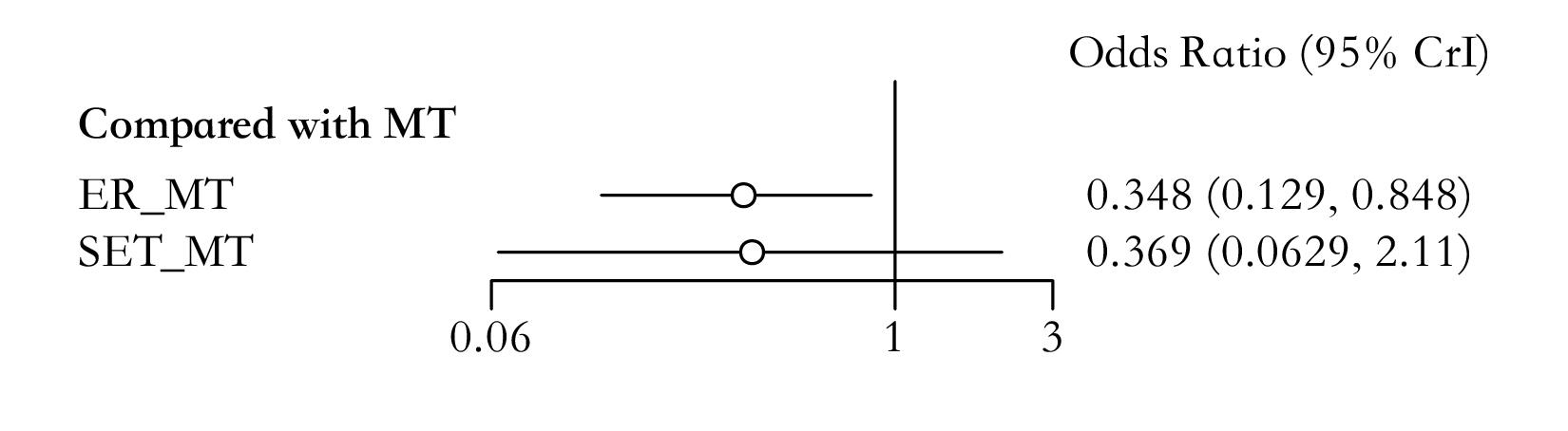

Supplement: Supplementary file 3 [file Data_Sheet_3.ZIP › Figure/Figure 8.tif]

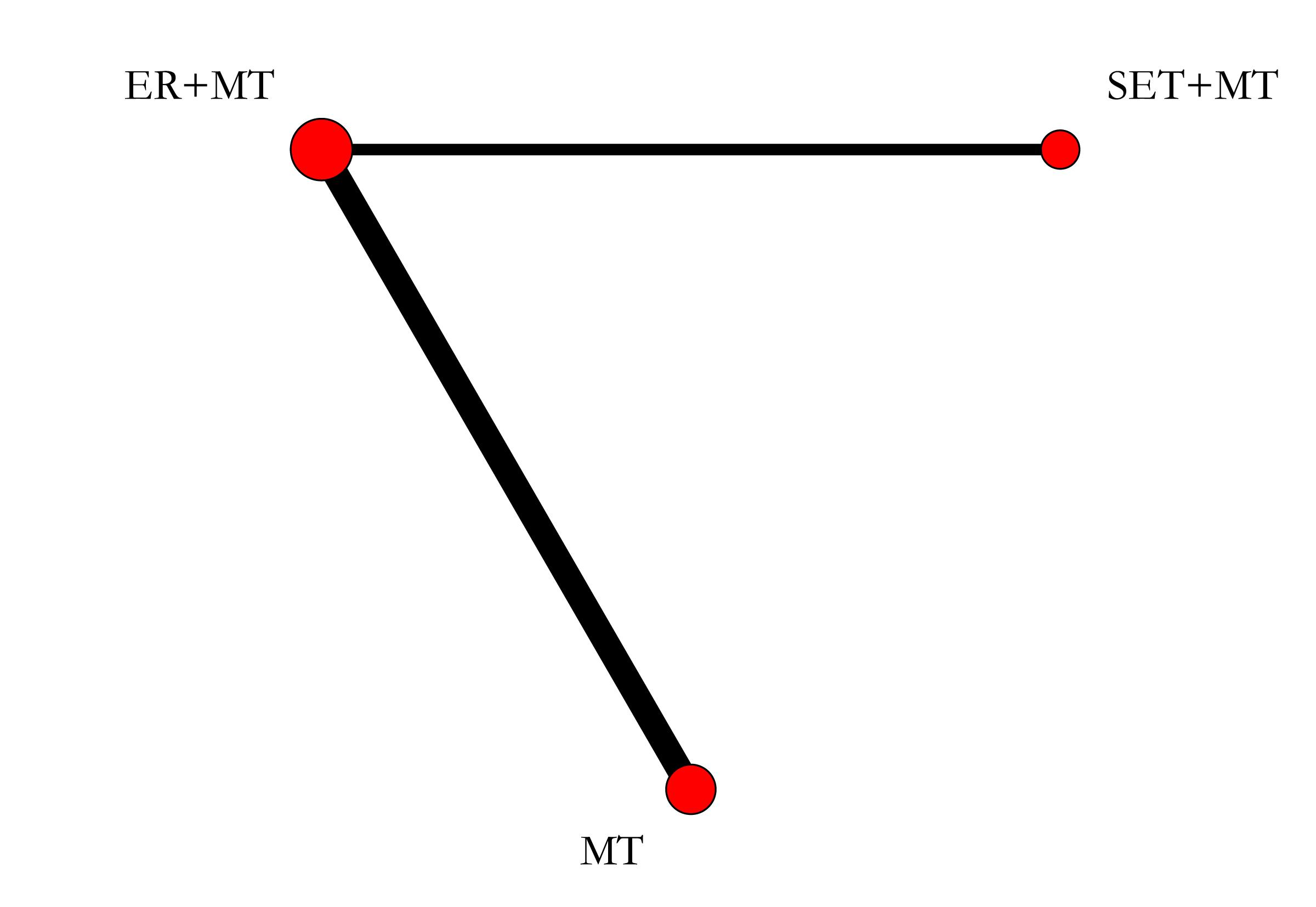

Supplement: Supplementary file 3 [file Data_Sheet_3.ZIP › Figure/Figure 9.tif]

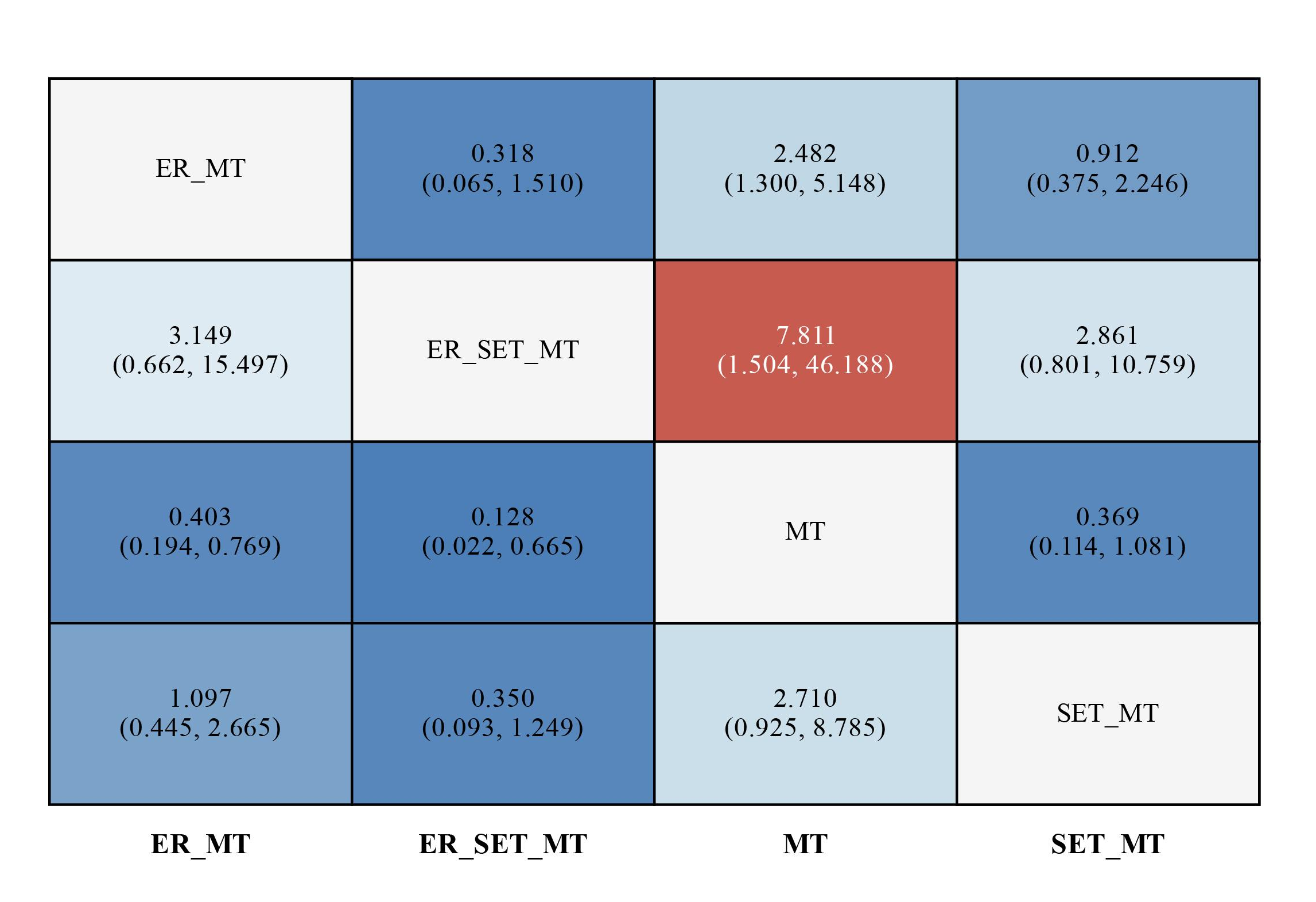

Supplement: Supplementary file 3 [file Data_Sheet_3.ZIP › Table/Table 2.tif]

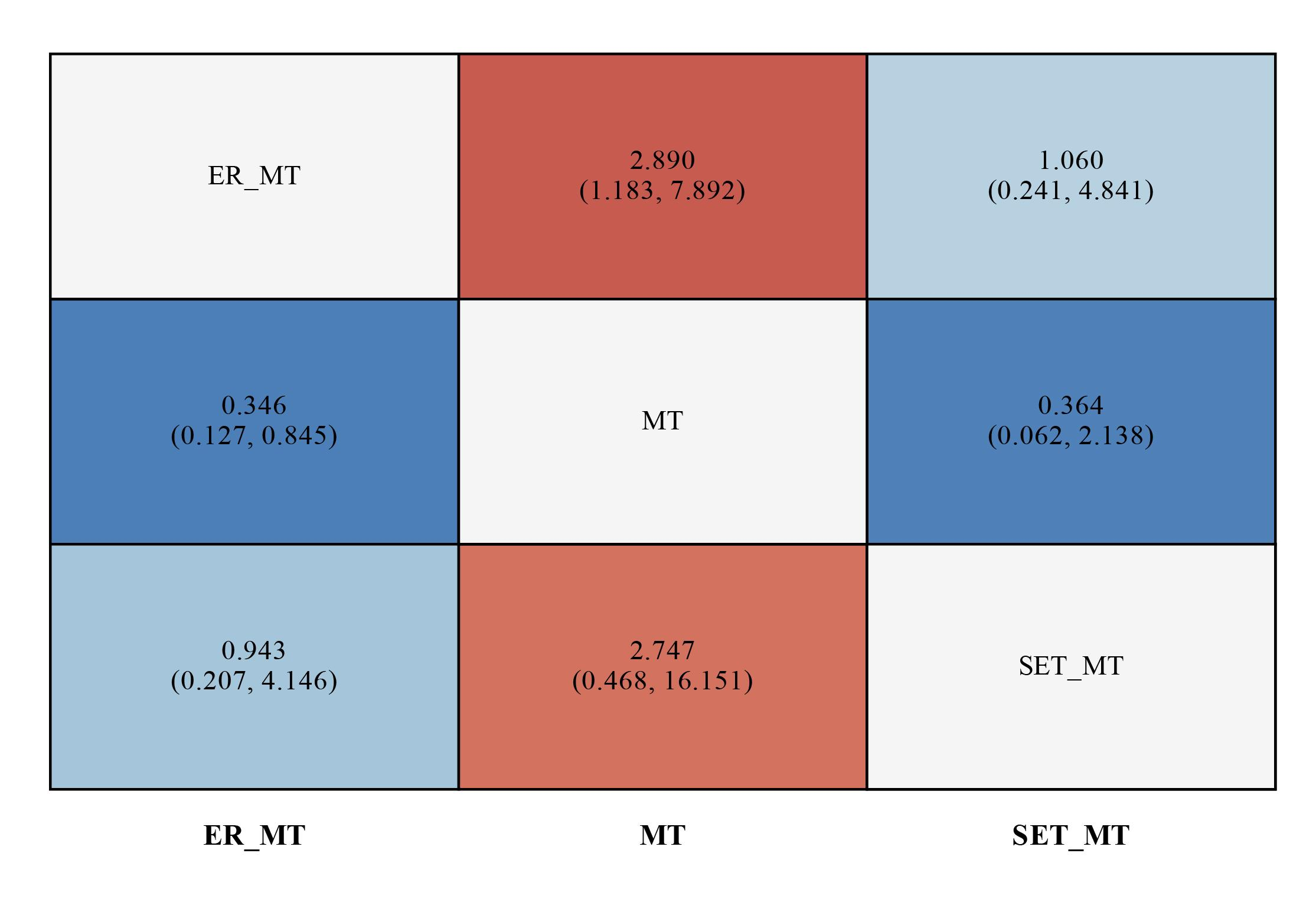

Supplement: Supplementary file 3 [file Data_Sheet_3.ZIP › Table/Table 3.tif]

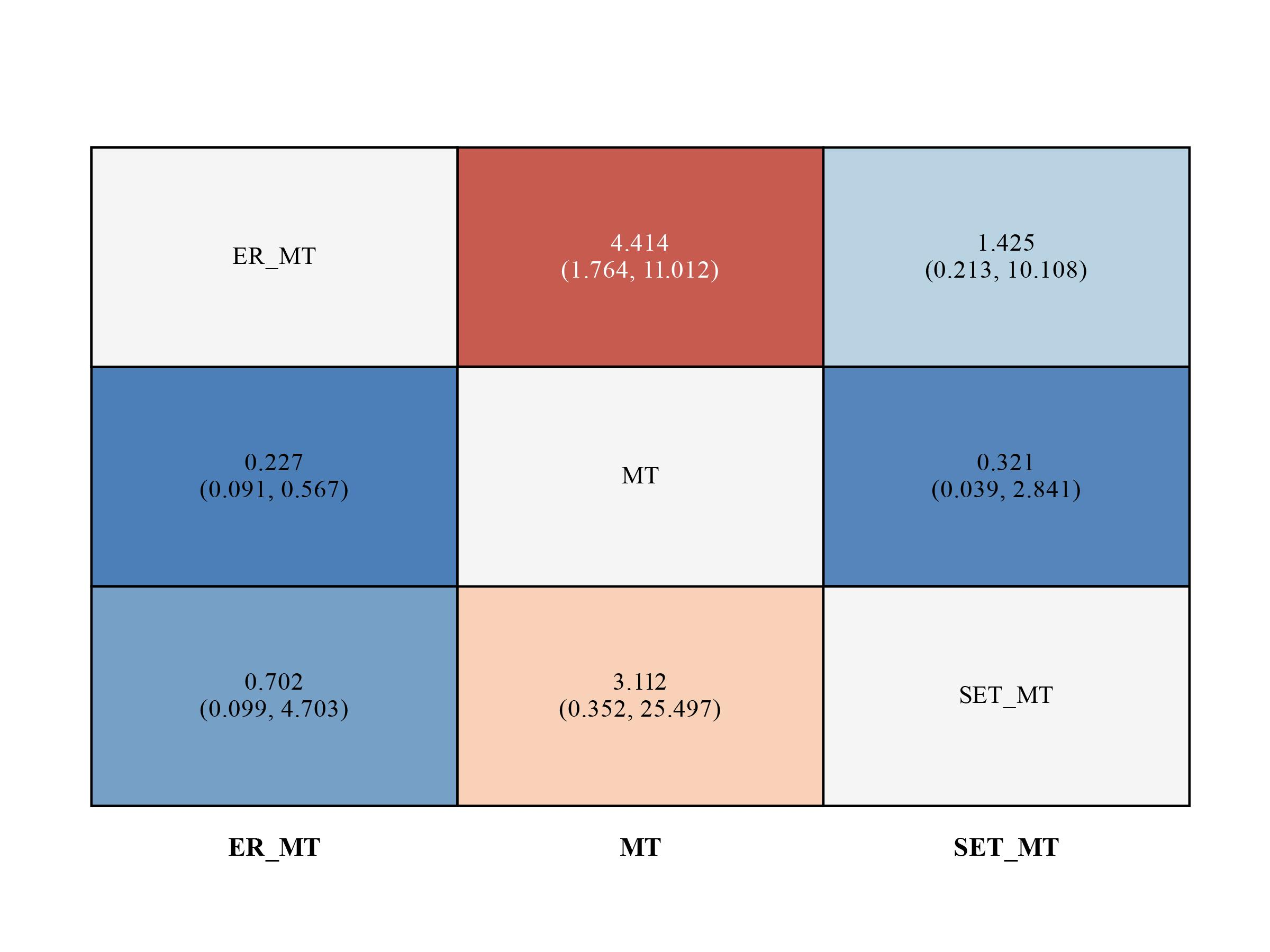

Supplement: Supplementary file 3 [file Data_Sheet_3.ZIP › Table/Table 4.tif]

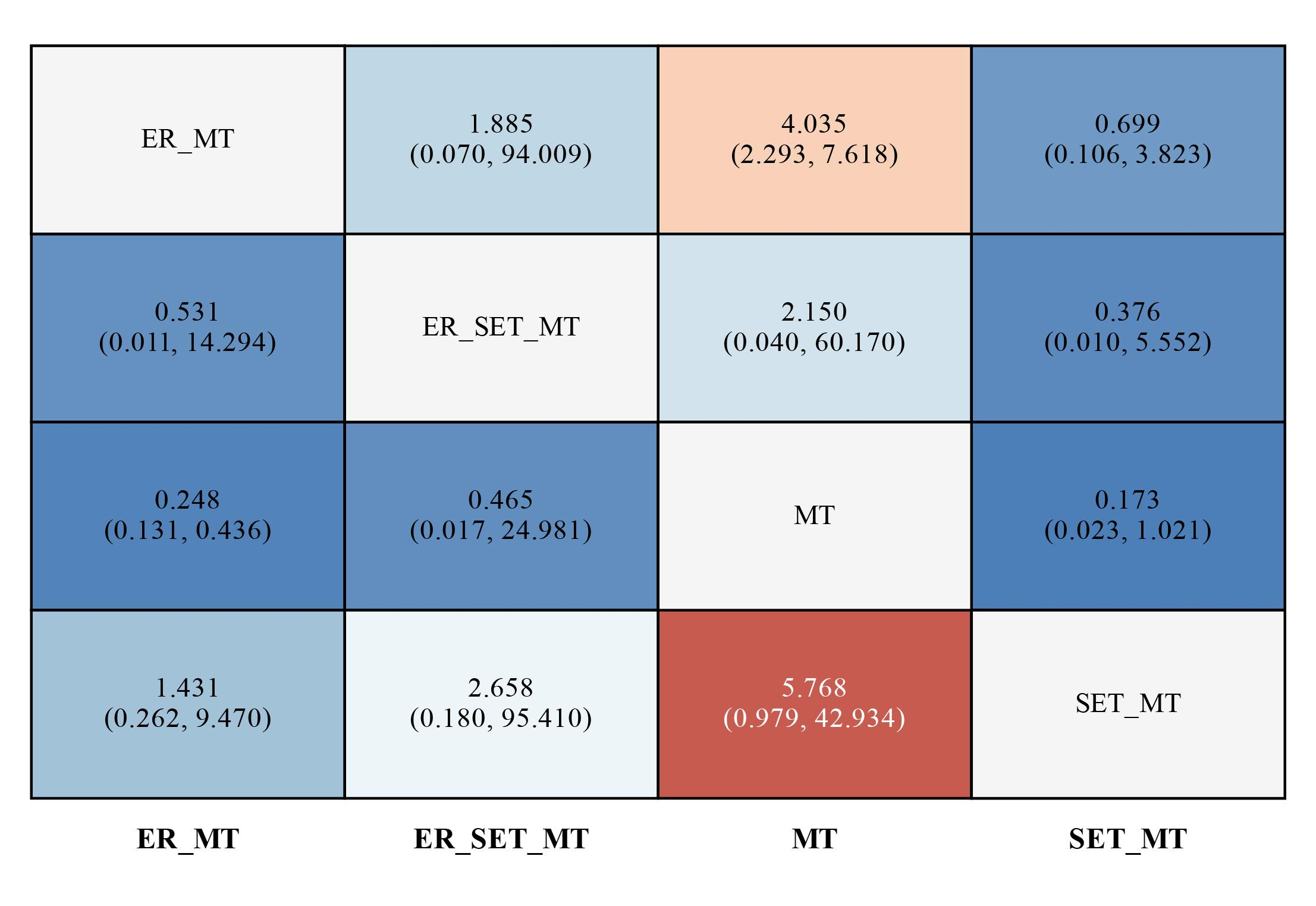

Supplement: Supplementary file 3 [file Data_Sheet_3.ZIP › Table/Table 5.tif]

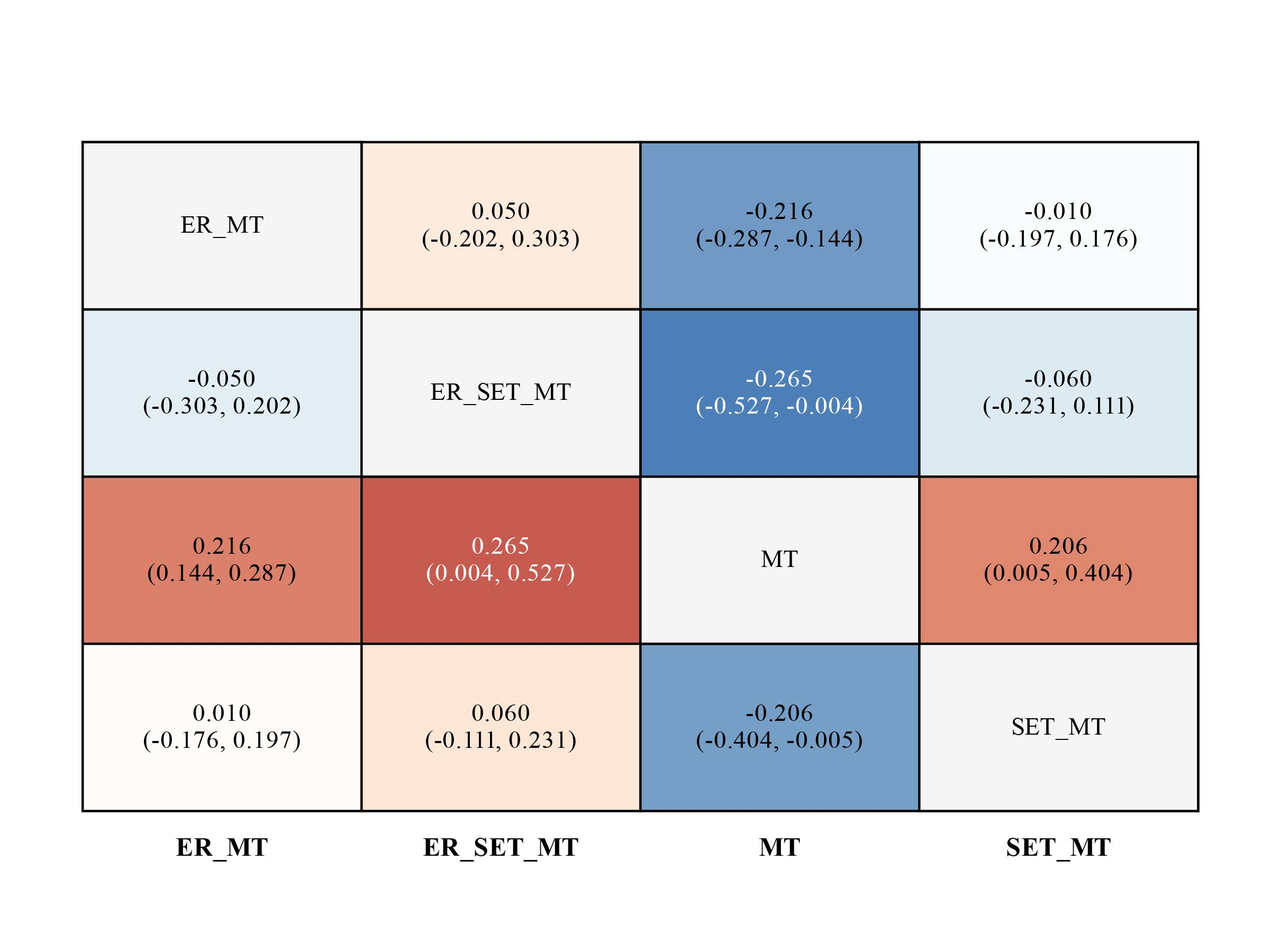

Supplement: Supplementary file 3 [file Data_Sheet_3.ZIP › Table/Table 6.tif]
